# Effect of a Novel Multicomponent Intervention to Improve Patient Access to Kidney Transplant and Living Kidney Donation: The EnAKT LKD Cluster Randomized Clinical Trial

**DOI:** 10.1001/jamainternmed.2023.5802

**Published:** 2023-11-03

**Authors:** Amit X. Garg, Seychelle Yohanna, Kyla L. Naylor, Susan Q. McKenzie, Istvan Mucsi, Stephanie N. Dixon, Bin Luo, Jessica M. Sontrop, Mary Beaucage, Dmitri Belenko, Candice Coghlan, Rebecca Cooper, Lori Elliott, Leah Getchell, Esti Heale, Vincent Ki, Gihad Nesrallah, Rachel E. Patzer, Justin Presseau, Marian Reich, Darin Treleaven, Carol Wang, Amy D. Waterman, Jeffrey Zaltzman, Peter G. Blake

**Affiliations:** 1 Division of Nephrology, Department of Medicine, McMaster University, Hamilton, Ontario, Canada; 2 Lawson Health Research Institute and London Health Sciences Centre, London, Ontario, Canada; 3 ICES, Ontario, Canada; 4 Department of Epidemiology and Biostatistics, Schulich School of Medicine and Dentistry, Western University, London, Ontario, Canada; 5 Kidney Patient & Donor Alliance, Canada; 6 Transplant Ambassador Program, Ontario, Canada; 7 Ajmera Transplant Centre, University Health Network, Toronto, Ontario, Canada; 8 Division of Nephrology, Department of Medicine, University of Toronto, Toronto, Ontario, Canada; 9 Patient Governance Circle, Indigenous Peoples Engagement and Research Council and Executive Committee, Can-Solve CKD, Vancouver, British Columbia, Canada; 10 Provincial Patient and Family Advisory Council, Ontario Renal Network, Toronto, Ontario, Canada; 11 Patient co-lead Theme 1–Improve a Culture of Donation, Canadian Donation and Transplantation Research Program, Edmonton, Alberta, Canada; 12 Division of Nephrology, University of Toronto, Toronto, Ontario, Canada; 13 Centre for Living Organ Donation, University Health Network, Toronto, Ontario, Canada; 14 Ontario Renal Network, Toronto, Ontario, Canada; 15 Trillium Gift of Life Network, Ontario Health, Toronto, Ontario, Canada; 16 Ontario Renal Network, Ontario Health, Toronto, Ontario, Canada; 17 Can-SOLVE CKD Network, Vancouver BC, Canada; 18 Trillium Health Partners, Mississauga, Ontario, Canada; 19 Department of Medicine, Humber River Regional Hospital, Toronto, Ontario, Canada; 20 Regenstrief Institute, Indianapolis, Indiana; 21 Department of Surgery, Division of Transplantation, Indiana University School of Medicine, Indianapolis; 22 Clinical Epidemiology Program, Ottawa Health Research Institute, Ottawa, Ontario, Canada; 23 School of Epidemiology and Public Health, University of Ottawa, Ontario, Canada; 24 Canadians Seeking Solutions and Innovations to Overcome Chronic Kidney Disease (Can-Solve CKD), Patient Council, Vancouver, British Columbia, Canada; 25 McMaster University, Hamilton, Ontario, Canada; 26 Division of Nephrology, Department of Medicine, Schulich School of Medicine and Dentistry, Western University, London, Ontario, Canada; 27 Department of Research Methods, Evidence and Uptake, Faculty of Health Sciences, McMaster University, Hamilton, Ontario, Canada; 28 Department of Surgery and J.C. Walter Jr. Transplant Center, Houston Methodist Hospital, Houston, Texas; 29 Division of Nephrology, St. Michael’s Hospital, Toronto, Ontario, Canada

## Abstract

**Importance:**

Patients with advanced chronic kidney disease (CKD) have the best chance for a longer and healthier life if they receive a kidney transplant. However, many barriers prevent patients from receiving a transplant.

**Objectives:**

To evaluate the effect of a multicomponent intervention designed to target several barriers that prevent eligible patients from completing key steps toward receiving a kidney transplant.

**Design, Setting, and Participants:**

This pragmatic, 2-arm, parallel-group, open-label, registry-based, superiority, cluster randomized clinical trial included all 26 CKD programs in Ontario, Canada, from November 1, 2017, to December 31, 2021. These programs provide care for patients with advanced CKD (patients approaching the need for dialysis or receiving maintenance dialysis).

**Interventions:**

Using stratified, covariate-constrained randomization, allocation of the CKD programs at a 1:1 ratio was used to compare the multicomponent intervention vs usual care for 4.2 years. The intervention had 4 main components, (1) administrative support to establish local quality improvement teams; (2) transplant educational resources; (3) an initiative for transplant recipients and living donors to share stories and experiences; and (4) program-level performance reports and oversight by administrative leaders.

**Main Outcomes and Measures:**

The primary outcome was the rate of steps completed toward receiving a kidney transplant. Each patient could complete up to 4 steps: step 1, referred to a transplant center for evaluation; step 2, had a potential living donor contact a transplant center for evaluation; step 3, added to the deceased donor waitlist; and step 4, received a transplant from a living or deceased donor.

**Results:**

The 26 CKD programs (13 intervention, 13 usual care) during the trial period included 20 375 potentially transplant-eligible patients with advanced CKD (intervention group [n = 9780 patients], usual-care group [n = 10 595 patients]). Despite evidence of intervention uptake, the step completion rate did not significantly differ between the intervention vs usual-care groups: 5334 vs 5638 steps; 24.8 vs 24.1 steps per 100 patient-years; adjusted hazard ratio, 1.00 (95% CI, 0.87-1.15).

**Conclusions and Relevance:**

This novel multicomponent intervention did not significantly increase the rate of completed steps toward receiving a kidney transplant. Improving access to transplantation remains a global priority that requires substantial effort.

**Trial Registration:**

ClinicalTrials.gov Identifier: NCT03329521

## Key Points

**Question:**

Does a novel multicomponent intervention help potentially transplant-eligible patients with advanced chronic kidney disease (CKD) complete key steps toward receiving a kidney transplant?

**Findings:**

In this cluster randomized clinical trial that included 20 375 patients from 26 CKD programs, the intervention compared with usual care did not significantly increase the rate of steps completed toward receiving a kidney transplant.

**Meaning:**

Among transplant-eligible patients with advanced CKD, this multicomponent intervention, which targeted many barriers to receiving a kidney transplant, did not significantly increase overall access to kidney transplant.

## Teaser

This randomized clinical trial evaluates the effect of implementing a novel multicomponent intervention designed to target several barriers that prevent eligible patients from completing key steps toward receiving a kidney transplant.

## Introduction

Patients with kidney failure need ongoing dialysis treatments or a kidney transplant to survive. Compared with dialysis, a kidney transplant offers patients a better quality of life and many gain 10 or more years of life expectancy.^1^ A transplant also costs the health care system less—over 5 years, every 100 kidney transplants save the health care system about $14.6 million USD (Canadian data), driven by averted dialysis costs.^2^ Living donor transplants offer further advantages compared with deceased donor transplants, including superior graft and patient survival.^3^ Unfortunately, many eligible patients will never receive a kidney transplant.^4^

Reasons for this care gap are complex, and barriers exist for patients, families, health care professionals, chronic kidney disease (CKD) programs, transplant centers, and health care systems.^5^ Education is required on multiple occasions, for varied audiences, and in several formats.^6^ Many patients and staff need clarifications about the transplant and living donor process.^7^ It is not easy to discuss living donations with family and friends.^4^ Overall, CKD programs need to better coordinate with transplant centers.^8^ There are too few kidneys from deceased donors, and living donation rates remain stagnant.^9^ In Ontario, CKD program staff traditionally placed little emphasis on transplant activity in their day-to-day work.^5^

Initiatives have been launched in several countries to address these barriers.^10^ We conducted the Enhance Access to Kidney Transplantation and Living Kidney Donation (EnAKT LKD) trial to determine the effect of a novel multicomponent intervention designed to help eligible patients complete key steps toward receiving a kidney transplant.

## Methods

### Study Design

We conducted a pragmatic, 2-arm, parallel-group, open-label, registry-based, superiority, cluster-randomized clinical trial. We designed the intervention to affect entire CKD programs (the clusters), so program-level randomization was adopted for logistical reasons and to minimize cross-group contamination. Trial data came mainly from linked administrative health care databases, including dialysis and transplant registries. We published the protocol (Supplement 1) and statistical plan (Supplement 2) before the outcome analysis, and changes to the protocol prior to this analysis are documented at ClinicalTrials.gov Identifier: NCT03329521.^5,11^ The trial was designed, analyzed, and reported following recommended guidelines (eTables 1-4 in Supplement 3).^12,13,14^ The Health Sciences Research Ethics Committee of Western University approved the trial to use a waiver of patient consent for participation (REB #108408).

### Study Setting and Participants

The administrative leaders of all 26 CKD programs in Ontario, Canada (the clusters) agreed to include their program and receive the randomly assigned intervention during the 4.2-year trial period from November 1, 2017, to December 31, 2021. Together these programs treat more than 24 000 patients with advanced CKD (patients approaching the need for dialysis or receiving maintenance dialysis; about half are potentially transplant eligible) each year.^15^ The trial was embedded in routine care across 26 specialized kidney clinics (for patients approaching the need for dialysis), 26 home dialysis programs, and 97 hemodialysis units. More than 3400 nurses and 230 nephrologists provided this care.

The CKD program staff educate patients and families on their treatment options, including transplant. Staff are also responsible for sending referrals to 1 of 6 transplant centers in Ontario. A referral requires the results of many tests (eg, blood tests, cardiac assessment, abdominal imaging; the complete list is described elsewhere^16^); note, this process differs in the US, where either a CKD program forwards a patient’s contact information to a transplant center to make the referral or a patient contacts the transplant center directly (ie, self-referral).^17^ Interested potential donors contact a transplant center to receive more information and begin their evaluation.

We used administrative data to identify and follow up patients with advanced CKD who received care in the 26 CKD programs. Data sets were authorized for use in this trial under section 45 of Ontario’s Personal Health Information Protection Act, linked using unique encoded identifiers, and analyzed at ICES.^18^ Patients could enter the trial on November 1, 2017, or during the trial period. Patients could only enter the trial once (eg, patients who received a kidney transplant during the trial that later failed did not re-enter). We followed patient outcomes until December 31, 2021, and patient entry into the trial analysis ended on September 30, 2021, to allow for at least 90 days of potential follow-up.

Entering the trial refers to patients being included in the outcome analyses, and the analyses were performed using linked administrative health care databases after the trial period was over. To enter the trial, patients needed to be aged 18 to 75 years (kidney transplants after age 75 years are rare).^15^ Patients also needed to be potentially transplant-eligible (have no recorded contraindications in the data sources; for example, no record of dementia, a long-term care residence, use of home oxygen [a sign of severe lung disease], or comorbidities likely to preclude transplant).^15^ Patients approaching the need for dialysis received care in specialized kidney clinics staffed by multidisciplinary teams. Clinic patients entered the trial when there was the first evidence of (1) an estimated glomerular filtration rate (eGFR) lower than 15 mL/min per 1.73 m^2^ (calculated using the 2021 equation without race)^19^ or (2) a 25% or greater 2-year predicted risk of kidney failure (calculated using the kidney failure risk equation [KFRE]).^20^ To ensure stable kidney function, at least 2 eGFR or 2 KFRE measures were required to enter the trial, with measures separated by at least 90 days but no more than 365 days. The remaining patients entered the trial after evidence of receiving outpatient maintenance dialysis in a center or at home.

### Randomization

We used covariate-constrained randomization, stratified by historic transplant center referral patterns, to allocate the 26 CKD programs (1:1) to the intervention or usual-care groups.^5^ More details are in the trial protocol (Supplement 1).^5^ Briefly, we concealed the process of computer generating the random allocation scheme from all CKD programs and most trial team members. Each program received notification of its group assignment approximately 9 months before the trial start date and prepared for implementation. All programs began the 4.2-year trial period on November 1, 2017.

### Intervention

Details are in the protocol, which features a recommended template to describe a complex intervention.^5,21,22^ The multicomponent intervention was codesigned by an 18-member panel to address complex barriers at multiple levels that prevent kidney transplant and living donation.^4^ The panel had expertise in CKD, transplant, quality improvement, health care data, education, and patient support and included patients, health care staff, administrators, scientists, and representatives from CKD programs, transplant centers, and kidney and transplant government-funded agencies.^23,24^ Four main components were developed for the intervention and provided to each CKD program. Instructions were given not to share intervention resources with the usual-care group (to prevent between-group contamination); however, resources were made broadly available after the trial.

#### Administration

Support from a central operations group (a part-time medical lead and 3 full-time personnel [manager, analyst, strategist]) to establish a new local quality improvement team to drive local performance. Each team received $7316 per year to support their activities. In addition, team representatives were invited to new monthly provincial rounds to share experiences and strategies (most were 1-hour teleconferences; 4 were 1-day in-person conferences).

#### Education

Multiple educational resources were made available to health care staff, patients, and potential donors.^25^ One resource^26^ was the mass production and online creation of a personalized education and coaching program (Explore Transplant Ontario^27^).

#### Patient Support

A new initiative of more than 85 kidney transplant recipients and living kidney donors who voluntarily spend time in CKD programs, sharing their experiential knowledge and stories and providing emotional support and hope.46

#### Data and Accountability

Program-level reports summarizing trends in 13 transplant measures, distributed quarterly to local teams and annually for 1-on-1 performance meetings between leaders of each CKD program and the provincial kidney agency.^28^

### Outcomes

The primary outcome was the rate of steps completed toward receiving a kidney transplant. Each patient could complete up to 4 steps, and for each patient, we only counted each step once: step 1, referred to a transplant center for evaluation; step 2, had a potential living donor contact a transplant center for evaluation (only the first donor counted if multiple donors began evaluations); step 3, added to the deceased donor waitlist; and step 4, received a transplant from a living or deceased donor.

The average wait time for a deceased donor kidney in Ontario is 4 years,^29^ and this intervention was not designed to increase the number of kidneys available from deceased donors. Thus, 5 secondary outcomes focused on specific steps, or a combination of steps, for living donor transplant (1) donor evaluation or living donor transplant (step 2 or a subset of step 4), (2) donor evaluation (step 2), (3) referral and donor evaluation (steps 1 and 2), (4) living donor transplant (a subset of step 4), and (5) preemptive living kidney donor transplant (a subset of step 4).

### Sample Size Calculation

Details are in the trial protocol and statistical plan (Supplement 1 and Supplement 2).^5,11^ Accounting for the correlation within clusters, the trial was designed to have at least 80% power to detect a between-group difference of 12 steps per 100 patient-years (assumed 23 steps per 100 patient-years in the usual-care group, 2-sided α = .05).

### Statistical Analysis

Details are in the statistical plan (Supplement 2).^11^ We performed the analyses using SAS statistical software (version 9.4; SAS Institute, Inc) and R statistical software (version 3.6, R Foundation).^30^ The analysis was performed between October 2022 and March 2023, after outcome data for the entire trial period became available in the data sources used.

Almost all baseline data were complete (eTable 5 in Supplement 3). For outcome data, 2.1% of patients had evidence of a missing step in error (how this missing data was handled is presented in eTable 6 in Supplement 3).

The analyses used an intention-to-treat approach, meaning patients were analyzed according to the random assignment of their CKD program when they entered the trial (intervention group vs usual-care group), regardless of whether they transferred to a CKD program in the alternate group. After trial entry, the only reason for patient loss to follow-up was emigration from the province (which occured in <2.0% of patients with advanced CKD each year). Otherwise, for analysis of the primary outcome, a patient’s observation time was stopped if they reached the trial end date (December 31, 2021), died, received a kidney transplant, recovered kidney function, or became ineligible to receive a transplant (developed a recorded contraindication as described elsewhere,^11^ except those patients who older than 75 years in follow-up remained in the trial).

Outcomes were analyzed at the patient level using stratified, constrained, multistate models, accounting for the order of step completion, the clustered design, and the covariates used in the randomization (shown visually in eFigures 1A and 1B in Supplement 3).^31^ When an outcome was a single step (eg, receipt of a living donor transplant), the multistate model reduced to a classic Cox proportional hazards model. We used cluster-level bootstrapping to obtain standard errors (accounting for the correlated outcomes within CKD programs) and a *t*-distribution as a small-sample correction for fewer than 40 clusters. eTable 7 in Supplement 3 shows the intracluster correlation coefficient (ICC) and coefficient of variance measures for the outcomes. Historic transplant center referral patterns were a stratification factor in the randomization and the model. We also stratified for the different transitions between steps to allow for different baseline transition intensities. The models accounted for the covariates used in the randomization and other key characteristics including age, sex, Charlson Comorbidity Index, 1 or more intensive care unit admissions in the prior year, frequency of hospital admissions in the prior year, historical rate of transplant at the CKD program, presence of a transplant center with the CKD program (present in 6 of the 26 CKD programs), and the CKD treatment modality at the time of trial entry (ie, in-center hemodialysis, home dialysis, or approaching the need for dialysis). Violation of the Markov assumption meant 3 event history variables were also included in model adjustment (details in eTable 8 in Supplement 3). The constrained models provided single estimates of the relative rate (hazard ratio) of each outcome among patients in CKD programs in the intervention group vs the usual-care group. Terminating events were treated as censoring terms in the analysis, resulting in a cause-specific hazard ratio for the total effect of the intervention. We expected most patients would enter the trial without steps completed toward receiving a transplant; those with prior steps only had new steps during the trial counted in the outcome.

Following prespecified α spending criteria,^5,11^ once we spent α, there was no allowance for multiplicity, and inferences may not be reproducible; we reported no other *P* values, and all outcomes are described with 95% CIs unadjusted for multiple testing.

In prespecified additional analyses, we investigated the consistency of the estimated intervention effect in models that were (1) unadjusted for characteristics, (2) adjusted for additional baseline characteristics, and (3) unconstrained to examine intervention effects on each transition shown visually in eFigures 1A and 1B in Supplement 3. The intervention effect was also assessed on steps not specified as secondary outcomes (kidney transplant [from a living or deceased donor], deceased donor transplant, referral, and waitlisting); in follow-up truncated to March 16, 2020, the date when nearly all kidney transplant activity in Ontario stopped due to the COVID-19 pandemic^32^; and in multiple subgroups.^5,11^ In another prespecified analysis, times to step completion were examined separately in each group along with other measures.

In post hoc analyses, we investigated the consistency of the estimated intervention effect in patients who (1) completed no steps toward receiving a transplant before trial entry, (2) entered the trial approaching the need for dialysis, and (3) were receiving maintenance dialysis when they entered the trial.

In a post hoc analysis to assess potential contamination bias, we compared kidney transplant rates in the usual-care group during the trial to historical norms. If programs in the usual-care group inadvertently gained access to intervention materials, kidney transplant rates may have increased.

## Results

During the 4.2-year trial period, the 26 CKD programs cared for 20 375 patients with advanced CKD who were potentially transplant eligible. A total of 9780 patients entered the trial from 13 CKD programs in the intervention group and 10 595 patients from 13 CKD programs in the usual-care group. We included all patients in the intention-to-treat analysis (Figure).

**Figure.**
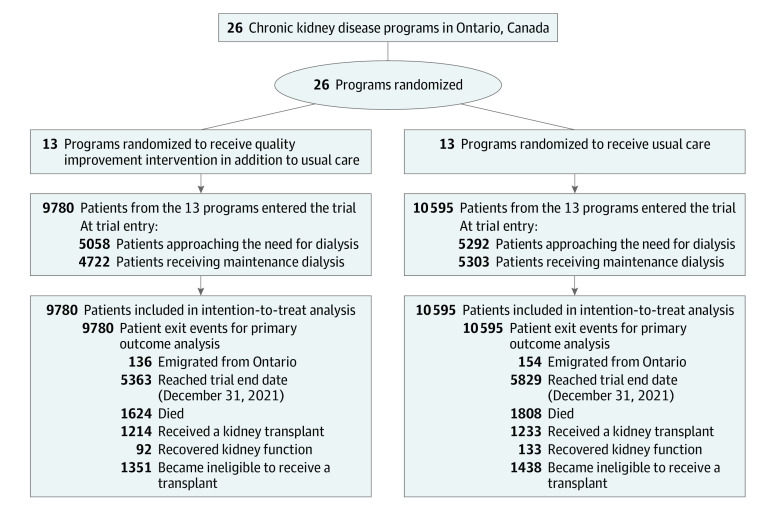
Participant Flow in the EnAKT LKD Trial

### Center and Patient Characteristics

Center and patient characteristics were similar between the 2 groups (Table 1). For the 20 375 patients, the median (IQR) age was 61 (51-69) years, 7786 were women (38%), and 11 517 had a history of diabetes (57%). When they entered the trial, 10 025 patients (51%) were approaching the need for dialysis and the remaining 49% were receiving maintenance dialysis. For patients who entered the trial approaching the need for dialysis, the median (IQR) estimated glomerular filtration rate was 16 (13-20) mL/min per 1.73 m^2^, the median (IQR) random urine albumin-to-creatinine ratio was 162 (69-314) mg/mmol, and the 2-year predicted risk of kidney failure was 45% (32%-67%). For patients who entered the trial on November 1, 2017, already receiving maintenance dialysis, the median (IQR) duration of dialysis was 2.6 (1.2-5.1) years. Before entering the trial, 80% of patients had completed no steps toward receiving a kidney transplant. Of the patients who entered the trial approaching the need for dialysis, 48% started maintenance dialysis during the trial.

**Table 1.** Baseline Characteristics

Characteristic	Intervention	Usual care
CKD program characteristics		
No. of programs	13	13
No. of patients per program who entered the trial, median (IQR)	789 (535-912)	738 (591-995)
Transplant center colocated with CKD program, No.	3	3
Historical rate of kidney transplant, 100 patient-years, mean (SD)^a^	6.78 (0.43)	6.31 (0.73)
Historical rate of living kidney donor transplant	1.83 (0.17)	1.91 (0.30)
Historical rate of deceased kidney donor transplant	4.95 (0.35)	4.40 (0.49)
Patient characteristics		
Patients, No.	9780	10 595
Entered the trial approaching the need for dialysis, No. (%)^b^	5058 (51.7)	5292 (49.9)
Estimated glomerular filtration rate, median (IQR), mL/min per 1.73 m^2^	16 (13-21)	16 (13-20)
Random urine albumin to creatinine ratio, median (IQR), mg/mmol	157 (66-301)	168 (72-322)
Estimated 2-y risk of kidney failure, median (IQR), %	43 (31-65)	46 (32-69)
Entered the trial receiving maintenance dialysis, No. (%)	4722 (48.3)	5303 (50.1)
Entered the trial receiving in-center hemodialysis	3495 (35.7)	3951 (37.3)
Entered the trial receiving home dialysis	1227 (12.5)	1352 (12.8)
Entered the trial on November 1, 2017, No. (%)	4162 (42.6)	4560 (43.0)
Entered the trial approaching the need for dialysis	1114 (11.4)	1246 (11.8)
Entered the trial receiving maintenance dialysis	3048 (31.2)	3314 (31.3)
Duration of dialysis, median (IQR), y	2.6 (1.2-5.2)	2.6 (1.1-5.0)
Entered the trial after November 1, 2017	5618 (57.4)	6035 (57.0)
Entered the trial approaching the need for dialysis	3944 (40.3)	4046 (38.2)
Entered the trial receiving maintenance dialysis	1674 (17.1)	1989 (18.8)
Prior kidney transplant, No. (%)	106 (1.1)	93 (0.9)
Age, median (IQR), y	61 (52-69)	61 (51-69)
Age, No. (%), y		
18-45	1395 (14.3)	1531 (14.5)
45-54	1646 (16.8)	1770 (16.7)
55-64	2805 (28.7)	3066 (28.9)
65-75	3934 (40.2)	4228 (39.9)
Sex, No. (%)		
Female	3726 (38.1)	4060 (38.3)
Male	6054 (61.9)	6535 (61.7)
Rural residence (population <10 000), No. (%)	1091 (11.2)	1328 (12.5)
Lower income, No. (%)^c^	5042 (51.6)	5343 (50.4)
Ontario Marginalization Index, No. (%)^d^		
Higher residential instability	4899 (50.1)	4633 (43.7)
Higher material deprivation	4746 (48.5)	5109 (48.2)
Higher ethnic diversity	5089 (52.0)	5289 (49.9)
Higher dependency	3628 (37.1)	4013 (37.9)
One-way distance from residence to transplant center, median (IQR), km	25 (11-55)	32 (16-81)
Body mass index, No./total No. (%)^e^		
<25	2400/8777 (27.3)	2783/9741 (28.6)
25-29.9	2683/8777 (30.6)	2922/9741 (30.0)
30-34.9	1850/8777 (21.1)	2024/9741 (20.8)
≥35	1844/8777 (21.0)	2012/9741 (20.7)
Charlson Comorbidity Index, No. (%)^f^		
0	3802 (38.9)	3986 (37.6)
1	2263 (23.1)	2547 (24.0)
2	1235 (12.6)	1285 (12.1)
3	1275 (13.0)	1461 (13.8)
≥4	1205 (12.3)	1316 (12.4)
Comorbidities, No. (%)		
Atrial fibrillation or flutter	1543 (15.8)	1774 (16.7)
Chronic liver disease	1047 (10.7)	1222 (11.5)
Chronic obstructive pulmonary disease	1838 (18.8)	1966 (18.6)
Congestive heart failure	2726 (27.9)	3038 (28.7)
Coronary artery disease	3213 (32.9)	3538 (33.4)
Depression	822 (8.4)	983 (9.3)
Diabetes mellitus	5390 (55.1)	6127 (57.8)
Hypertension	8633 (88.3)	9256 (87.4)
Lower extremity amputation	239 (2.4)	306 (2.9)
Myocardial infarction	722 (7.4)	803 (7.6)
Peripheral vascular disease	584 (6.0)	715 (6.7)
Previous skeletal fracture	500 (5.1)	501 (4.7)
Transient ischemic attack or stroke	1164 (11.9)	1245 (11.8)
Venous thromboembolism	119 (1.2)	149 (1.4)
Healthcare utilization		
Hospital admissions in prior year, No. (%)		
None	5292 (54.1)	5797 (54.7)
1	2476 (25.3)	2624 (24.8)
2	1120 (11.5)	1146 (10.8)
≥3	892 (9.1)	1028 (9.7)
Emergency department visits in prior year, No. (%)		
None	3706 (37.9)	3913 (36.9)
1	2310 (23.6)	2477 (23.4)
2	1411 (14.4)	1523 (14.4)
≥3	2353 (24.1)	2682 (25.3)
Intensive care unit admission in prior year, No. (%)	1335 (13.7)	1490 (14.1)
Intensive care unit admission with mechanical ventilation in prior year, No (%)	14 (0.1)	14 (0.1)

Abbreviation: CKD, chronic kidney disease.

^a^ Historic transplant rates were calculated in a similar fashion as done during the trial period. Patients with advanced CKD (patients approaching the need for dialysis or receiving maintenance dialysis) were accrued on November 1, 2013, or during the period ending September 30, 2017, with a maximum follow-up of October 31, 2017. The incidence rate was calculated as the number of transplants over the patient-years. We used cluster-level bootstrapping to obtain standard deviations, to account for correlated outcomes within CKD programs.

^b^ In the intervention group, 2375 of 5058 patients (47.0%) started maintenance dialysis during the trial period; in the usual-care group, 2602 of 5292 patients (49.2%) started maintenance dialysis during the trial period.

^c^ Lower income was defined as living in a neighborhood in the bottom 2 quintiles of average neighborhood income in the province.

^d^ The Ontario Marginalization Index (ON-MARG) is categorized in 5 equal parts (quintiles) for all neighborhoods in the province. The ON-MARG is a Census (geographical)-based index that quantifies the degree of marginalization across Ontario using 4 dimensions: residential instability, material deprivation, ethnic diversity, and dependency. For each dimension, higher marginalization was defined as living in a neighborhood in the top 2 quintiles. Residential instability refers to the area-level concentration of people with high housing instability or family instability. Material deprivation refers to individuals and communities not being able to access and realize basic material needs. Ethnic diversity refers to area-level concentrations of residents who are recent immigrants and/or those who self-identify as a visible minority. Dependency refers to the area-level concentrations of people who do not have employment income, including seniors and children. The Ontario Community Health Profiles Partnership was the source of the marginalization indices.

^e^ BMI calculated as weight in kilograms divided by height in meters squared. Height and weight were recorded at the time patients were registered into the provincial kidney reporting system and was missing in 1003 patients (10.3%) in the intervention group and 854 patients (8.1%) in the usual-care group.

^f^ The Charlson Comorbidity Index is a weighted index that can be used to assess comorbidity and predict survival in patients with kidney disease. A higher score represents greater comorbidity.

We followed up patients for a median (IQR) of 2.1 (1.0-3.6) years. During follow-up, 290 patients (1.4%) emigrated from the province, 225 recovered their kidney function (1.1%), 2789 became ineligible to receive a kidney transplant (13.7%), and 3432 died (16.8%); these rates were similar between the 2 groups (Figure**;** eTable 9 in Supplement 3). In follow-up, 1105 patients (5.4%) (484 [4.9%] intervention, 621 [5.9%] usual care) transferred to a CKD program in the alternate group (eTable 9 in Supplement 3). In the trial, patients spent 97.2% of all follow-up time receiving care in a CKD program per the random allocation.

### Intervention Uptake

The provincial core operations group met more than 100 times during the trial and observed evidence of intervention uptake. Each of the 13 CKD programs established a local quality improvement team, developed a charter with their goals and activities, and indicated that team members met regularly to review and improve transplant performance. Representatives from each local team participated in monthly provincial rounds. Together, the programs reported that 1740 patients completed the educational program Explore Transplant Ontario. Transplant ambassadors (prior kidney transplant recipients and living kidney donors) recorded 5471 meaningful interactions with patients with advanced CKD and 719 meaningful interactions with potential living kidney donors. Each CKD program received regular performance reports and had annual performance meetings with the provincial kidney agency.

The onset of the COVID-19 pandemic, 2.4 years into the 4.2-year trial period, substantially affected intervention delivery for at least a year. Transplant activity ceased temporarily, local quality improvement teams met less often, there was a pause on the provincial rounds, health care staff retired or were redeployed, and transplant ambassadors transitioned from in-person to virtual meetings.

### Primary Outcome

The rate of the primary outcome did not differ significantly between the intervention group [n = 9780 patients] vs the usual-care group [10 595 patients]: 5334 vs 5638 steps; 24.8 vs 24.1 steps per 100 patient-years; adjusted hazard ratio 1.00 (95% CI, 0.87-1.15) (Table 2**;** eFigures 1A and 1B in Supplement 3; the Video [animation of step completion over the trial period]). In follow-up, 131 patients (1.3%) in the intervention group completed all 4 steps, and 572 (5.8%), 1493 (15.3%), and 3138 (32.1%) completed 3 or more, 2 or more, and 1 or more steps, respectively. Corresponding numbers in the usual-care group were 129 (1.2%), 589 (5.6%), 1538 (14.5%), and 3382 (31.9%), respectively.

**Table 2.** Effect of the Quality Improvement Intervention on the Rate of Steps^a^ Completed Toward Receiving a Kidney Transplant

Variable	Patients, No.		Steps completed during the trial, No.		Rate of steps completed per 100 patient-years (95% CI)^b^		Adjusted hazard ratio (95% CI)^c^	*P* value^d^
	Intervention	Usual care	Intervention	Usual care	Intervention	Usual care		
Primary outcome								
Steps 1, 2, 3, or 4	9780	10 595	5334	5638	24.8 (21.8-27.9)	24.1 (19.7-28.4)	1.00 (0.87-1.15)	.96
Secondary outcomes								
Steps 2 or 4, restricted to living donor transplants	9780	10 595	1304	1299	6.1 (5.0-7.2)	5.5 (3.9-7.2)	1.11 (0.89-1.38)	NA
Step 2^e^	9210^f^	10 029^f^	923	920	4.9 (4.1-5.6)	4.4 (3.1-5.7)	1.22 (0.97-1.54)	NA
Steps 1 and 2^e^	7828^g^	8541^g^	677	691	4.2 (3.4-5.0)	3.9 (2.7-5.1)	1.16 (0.89-1.50)	NA
Step 4, restricted to living donor transplants^e^	9780	10 595	381	379	1.8 (1.3-2.3)	1.6 (1.0-2.2)	1.06 (0.70-1.60)	NA
Step 4, restricted to preemptive living donor transplants^e^^,^^h^	5058^i^	5292^i^	127	115	1.7 (1.1-2.4)	1.5 (0.6-2.4)	1.11 (0.57-2.15)	NA

Abbreviation: NA, not applicable.

^a^ Steps (1) referred to a transplant center for evaluation; (2) had a potential living donor contact a transplant center for evaluation (only the first potential donor was counted for a patient when there were multiple potential donors); (3) added to deceased donor waitlist; and (4) received a transplant from a living or deceased donor.

^b^ For 95% CIs, we used cluster-level bootstrapping to obtain standard errors (accounting for the correlated outcomes within chronic kidney disease programs) and a *t*-distribution as a small-sample correction when calculating the margin of error in the 95% CI because our trial included fewer than 40 clusters.

^c^ For the adjusted hazard ratio, the referent group was usual care. The primary outcome was analyzed at the patient-level using a stratified, constrained, multistate model accounting for the order in which steps were completed, the clustered design, and the covariates used in the randomization. See the Statistical Analysis section in the Methods for more details.

^d^ The level of significance for the primary outcome was α = .05. As α was spent up in the analysis of the primary outcome, subsequent statistical tests are reported as point estimates with 95% CIs without *P* values (NA).

^e^ The multistate model for this outcome reduced to a classic Cox proportional hazards model.

^f^ Excludes patients who completed step 2 before entering the trial.

^g^ Excludes patients who completed steps 1 or 2 before entering the trial.

^h^ The follow-up time was censored if and when a patient started dialysis.

^i^ Excludes patients who were receiving maintenance dialysis when they entered the trial.

**Video.** Animation of Step Completion in the Enhance Access to Kidney Transplantation and Living Kidney Donation Cluster-Randomized Clinical Trial This video shows the rate that each step was completed over time in both study groups.

### Secondary Outcomes

There was no notable between-group difference in the 5 secondary outcomes focused on living donor transplant, as each hazard ratio 95% CI contained a value of 1.0 (Table 2). The rate of starting a living donor evaluation (step 2) for patients with CKD who did not have one before trial entry was numerically higher but not notably different between the intervention group (n = 9210 patients) vs the usual-care group (10 029 patients): 923 vs 920 evaluations; 4.9 vs 4.4 evaluations per 100 patient-years; adjusted hazard ratio, 1.22 (95% CI, 0.97-1.54) (Table 2).

### Additional Analyses

The results of 6 prespecified analyses to investigate the consistency of the estimated intervention effect are shown in eTables 10 to 14, eFigure 2 in Supplement 3. In these analyses, the 95% CIs of all hazard ratios contained a value of 1.0, indicating no notable difference between the groups. The exception was patients transitioning from no steps to having a potential living donor begin their evaluation (step 2), which was higher in the intervention group [n = 7828 patients] vs the usual-care group [8541 patients]: 347 vs 206 evaluations; 2.5 vs 1.4 evaluations per 100 patient-years; adjusted hazard ratio, 1.79 (95% CI, 1.02-3.17) (eTable 11 in Supplement 3).

eTable 15 in Supplement 3 shows each group’s time to complete specific steps and other measures. For example, the median (IQR) time from transplant referral to a living donor transplant (for those who completed both steps in follow-up) was 13.6 (9.3-19.3) months in the intervention group and 15.6 (11.3-20.9) months in the usual-care group. The median (IQR) time from when a potential living kidney donor began their evaluation to receipt of a living donor transplant (for those who completed both steps in follow-up) was 13.8 (9.5-18.5) months in the intervention group and 13.9 (9.6-19.1) months in the usual-care group.

### Post Hoc Analyses

The results of 3 post hoc analyses to investigate the consistency of the estimated intervention effect are shown in eTables 16 to 18 in Supplement 3. The 95% CIs of all hazard ratios contained a value of 1.0. In our assessment of potential contamination bias, transplant rates in the usual-care group were not higher during the trial compared with historical norms (eTable 19 in Supplement 3).

## Discussion

In this trial of more than 20 000 patients cared for by more than 3600 health care staff in 26 CKD programs, delivering a novel multicomponent intervention compared with usual care did not significantly increase the rate of steps completed toward receiving a kidney transplant. Trial results were consistent in multiple analyses.

The 2019 Advancing American Kidney Health executive order included ambitious goals for increasing the rate of kidney transplant.^10,33^ Although setting aspirational targets is helpful, our experience conducting this trial underscores the challenge of improving patient access to kidney transplant in busy health care environments. Effective solutions are urgently needed. A more resource-intensive intervention may be required to increase patient access to transplant; currently, however, there is limited evidence and guidance on effective strategies to increase access to kidney transplant. In prior trials,^34,35,36^ interventions that focused on transplant education, engagement activities, navigating the health care system, and/or performance reporting to dialysis centers have demonstrated mixed success in better knowledge, communication, and/or step completion in the transplant and living donation process. In our trial, the estimated rate of living donor evaluations was 22% higher in the intervention group than the usual-care group, with a 95% CI spanning a 3% decrease to a 54% increase in the rate of evaluations (the CI width would be broader if we accounted for multiple statistical comparisons).

The strengths of this clinical trial include its size, scope, and systematic approach to designing, implementing, and testing a province-wide multicomponent intervention. Diverse stakeholders, including administrators, health care staff, patients, and nephrologists, worked together to develop and implement the intervention, which was designed to address known barriers to receiving a transplant.^4,37,38,39^ Educational materials were created and used, and patients engaged with a new provincial support group of transplant recipients and living donors. The creation of data-sharing agreements and data collection allowed CKD programs to receive detailed performance reports for the first time. We conducted this pragmatic, randomized clinical trial within a learning health care system to generate robust estimates of how the intervention performed in a routine care setting.^40^

Nonetheless, it was disappointing that, overall, we could not demonstrate a significant benefit of this intervention compared with usual care. It is unlikely that contamination, which is the use of intervention components by the usual-care group during the trial, is a reason for these findings. We acknowledge that it takes time to improve health care delivery and organize each transplant, and CKD programs faced many challenges during the COVID-19 pandemic. We are now completing a multimodal process evaluation to understand how the intervention can be improved.^22^ In the interim, we identified 2 staff-related opportunities. First, although many staff in CKD programs appreciated the support and resources, they indicated that growing patient-to-staff ratios meant they lacked time to enact this initiative fully. Acknowledging this concern, the provincial kidney agency began providing annual funding for transplant coordination activities in all CKD programs after the trial was completed. Second, there was substantial turnover among frontline nurses and members of the quality improvement teams. Any future strategy should plan for an ongoing need for intervention reorientation.

### Limitations

This study has several limitations. The COVID-19 pandemic negatively affected intervention delivery, and the momentum for this initiative may have lessened as health care priorities shifted to focus on the pandemic. We did not determine whether the intervention improved culture, knowledge, or experience. Administrative data to assess transplant eligibility will never replace detailed in-person evaluation.^15^ Trial findings may not be generalizable to other health care systems because dialysis practice patterns, patient case-mix, access to multidisciplinary care, and resources available to improve access to kidney transplant may vary across geographical regions.^41,42,43,44^ Finally, this intervention did not address inequities in access to transplantation, a key priority in health care.^35,45,46,47^

## Conclusions

The findings of this randomized clinical trial did not show that a novel multicomponent intervention significantly increased the rate of completed steps toward receiving a kidney transplant. Improving access to transplantation remains a global priority that requires substantial effort.

## Notes

Supplement 1.
Trial Protocol

Supplement 2.
Statistical Analysis Plan

Supplement 3. **eTable 1.** Ottawa Statement **eTable 2.** CONSORT statement for cluster trials, pragmatic trials, and trials using routinely collected data **eTable 3.** Risk of bias **eTable 4.** CONSERVE-CONSORT (CONSORT Extension for RCTs Revised in Extenuating Circumstance) **eTable 5.** Missing baseline data **eTable 6.** Outcome ascertainment, data cleaning, and handling of missing data for the steps completed toward receiving a kidney transplant **eTable 7.** Intracluster correlation coefficient (ICC) and coefficient of variation measures for the outcomes **eTable 8.** Multistate model assumptions **eTable 9.** Patient follow-up **eTable 10.** Primary outcome examined in two multistate models: the first unadjusted for baseline characteristics and the second adjusted for additional baseline characteristics. **eTable 11.** Effect of the intervention on each transition (shown visually in eFigure 1a and 1b); these estimates are from an unconstrained model **eTable 12.** Effect of the intervention on steps not specified as primary or secondary outcomes **eTable 13.** Effect of the intervention when follow-up was truncated to March 16, 2020, the start of the COVID-19 pandemic in Ontario **eTable 14.** Effect of the intervention on the primary composite outcome in multiple subgroups **eTable 15.** The time to complete steps and other measures in the intervention group and usual-care group **eTable 16.** Effect of the intervention when restricted to patients who completed no steps toward receiving a transplant before trial entry **eTable 17.** Effect of the intervention when restricted to patients who entered the trial approaching the need for dialysis **eTable 18.** Effect of the intervention when restricted to patients who were receiving maintenance dialysis when they entered the trial **eTable 19.** Rates of kidney transplantation (total, living, deceased) in intervention and usual-care groups in pre-trial, trial, and pre-COVID-19 pandemic periods **eFigure 1.** Multistate models for the intervention and usual-care groups **eFigure 2.** Forest plot of the effect of the intervention on the primary composite outcome in multiple subgroups

Supplement 4.
Data Sharing Statement
