# Supplementary material for: Effect of a Novel Multicomponent Intervention to Improve Patient Access to Kidney Transplant and Living Kidney Donation: The EnAKT LKD Cluster Randomized Clinical Trial
Source: JAMA Intern Med. 2023 Nov 3;183(12):1366–75. doi: 10.1001/jamainternmed.2023.5802 (PMC10696487; doi:10.1001/jamainternmed.2023.5802)
Supplement: Supplement 1. — Trial Protocol [file jamainternmed-e235802-s001.pdf]

Title: Quality Improvement Intervention to Enhance Access to Kidney Transplantation and Living Kidney Donation (EnAKT LKD) in Patients with Chronic Kidney Disease: A Pragmatic, Registry-based, Cluster-Randomized Clinical Trial

Protocol Trial Registration: NCT03329521

Date: November 9th, 2022

Protocol Compilation

All documents are from the EnAKT LKD study.

The original protocol was approved by the research ethics board prior to randomization which occurred in February 2017 and prior to the Chronic Kidney Disease programs being notified of their assigned allocation in February 2017.

The final protocol was submitted for publication on December 20, 2020, and accepted for publication on January 2, 2021.

Summary of changes for the protocol were posted online on May 4, 2021, at ClinicalTrials.gov NCT03329521. It documents changes between the original protocol version on August 30, 2017, and the protocol published in the Canadian Journal of Kidney Health and Disease on January 2, 2021.

The original and only version of the statistical analysis plan was submitted for publication on June 23, 2022, and accepted for publication on September 5, 2022.

The original and only protocol for the process evaluation was approved by the research ethics board on October 15, 2020. The original and only version of the protocol for the process evaluation was submitted for publication on August 16, 2021 and accepted for publication on January 25, 2022.

Table of Contents

* The numbers below refer to the page numbers in the pdf document.

1. Original protocol…………………………………………………………………………………………………page 4
2. Final protocol……………………………………………………………………………………………………page 10
3. Table of protocol updates…………………………………………………………………………………..page 11
4. Final statistical analysis plan……………………………………………………………………………page 31
5. Final process evaluation protocol………………………………………………………………………page 32

A multicomponent quality improvement program to improve access to kidney transplantation and living kidney donation

Principal Investigator
Dr. Amit Garg MD, PhD
Schulich School of Medicine and Dentistry/Epidemiology & Biostatistics
London Health Sciences Centre
amit.garg@lhsc.on.ca

Lay summary of the study
Despite the advantages of kidney transplantation over dialysis (i.e., improved survival) only 42.5% of Canadians with end-stage renal disease have received a kidney transplant. The best way to improve access to kidney transplantation is unknown. A multicomponent kidney transplant quality improvement program was developed in collaboration with the Ontario Renal Network and Trillium Gift of Life Network to improve access to kidney transplantation in Ontario, Canada. The multicomponent program includes four main components: data (e.g., reports to renal programs about their program's performance), education (e.g., education toolkits), peer mentorship (e.g., kidney transplant recipients can mentor kidney disease patients), and administration (e.g., helping finance nursing staff time for transplant education). The goal of this multicomponent program is to increase kidney transplant referrals and kidney transplant rates in Ontario renal programs which care for individuals with kidney disease.

Study rationale
Compared to dialysis, kidney transplantation is associated with improved survival and quality of life with kidney transplantation saving an average of 4.4 life years per patient (1-3). Moreover, kidney transplantation has significantly lower healthcare system costs saving approximately $250,000 (CAD) per patient over a five-year period (4). However, despite these advantages, only 42.5% of the 41,931 Canadians with end-stage renal disease (ESRD) have received a kidney transplant (5). There are multiple reasons why ESRD patients who may benefit from a kidney transplant do not receive one with potential barriers to kidney transplantation existing at the level of the patient (e.g., patient does not have a good understanding of transplantation to make an informed decision about treatment options), potential living kidney donor (e.g., financial concern), healthcare provider (e.g., insufficient knowledge on transplantation), renal program (e.g., inefficient processes), and at the level of the transplant centre (e.g., lack of standard criteria for transplant acceptance). Moreover, many ESRD patients would like to receive a transplant but there are too few deceased donor organs available to meet demand (6). Although, patients can receive a kidney from a living donor there are also multiple barriers to living donation (e.g., difficulty finding a donor) (7). Recognizing the need to address barriers to kidney transplantation one of the Ontario Renal Network's (ORN) mandates is to improve access to kidney transplantation. To accomplish this, in collaboration with the Trillium Gift of Life Network (TGLN), the ORN plans to launch a multicomponent kidney transplant quality improvement program across the 26 renal programs in Ontario. Using a community-based participatory research approach and the best available evidence the multicomponent program was developed. The program will be comprised of four main components: 1. Data (e.g., provide

reports to renal programs about their performance using best practices in audit and feedback) (8); 2. Education (e.g., education toolkits for renal program providers, renal patients and families, and potential donors) (9-12); 3. Peer mentorship (e.g., kidney transplant recipients and living kidney donors can mentor individuals who are going through the transplant process to provide support) (13-15); and 4. Administration (e.g., helping finance nursing staff time for transplant education within each renal program). To our knowledge, this will be the first province in Canada to implement and evaluate a multicomponent kidney transplant quality improvement program to improve access to kidney transplantation with the goal of increasing the number of kidney transplant referrals and the number of kidney transplants.

Purpose and objectives

Purpose: Many pre-dialysis and dialysis patients would likely benefit from a kidney transplant, yet do not receive one. Recognizing this, one of the goals of the Ontario Renal Network (ORN), in collaboration with the Trillium Gift of Life Network (TGLN), is to improve access to kidney transplantation through implementing and evaluating a multicomponent kidney transplant quality improvement program across Ontario's regional renal programs and their associated multidisciplinary kidney care clinics.

Objectives: The primary objective is to determine if a multicomponent kidney transplant quality improvement program increases the kidney transplant referral rate in Ontario renal programs. Specifically, we will be comparing renal program level kidney transplant referral rates between the multicomponent quality improvement group and the standard-of-care group. We will also be examining several secondary outcomes, including: 1) proportion of potential living kidney donors contacting a transplant centre; 2) rate of kidney transplant evaluation; 3) rate of kidney transplant wait listing; 4) proportion of patients approved to receive a living kidney donor transplant; 5) rate of kidney transplantation (living and deceased donor kidney transplants examined separately); and 6) rate of pre-emptive kidney transplantation (i.e., no dialysis prior to transplantation). All secondary outcomes will also be examined at the level of the renal program.

Study design and methodology

To support a more defensible evaluation of the multicomponent kidney transplant quality improvement program a parallel two-arm cluster randomized control trial design will be used where the 26 Ontario regional renal programs (the clusters) will be randomized using covariate-constrained randomization in a 1:1 ratio to receive a multicomponent quality improvement program or the standard of care that is currently provided by the ORN. After the trial is over the other half of the renal programs will receive the multicomponent improvement program.

Administrative databases at ICES will be utilized to conduct the randomization with renal programs, which care for pre-dialysis and dialysis patients, being randomized on a single date. Each of the 26 renal programs in Ontario will be a 'cluster'. Thirteen of the 26 renal programs in Ontario will be randomized to receive the multicomponent kidney transplant quality improvement program in September 2017 (early), while the other 13 programs will receive the standard of care. In Fall 2019 the standard of care group will begin receiving the multicomponent program. The multicomponent program will have four main components: 1.

Data (e.g., provide reports to renal programs about their performance using best practices in audit and feedback); 2. Education (e.g., education toolkits for renal program providers, renal patients and families, and potential donors); 3. Peer mentorship (e.g., kidney transplant recipients and living kidney donors can mentor individuals who are going through the transplant process to provide support); and 4. Administration (e.g., helping finance nursing staff time for transplant education within each renal program). The primary outcome of interest is referral for transplantation, defined as the date the transplant centre receives a referral from the renal program. All baseline and outcome data will be retrieved from routinely collected administrative healthcare data housed at ICES.

Inclusion criteria for participant recruitment.
All of Ontario's 26 regional renal programs and their associated multidisciplinary kidney care clinics (MCKC) will be included. These programs provide care for all chronic dialysis patients. Each CKD program also provides a MCKC for pre-dialysis patients. We will be restricting to pre-dialysis patients with an eGFR <15mL/min/1.73m^2 (defined as two outpatient eGFR measurements meeting this criterion within the last year) OR a 2-year KFRE ≥25% (defined as two KFRE measurements meeting this criterion within the last year).

Considering your inclusion criteria listed above, what is the basis to exclude a potential participant?
Not applicable, we will be including all Ontario renal programs.

If using patients, describe the usual standard of care at the study site(s) for this population
Currently, there is no standard of care to improve access to kidney transplantation at the Ontario renal programs. Renal programs may provide education to their patients on treatment modalities (i.e., hemodialysis, peritoneal dialysis, transplant). The renal programs also currently provide program-level performance reports on different metrics (e.g., transplant rates); however, these are not based on best practices in audit and feedback.

Describe the study procedures and any study specific testing that will be done, outside of standard care.
The multicomponent kidney transplant quality improvement program will be at the level of the renal program, rather than the individual patient. As a result, all patients within a program will receive similar care.

How many participants over the age of 18 will be included at all study locations?
26 renal programs and their associated multidisciplinary kidney care clinics.

How many participants under the age of 18 will be included at all study locations?
No renal programs treat children exclusively; however, there could be some children transitioning to adult care.

Describe the method(s) of data analysis.
All baseline and outcome data will be ascertained from healthcare data housed at ICES. Under section 45 of Ontario's Personal Health Information Privacy Act ICES is considered a prescribed entity. Therefore, health information custodians, such as, physicians, long-term care homes, or hospitals, can disclose personal health information without consent to ICES about their patients. As a result, this evaluation can be performed without requiring the direct collection of patient identifiers or information. The statistical analyses will be conducted using an intention to treat analysis. To determine if a statistically significant difference in referral rates (primary outcome) exists between the multicomponent kidney transplant quality improvement program and the standard of care group a Poisson regression model will be used which includes potential confounders but not the intervention status. This method has been found in simulation studies to be robust even when there are a small number of clusters and when the distribution of cluster sizes is skewed. Using ICES administrative databases the renal programs' patients (dialysis and pre-dialysis) will be followed for approximately two-years censoring at death. All analyses will be conducted using the Statistical Analysis Software (SAS) version 9.4 and all analytics will be conducted at ICES Western by an ICES analyst.

How will the results of this study be made public?
Peer reviewed publication|Presentation|Other

If report to participants or other is selected above, please explain
Other can be described as providing a summary report of our findings which would include detailed statistical methodology to the Ontario Renal Network (ORN); due to word limit restrictions often imposed in peer reviewed publications we like to provide more detailed methods and resources to aid with the interpretation of results for non-researchers.

Briefly provide any plans for provision of feedback of results to the participants.
The results will be presented to patient representatives who will assist with interpreting, drafting, and developing a dissemination plan to communicate the results. Communications teams from the ORN and TGLN will be used to distribute results and publish information and resources. Results will be published in an open-access manuscript. Study findings will be disseminated through social media and news media releases.

Does this study include any use of deliberate deception or withholding of key information that may influence a participant's performance or response?
No

List any potential anticipated benefit to the participants.
The renal programs may be able to help their patients make more informed decisions around kidney transplantation by providing them with enhanced educational resources and through peer mentorship. All four parts of the multicomponent program, including data and administration, will aim to improve access to kidney transplantation at renal programs, which would benefit their patients given kidney transplantation has been found to be associated with improved survival and quality of life compared to dialysis.

List the potential benefits to society.
We hypothesize that the multicomponent kidney transplant quality improvement program will increase kidney transplant referral rates in Ontario's renal programs, thereby increasing access to kidney transplantation and increasing the number of kidney transplants in Ontario. Increasing the number of kidney transplants could result in significant financial savings to the Ontario healthcare system; kidney transplantation has significantly lower healthcare system costs compared to dialysis saving approximately $250,000 (CAD) per patient over a five-year period. These healthcare system savings could benefit all Ontario residents allowing these healthcare financial savings to be redistributed to other areas.

List any potential risks to study participants.
We are simply evaluating a program that the ORN is already planning on rolling out to their renal programs. Thus, we believe that participating in this multicomponent kidney transplant quality improvement program poses no more than a minimal risk to patients.

List any potential inconveniences to daily activities.
It is not anticipated that the multicomponent kidney transplant quality improvement program will impact renal programs' and their patients' daily activities. For example, renal programs will provide education initiatives to their patients when they were already scheduled to attend the renal program. Moreover, regarding renal program staff, part of the multicomponent program involves funding dedicated staff time to implement this strategy.

Since our unit of randomization and unit of analysis are renal programs, participants will not be approached to be recruited for this study.

Is this an industry sponsored protocol?
No

References

1. Canadian Institute for Health Information. Canadian Organ Replacement Register Annual Report: Treatment of End-Stage Organ Failure in Canada, 2000 to 2009. Ottawa, ON: CIHI; 2011.
2. Rana A, Gruessner A, Agopian VG, *et al*. Survival benefit of solid-organ transplant in the United States. *JAMA surgery*. 2015;150:252-9.
3. Ortiz F, Aronen P, Koskinen PK, *et al*. Health-related quality of life after kidney transplantation: who benefits the most? *Transpl Int*. 2014;27:1143-51.
4. Kidney Foundation of Canada. Facing the Facts. 2012. http://www.kidney.ca/document.doc?id=1376. Accessed July 7, 2015.
5. Canadian Institute for Health Information. Canadian Organ Replacement Register Annual Report: Treatment of End-Stage Organ Failure in Canada, 2003 to 2012. Ottawa, ON: CIHI; 2014.
6. Canadian Institute for Health Information. Canadian Organ Replacement Register Annual Report: Treatment of End-Stage Organ Failure in Canada, 2002 to 2011. Ottawa, ON: CIHI; 2013.
7. Barnieh L, McLaughlin K, Manns BJ, Klarenbach S, Yilmaz S, Hemmelgarn BR. Barriers to living kidney donation identified by eligible candidates with end-stage renal disease. *Nephrol Dial Transplant*. 2011;26:732-8.
8. Brehaut JC, Colquhoun HL, Eva KW, *et al*. Practice Feedback Interventions: 15 Suggestions for Optimizing Effectiveness. *Ann Intern Med.* 2016;164:435-41.
9. Waterman AD, Peipert JD, Goalby CJ, Dinkel KM, Xiao H, Lentine KL. Assessing Transplant Education Practices in Dialysis Centers: Comparing Educator Reported and Medicare Data. *Clin J Am Soc Nephrol* 2015; 10:1617-2.
10. LaPointe Rudow D, Hays R, Baliga P, *et al*. Consensus conference on best practices in live kidney donation: recommendations to optimize education, access, and care. *Am J Transplant*. 2015;15(4):914-22.
11. Skelton SL, Waterman AD, Davis LA, Peipert JD, Fish AF. Applying best practices to designing patient education for patients with end-stage renal disease pursuing kidney transplant. *Prog Transplant*. 2015;25:77-84.
12. Waterman A HS, Stanley SL, Barrett AC, Millinger R. Improving education increases dialysis patients' pursuit of transplant: Explore Transplant RCT findings. In: American Transplant Congress: May 30-June 3 2009; Boston, MA: *American journal of transplantation*; 2009: 360.
13. Hughes J, Wood E, Smith G. Exploring kidney patients' experiences of receiving individual peer support. *Health expectations* 2009;12:396-406.
14. Marlow NM, Kazley AS, Chavin KD, Simpson KN, Balliet W, Baliga PK. A patient navigator and education program for increasing potential living donors: a comparative observational study. *Clin Transplant.* 2016;30:619-27.
15. Sullivan C, Leon JB, Sayre SS, *et al*. Impact of navigators on completion of steps in the kidney transplant process: a randomized, controlled trial. *Clin J Am Soc Nephrol.* 2012;7:1639-45.

Final protocol

Reference:

Yohanna S, Naylor KL, Mucsi I, McKenzie S, Belenko D, Blake PG, Coghlan C, Dixon SN, Elliott L, Getchell L, Ki V, Nesrallah G, Patzer RE, Presseau J, Reich M, Sontrop JM, Treleaven D, Waterman AD, Zaltzman J, Garg AX. A Quality Improvement Intervention to Enhance Access to Kidney Transplantation and Living Kidney Donation (EnAKT LKD) in Patients With Chronic Kidney Disease: Clinical Research Protocol of a Cluster-Randomized Clinical Trial. *Can J Kidney Health Dis*. 2021 Apr 15;8:2054358121997266. doi: 10.1177/2054358121997266. eCollection 2021.

Table of Protocol Updates

**** All protocol updates below were made without reviewing any between-group trial outcome data (viewing and analysis will only occur after the trial period is over and the final version of the statistical analysis plan has been accepted for publication) and were done after the start of the EnAKT LKD Trial period (November 1, 2017).**

Revision	Date of Revision	Details of Revision	Rationale
Official Title	August 10, 2020	**Changed from**: A Protocol to Evaluate the Effectiveness of a Multi-component Initiative to Enhance Access to Kidney Transplantation and Living Donation: the Enhance Access to Kidney Transplantation and Living Kidney Donation Trial<br><br>**Changed to:** Quality Improvement Intervention to Enhance Access to Kidney Transplantation and Living Kidney Donation (EnAKT LKD) in Patients with Chronic Kidney Disease: A Pragmatic, Registry-based, Cluster-Randomized Clinical Trial	We changed the title to provide more details on the trial design. The change was also made to align the title with the published trial protocol.
Study Description (*Brief Summary*)	August 10, 2020	**Changed from**: Compared to dialysis, kidney transplantation is associated with improved survival, better quality of life and substantial cost savings to healthcare systems. Despite these advantages, many individuals with kidney failure never receive a kidney transplant. A multi-component quality improvement initiative was developed to enhance access to kidney transplantation and living kidney donation in Ontario's chronic kidney disease (CKD) programs. These CKD programs provide care to individuals with reduced kidney function. The initiative includes four main components: 1. Data (e.g., data collection and reports to CKD programs about their transplant related performance); 2. Education (e.g., education toolkits for CKD program staff, kidney patients and families, including living kidney donor candidates); 3. Transplant Ambassadors (e.g., kidney transplant recipients and living kidney donors who discuss transplantation and living donation to patients and their families) and 4. Administration (e.g., provincial administrative support and resources provided to CKD programs to support local work). This trial will provide high-quality evidence about the effectiveness of a multi-component quality improvement initiative aimed to enhance access to kidney transplantation and living kidney donation.<br><br>**Changed to**: Compared to dialysis, kidney transplantation is associated with improved survival, better quality of life and substantial cost savings to healthcare systems. Despite these advantages, many individuals with	The four main trial components and their delivery have not changed; we have simply refined how we describe these components. Please refer to our published trial protocol for further details on each of the intervention components.

Revision	Date of Revision	Details of Revision	Rationale
		kidney failure will never receive a kidney transplant. A multi-component quality improvement intervention (vs. usual care) provided in chronic kidney disease (CKD) programs located in Ontario, Canada was developed to determine if it can enable more patients with no recorded contraindications to kidney transplant to complete more steps towards receiving a kidney transplant. These CKD programs provide care to individuals with CKD (including patients approaching the need for dialysis and patients receiving dialysis). The intervention has four main components: (1) local quality improvement teams and administrative support; (2) tailored education and resources for staff, patients, and living kidney donor candidates; (3) support from kidney transplant recipients and living kidney donors (i.e. Transplant Ambassador Program); and (4) program-level performance reports and oversight by program leaders. The Enhance Access to Kidney Transplantation and Living Kidney Donation (EnAKT LKD) trial will provide high-quality evidence on whether a multi-component quality improvement intervention helps patients complete more steps towards receiving a kidney transplant.	
	June 23, 2022	**Added Clarification** (*Only the wording of the first component changed and the wording of all other components stayed the same)***:** (1) support for local quality improvement teams and administrative needs.	The four main trial components and their delivery have not changed; we have simply refined how we describe the first component. Please refer to our published statistical analysis plan.
Study Description (*Detailed Description*)	August 10, 2020	**Added**:<br>1. Statement of the health problem or issue:<br>Compared with dialysis, a kidney transplant offers patients a better quality of life and many gain 10 or more years of life expectancy. A transplant also costs the healthcare system less—over a five-year period. Living donor transplants offer further advantages, including superior graft and patient survival compared with deceased donor transplants. Unfortunately, many patients with kidney failure who would benefit from a transplant will never receive one. There is a chronic shortage of organs from deceased donors, and in Canada, the rate of living donor kidney transplantation has stagnated. In addition to the shortage of transplantable kidneys, several other barriers impede patient access to transplantation.	We have provided more details on the trial for people to view on ClinicalTrials.gov as per our published trial protocol.

Revision	Date of Revision	Details of Revision	Rationale
		2. Objective of your project: To determine if a quality improvement intervention provided in chronic kidney disease (CKD) programs (vs. usual care) enables more patients with no recorded contraindications to kidney transplant to complete more steps towards receiving a kidney transplant.<br>3. How will you undertake your work? We will conduct a pragmatic two-arm, parallel-group, open-label, registry-based, cluster-randomized clinical trial—the Enhance Access to Kidney Transplantation and Living Kidney Donation (EnAKT LKD) trial. Our study will include the 26 chronic kidney disease (CKD) programs in Ontario, Canada which are expected to care for over 10,000 adult patients with CKD (including patients approaching the need for dialysis and patients receiving dialysis) with no recorded contraindications to a kidney transplant during the trial. Patients in 13 of the 26 CKD programs will receive a quality improvement intervention or usual care. The intervention has four main components: (1) local quality improvement teams and administrative support; (2) tailored education and resources for staff, patients, and living kidney donor candidates; (3) support from kidney transplant recipients and living kidney donors; and (4) program-level performance reports and oversight by program leaders. Patients in the other 13 programs will receive usual care and will continue to support access to kidney transplantation and living kidney donation as usual.<br>4. What is unique/innovative about your project? An investigator usually needs to study a large number of patients in a clinical trial to reliably understand the effects of a treatment. Normally, a study with 10,000 patients would cost more than $10 million dollars to conduct; however, this study will provide a reliable answer to the question being asked and can be done at a fraction of the cost. This is because we will use data already collected by the healthcare system. The investigator will be able to analyze these healthcare data at the end of the study. This means that the study will cost less than a traditional clinical trial. This pragmatic trial includes all CKD programs in the province of	

Revision	Date of Revision	Details of Revision	Rationale
		Ontario. By including patients from a variety of backgrounds, the results of the trial should be broadly generalizable.<br>5. What is the impact of the proposed research? The EnAKT LKD trial will provide high-quality evidence on whether a multi-component quality improvement intervention helps patients complete more steps towards receiving a kidney transplant. This is important as compared to dialysis, kidney transplant offers patients a better quality of life and many gain 10 or more years of life expectancy. A transplant also costs the healthcare system less. If our intervention is successful, more transplants may ultimately be performed and result in improved survival and a better quality of life for patients with CKD. Kidney transplantation achieves the *triple aim in healthcare*: better outcomes, better experience of care, and lower costs.	
	June 23, 2022	**Added Clarification**:<br>How will you undertake your work? (*Only bolded part added):* We will conduct a pragmatic, 2-arm, parallel-group, open-label, registry-based, cluster-randomized, **superiority**, clinical trial —the Enhance Access to Kidney Transplantation and Living Kidney Donation (EnAKT LKD) trial.	No change was made to the design of the trial, rather we added the term superiority, to align with the wording in our published statistical analysis plan. This trial was always a superiority trial.
		How will you undertake your work? (*Only the wording of the first component changed and the wording of all other components stayed the same*): The intervention has four main components: (1) support for local quality improvement teams and administrative needs.	No change was made to the trial intervention, rather we updated the wording of the first component of the intervention to align with our published statistical analysis plan.
		How will you undertake your work? *(Only bolded part added):* Patients in the other 13 programs will receive usual care and will continue to **receive** support in accessing kidney transplantation and living kidney donation as usual.	No change to the trial delivery was made we just adjusted the wording to provide more clarity.
		What is unique /innovative about your project? (O*nly bolded sections changed*): An investigator usually needs to study a large number of patients in a clinical trial to reliably understand the effects of an **intervention**. Normally, a study with 10,000 patients would cost more than $10 million dollars to conduct; however, this study will provide a reliable answer to the question being asked and can be done at a fraction	No changes to the trial were made we were simply updating the terminology to align with our published statistical analysis plan. We also included an additional unique feature of our project that we thought should be emphasized; again, no change was made to the trial delivery.

Revision	Date of Revision	Details of Revision	Rationale
		of the cost. This is because we will use data **routinely** collected by the healthcare system. The investigator will be able to analyze these healthcare data at the end of the study. This means that the study will cost less than a traditional clinical trial. **Also unique to this trial, is that that the intervention was embedded and delivered in routine care.**	
Objective	August 10, 2020	**Changed from**: To determine if a multi-component kidney transplant quality improvement program increases the kidney transplant referral rate in Ontario renal programs. Specifically, we will be comparing renal program level kidney transplant referral rates between the multi-component quality improvement group and the standard-of-care group.<br>**Changed to**: To determine if a quality improvement intervention provided in chronic kidney disease (CKD) programs (vs. usual care) enables more patients with no recorded contraindications to kidney transplant to complete more steps towards receiving a kidney transplant.	We have refined our objective to incorporate our refined primary outcome (*see Primary Outcome Measures below*). The intent of this work was always to assess access to kidney transplant and with the availability of new datasets we can now do this in a refined way. Our outcome now captures the complete patient journey in access to kidney transplant.<br>We changed the term "renal program" to "chronic kidney disease program" or "CKD program" to align with the terminology used in our published trial protocol and to align with the nomenclature used by the Ontario Renal Network (part of Ontario Health) when it refers to the programs. To keep consistent with our published trial protocol we have changed the term "standard of care" to "usual care".
Study start date	March 3, 2017	**Changed from:** Half of the chronic kidney disease programs will receive the multicomponent quality improvement program early (Fall 2017).<br>**Changed to**: Provided the exact start date for the trial (November 1, 2017).	We always planned for the trial to start in Fall 2017 with the original goal of it starting in September 2017. However, after randomization we learned that the chronic kidney disease programs were not ready to start the trial until November 2017. Therefore, in our published protocol we describe the rationale for a 9-month lead time being given after randomization to give the intervention group enough time to establish their quality improvement teams and to manage schedules so team members could attend the in-person quality improvement launch event at the start of the trial period.
Study Status	February 24, 2020	**Study status revision 1:**<br>**Changed from**: *Estimated Primary Completion Da*te: November 2019<br>*Estimated Study Completion Date*: November 2019<br>**Changed to**: *Estimated Primary Completion Date*: March 31, 2021	**Study status revision 1:**<br>We increased the length of the trial to improve statistical power (*please see power section*). This will allow a better understanding of whether the multi-

Revision	Date of Revision	Details of Revision	Rationale
	 <br><br><br>December 4, 2020	*Estimated Study Completion Date*: March 31, 2021<br><br><br><br>**Study status revision 2:**<br>**Changed from**: *Estimated Primary Completion Date*: March 31, 2021 *Estimated Study Completion Date*: March 31, 2021<br>**Changed to**: *Estimated Primary Completion Date*: December 31, 2021 *Estimated Study Completion Date*: December 31, 2021	component quality improvement intervention had an impact on increasing access to kidney transplantation in Ontario.<br><br>**Study status revision 2:**<br>On March 16, 2020 nearly all kidney transplants and evaluations for deceased and living donor transplants were suspended in Ontario due to the COVID-19 pandemic. Similarly, most components of the multi-component quality improvement intervention were halted. The four components of the intervention started ramping up again in September 2020 and transplant activity started increasing in June 2020 but did not return to full capacity for several months. Extending the length of time of the multi-component quality improvement intervention in the first arm will allow adequate time for CKD programs to be exposed to the intervention when it is functioning at its full potential.
Study Follow-up Period	February 24, 2020<br><br><br>December 4, 2020<br><br><br>June 23, 2022	**Study follow-up period revision 1:**<br>**Changed from**: 2 years<br>**Changed to:** 3.4 years<br><br>**Study follow-up period revision 2:**<br>**Changed from**: 3.4 years<br>**Changed to:** 4.1 years<br><br>**Changed from**: 4.1 years<br>**Changed to**:4.17 years.	*Please see study status section above for rationale of study follow-up period revision 1 and 2.*<br><br><br><br><br>No change was made to the study follow-up period. We simply are providing a more precise time frame and this precise time frame aligns with our published statistical analysis plan.
Arm	August 10, 2020	**Changed from:** *Arm title (experimental arm)*: Multicomponent Initiative *Arm description*: A number of quality improvement initiatives will be provided at the CKD programs.<br><br>**Changed to:** *Arm title (experimental arm)*: Multi-component quality improvement intervention	No changes to the intervention were made. The change was made to make the terminology consistent with the published trial protocol.

Revision	Date of Revision	Details of Revision	Rationale
		Arm description: A multi-component quality improvement intervention will be provided at chronic kidney disease (CKD) programs.<br><br>**Change from:** *Arm title (no intervention)*: Routine Care/standard-of-care<br><br>**Changed to:** *Arm title (no intervention)*: Usual Care	No changes in the delivery of care were made to the no intervention group. We changed the terminology to "usual care" to reflect the terminology used in our published trial protocol.
Intervention	August 10, 2020<br><br><br><br><br><br><br><br><br><br><br><br><br><br><br><br><br><br><br><br>June 23, 2022	**Changed from**: *Intervention name*: Multi-component Initiative<br><br>**Changed to**: *Intervention name*: Multi-component quality improvement intervention<br><br>**Changed from:** *Intervention description*: The initiative is grounded in a quality improvement framework and has four main components for the chronic kidney disease (CKD) programs, including: 1. Data (e.g., data collection and reports to CKD programs about their performance using best practices in audit and feedback); 2. Education (e.g., education toolkits for CKD program staff, renal patients and families, including living donor candidates); 3. Transplant Ambassadors (e.g., kidney transplant recipients and living kidney donors who discuss transplantation and living donation to patients and their families) and 4. Administration (e.g., provincial administrative support and resources provided to CKD programs to support local work).<br><br>**Changed to:** *Intervention description*: The multi-component quality improvement intervention has four main components for the chronic kidney disease programs: (1) local quality improvement teams and administrative support; (2) tailored education and resources for staff, patients, and living kidney donor candidates; (3) support from kidney transplant recipients and living kidney donors; and (4) program-level performance reports and oversight by program leaders.<br><br>**Changed to:** *Intervention description (Only the wording of the first component changed and the wording of all other components stayed the same):* (1) support for local quality improvement teams and administrative needs.	The four main trial components and their delivery have not changed; we have simply refined how we describe these components. Please refer to our published trial protocol and published statistical analysis plan for further details on each of the intervention components.

Revision	Date of Revision	Details of Revision	Rationale
Primary Outcome Measures	November 19, 2019	**<u>Changed from</u>**: *Primary Outcome*: Composite outcome of living kidney donor candidate referral and transplant recipient referral event rate.<br>*Description:* The primary outcome has not been finalized. It will be finalized well before the trial ends and before the analysis of results. The outcome will be published in the peer-reviewed protocol.<br>*Time Frame*: Two years<br><br>**<u>Changed to</u>**: *Primary Outcome:* Number of key steps completed towards receiving a kidney transplant.<br>*Description*: The average number of key steps completed towards receiving a kidney transplant per 100 person-years during the trial period and analyzed at the cluster-level (chronic kidney disease program). Each step will only be counted once per patient (the first time it occurs), and each patient can contribute a maximum of four steps to their group total. The four steps include: Step I: patient referred to a transplant centre for evaluation, Step II: at least one living kidney donor candidate contacts a transplant centre for an intended recipient and completes a health history questionnaire to begin their evaluation, Step III: patient added to the deceased donor transplant wait list, and Step IV: patient receives a kidney transplant from a living or deceased donor. Patients who complete steps before the trial starts can contribute new steps during the trial period.<br>*Time Frame*: 4.1 years	Please see the "Objective" section above first for further explanation for the outcome being refined. The primary outcome was refined based on our analysis of historic records. The intent of this work is to test an intervention to improve access to kidney transplant and with the availability of new datasets this can now be done in a refined way. The four steps that comprise the primary outcome are key steps to receiving a kidney transplant. This outcome now captures the complete patient journey in access to transplant.
	June 23, 2022	**<u>Added Clarification</u>:** *Primary Outcome*: Completing key steps toward receiving a kidney transplant<br>*Description*: The primary outcome is completing key steps toward receiving a kidney transplant, where up to 4 unique steps per patient will be considered: (1) patient referred to a transplant center for evaluation, (2) a potential living kidney donor begins their evaluation at a transplant center to donate a kidney to the patient, (3) patient added to the deceased donor transplant wait list, and (4) patient receives a kidney transplant from a living or deceased donor. A patient-level analysis will be used to determine the intervention effect on completed key steps towards receiving a kidney transplant.<br>*Time Frame:* 4.17 years	No change was made to the primary outcome we simply improved the wording to make it more easily understood by the reader. Please refer to the *Analysis of Trial Outcomes* section of this table for a rational for the change from a cluster-level analysis to a patient-level analysis.

Revision	Date of Revision	Details of Revision	Rationale
Secondary Outcome Measures	June 24, 2020	**<u>Changed from</u>**:<br>1. Kidney transplantation rate (living and deceased donor kidney transplants examined separately and together)<br>*Description*: The secondary outcome has not been finalized. It will be finalized well before the trial ends and before the analysis of results. The outcome will be published in the peer-reviewed protocol.<br>*Time Frame*: Two years<br>2. Rate of pre-emptive kidney transplantation<br>*Description:* The secondary outcome has not been finalized. It will be finalized well before the trial ends and before the analysis of results. The outcome will be published in the peer-reviewed protocol.<br>*Time Frame*: Two years<br>3. Rate of kidney transplant waitlisting<br>*Description:* The secondary outcome has not been finalized. It will be finalized well before the trial ends and before the analysis of results. The outcome will be published in the peer-reviewed protocol.<br>*Time Frame*: Two years<br>4. Average Healthcare Costs<br>*Description:* The secondary outcome has not been finalized. It will be finalized well before the trial ends and before the analysis of results. The outcome will be published in the peer-reviewed protocol.<br>*Time Frame:* Two years<br>**<u>Changed to</u>**: We have pre-specified five secondary outcomes to examine the impact of our intervention on living kidney donor activity.<br>*1.* A living donor candidate contacts a transplant centre for a patient and completes a health history questionnaire to begin their evaluation <u>or</u> a patient receives a living donor transplant.<br>*Description:* Given that the average wait time for a deceased donor kidney transplant is five years on average in Ontario, our intervention is likely to have only a small impact on the rate of	The average wait time for a deceased donor kidney transplant is approximately five years in Ontario, therefore, we hypothesize that the intervention is likely to have only a small impact on the rate of deceased donor kidney transplants. For this reason, five secondary outcomes have been pre-specified to examine the impact of the intervention on the rate of living kidney donor activity.

Revision	Date of Revision	Details of Revision	Rationale
		deceased donor kidney transplants. For this reason, we have pre-specified five secondary outcomes to examine the impact of our intervention on the rate of living kidney donor transplant activity.<br>*Time Frame*: 4.1 Years<br>2. A living kidney donor candidate contacts a transplant centre for a patient and completes a health history questionnaire to begin their evaluation.<br>*Description:* Secondary outcome selected to examine the rate of living kidney donor transplant activity.<br>*Time Frame*: 4.1 Years<br>3. A transplant centre receives a patient's complete referral package from a chronic kidney disease program and a living kidney donor candidate contacts a transplant centre for a patient and completes a health history questionnaire to begin their evaluation.<br>*Description:* Secondary outcome selected to examine the rate of living kidney donor transplant activity.<br>*Time Frame*: 4.1 Years<br>4. A patient receives a living donor kidney transplant.<br>*Description*: Secondary outcome selected to examine the rate of living kidney donor transplant activity.<br>*Time Frame*: 4.1 Years<br>5. Pre-emptive living donor kidney transplants<br>*Description*: Secondary outcome selected to examine the rate of living kidney donor transplant activity. This outcome is restricted to patients who were not receiving dialysis when they entered the trial and not on dialysis at the time of transplant.<br>*Time Frame*: 4.1 Years	
	June 23, 2022	**<u>Added Clarification</u>**:<br>1. A potential living kidney donor begins their evaluation at a transplant centre to donate a kidney to the patient and/or a patient receives a living donor transplant.	All secondary outcomes were listed in our originally published protocol. No secondary outcomes where changed. We now provide additional details as we

Revision	Date of Revision	Details of Revision	Rationale
		Description: Given that the average wait time for a deceased donor kidney transplant is five years on average in Ontario, our intervention is likely to have only a small impact on the rate of deceased donor kidney transplants. For this reason, we have pre-specified five secondary outcomes to examine the impact of our intervention on living kidney donor transplant activity.<br>*Time Frame*: 4.17 Years<br>2. Time to first occurrence of a potential living kidney donor beginning their evaluation at a transplant centre to donate a kidney to the patient.<br>*Description*: Secondary outcome selected to understand living kidney donor transplant activity.<br>*Time Frame*: 4.17 Years<br>3. A transplant centre receives a patient's complete referral package from a chronic kidney disease program and at least one potential living kidney donor begins their evaluation at a transplant centre to donate a kidney to the patient.<br>*Description:* Secondary outcome selected to understand living kidney donor transplant activity.<br>*Time Frame*: 4.17 Years<br>4. A patient receives a living donor kidney transplant.<br>*Description*: Secondary outcome selected to understand living kidney donor transplant activity.<br>*Time Frame*: 4.17 Years<br>5. Pre-emptive living donor kidney transplants (restricted to patients who were not receiving dialysis when they entered the trial and not on dialysis at the time of transplant).<br>*Description*: Secondary outcome selected to understand living kidney donor transplant activity.<br>*Time Frame*: 4.17 Years	thought that the interpretation could be subjective. Please refer to our published statistical analysis plan for further details. No change was made to the time frame. We simply are providing a more precise time frame and this precise time frame aligns exactly with our published statistical analysis plan.
Other pre-specified outcomes	August 7, 2020	**Added:** We will consider several other outcomes in an exploratory analysis. Other outcome measures, include:<br>1. Rate of deceased donor kidney transplant	All other outcomes provide additional important information about access to kidney transplant. Further

Revision	Date of Revision	Details of Revision	Rationale
		Description: Rate of deceased donor kidney transplant censoring at death and receipt of a living donor kidney transplant.<br>*Time Frame*: 4.1 Years<br>2. Average number of months from the date of dialysis initiation (i.e. trial entry) to the date of referral.<br>*Description*: This outcome is assessed in patients receiving maintenance dialysis who were referred to a transplant centre.<br>*Time Frame*: 4.1 Years<br>3. Rate of living kidney donor transplants<br>*Description:* This outcome is assessed in patients waitlisted for a deceased donor kidney transplant and censored at death and receipt of a deceased donor kidney transplant.<br>Time Frame: 4.1 Years<br>4. Proportion of pre-emptive transplants<br>*Description*: This outcome is assessed in recipients of a living kidney donor transplant and restricted to patients who were not receiving dialysis when they entered the trial and not on dialysis at the time of transplant.<br>*Time Frame*: 4.1 Years<br>5. Average number of months from the date of referral to a transplant centre to the date the first living donor candidate contacts the transplant centre for the intended recipient<br>*Description*: This outcome is assessed in recipients of a living kidney donor transplant.<br>*Time Frame*: 4.1 Years<br>6. Average number of months from the date of referral to a transplant centre to date of the transplant surgery<br>*Description:* This outcome is assessed in recipients of a living or deceased donor kidney transplant.<br>*Time Frame*: 4.1 Years	details on these measures can be found in our published trial protocol.
	June 23, 2022	**Added Clarification**:<br>1. Rate of deceased donor kidney transplant	No change was made to these outcomes. We are just providing more clarification on the censoring events.

Revision	Date of Revision	Details of Revision	Rationale
		Description: Rate of deceased donor kidney transplant censoring at receipt of a living donor kidney transplant and at the censoring events included in the main trial analysis (i.e., emigration, trial end date [December 31, 2021], death, evidence of recovered kidney function, or on the date a recorded contraindication to transplant occurs [with the exception of age >75]).<br>3. Rate of living kidney donor transplants<br>*Description*: This outcome is assessed in patients waitlisted for a deceased donor kidney transplant and censored at receipt of a deceased donor kidney transplant and at the censoring events included in the main trial analysis (i.e., emigration, trial end date [December 31, 2021], death, evidence of recovered kidney function, or on the date a recorded contraindication to transplant occurs [with the exception of age >75]).<br>**Changed from**: 4.1 years<br>**Changed to**:4.17 years	No change was made to the time frame. We simply are providing a more precise time frame and this precise time frame aligns exactly with our published statistical analysis plan.
Balancing Measures	August 7, 2020	**Added:** Balancing Measures<br>• Proportion of patient referrals to a transplant centre where the referral was declined.<br>• Proportion of patient referrals to a transplant centre where the referral information was incomplete (e.g. missing diagnostics and/or other required patient information).<br>• Proportion of patient referrals to a transplant centre where the referral was deferred.<br>• Proportion of patient referrals to a transplant centre where the referral was accepted.<br>• Proportion of patients referred to a transplant centre who were not waitlisted or transplanted within 1-year of referral.<br>• Average time from patient referral to transplant centre to consulting with a transplant nephrologist (restricted to patients who had a referral that was accepted).<br>• Average time from referral to transplant centre to being waitlisted (restricted to patients who were waitlisted).	We added balancing measures to track whether our multi-component quality improvement intervention, which was designed to improve access to transplant, did not inadvertently introduce problems in other aspects of care. Further details on balancing measures are described in our published trial protocol.

Revision	Date of Revision	Details of Revision	Rationale
		• Average time from consulting with a transplant nephrologist to being waitlisted (restricted to patients who were waitlisted).<br>• Average time from referral to living kidney donor transplantation.<br>• The proportion of living donor candidates who complete all the following: a nephrology consultation, a surgeon consultation, and a computed tomography angiogram.<br>• Average time from completing the health history questionnaire to the computed tomography angiogram.<br>• Average time from completing the health history questionnaire to donor nephrectomy.<br>• Average time from nephrologist consultation to donor nephrectomy.	
Statistical Significance	December 5, 2019	**Added**: To avoid type I errors due to multiple comparisons, we will use the fixed-sequence procedure, a stepwise multiple-testing procedure where two-sided hypothesis tests for superiority will be performed at the 0.05 significance level in a pre-specified order. We will test the primary outcome first. This will be followed by the five secondary outcomes. Once a hypothesis test is not significant, no further testing will be done. Rather, the analyses of any subsequent secondary outcomes, as well as additional outcomes and other analyses will be reported as point estimates with 95% confidence intervals (without p values); we will indicate that interval widths are not adjusted for multiple testing and therefore inferences drawn may not be reproducible.	During the course of our trial newly recommended guidance was published to avoid Type 1 errors due to multiple comparisons.[1,2,3] To avoid type I errors due to multiple comparisons we will use the fixed-sequence procedure, a stepwise multiple-testing procedure.<br><br>1. Harrington D, D'Agostino RB, Gatsonis C, et al. New Guidelines for Statistical Reporting in the Journal. N Engl J Med. 2019;381(3):285-286. doi:10.1056/NEJMe1906559<br><br>2. U.S. Department of Health and Human Services, Food and Drug Administration, Center for Drug Evaluation and Research (CDER), Center for Biologics Evaluation and Research (CBER). Multiple Endpoints in Clinical Trials: Guidance for Industry. Silver Spring, MD; 2017.<br><br>3. Massachusetts Medical Society. Submitting to NEJM - Statistical Reporting Guidelines. https://www.nejm.org/author-center/new-manuscripts. Accessed December 4, 2019.
Statistical Power	August 10, 2020	**Added:** Statistical power calculations for the primary outcome (the number of steps completed towards receiving a kidney transplant) were	At the beginning of the trial the only way we could get power estimates was through conducting a literature

Revision	Date of Revision	Details of Revision	Rationale
		informed by an analysis of historical administrative healthcare data in Ontario (from November 1, 2016 to October 31, 2017). We estimate a 3.5 year trial should have at least 80% power to detect a rate ratio of 1.5 (this corresponds to patients in the intervention group completing an average of 12 more steps per 100 person-years than patients in the control group [35 steps vs. 23 steps, respectively]; 2-sided α=0.05).	review. However, a much more refined way to calculate power is to use historic transplant data that became available to us. Specifically, we could analyze historic data and get event rates which in turn informed our power calculations. See our published trial protocol for further details on our statistical power calculations.
Cohort Selection	August 10, 2020<br><br>June 23, 2022	**<u>Added:</u>** The primary analysis will be focused on individuals in multi-care kidney clinics (provide care to patients with kidney disease approaching the need for dialysis) or in dialysis programs with no recorded contraindications to kidney transplant in ICES administrative healthcare databases (i.e. patients that are eligible for transplant). Examples of recorded contraindications to transplant include dementia, use of home oxygen, living in a long-term care home, and any comorbidities likely to preclude transplantation. We will also restrict our primary analysis to individuals aged 18 to 80 as few people over age 80 are healthy enough to receive a transplant. Additional details can be found in our published trial protocol. We will also exclude patients from our analysis with invalid or missing data on date of birth or sex, and patients who are not permanent residents of Ontario (<1% will be excluded for a reason of invalid or missing data).<br><br>**<u>Changed from</u>:** Although older age is not an absolute contraindication to transplant, few people over age 80 are healthy enough to receive a transplant, and transplants in this age group are rare in Ontario; we will therefore exclude those ≥80 years of age. Additional exclusion criteria include evidence of any recorded contraindications to transplant including dementia, use of home oxygen (a sign of serious pulmonary disease), living in a long-term care home, and any comorbidities likely to preclude transplantation.<br><br>**<u>Changed to</u>:** We found that >97% of patients with one or more of the following characteristics did not receive a transplant in follow-up, and so patients with these characteristics will not enter the trial for analysis: an ESKD adapted Charlson comorbidity index score ≥7 (a higher score represents greater comorbidity), age >75 years, home oxygen use, dementia, living in a long-term care facility, receiving ≥1 physician house	Although the analysis will be restricted to individuals approaching the need for dialysis or patients on dialysis with no recorded contraindications to transplant, the delivery of the intervention is at the CKD program level and all CKD programs in Ontario are included. At the time of the final analysis, pre-specified selection criteria will be applied to restrict the statistical analysis to the patients of interest for trial inclusion.<br><br>We further refined our eligibility criteria based on an analysis we conducted where we compared 80+ baseline characteristics between patients who did and did not receive a kidney transplant during follow-up.[1] We also further refined our eligibility criteria to exclude individuals receiving conservative renal care; these individuals would not be eligible to receive a kidney transplant as they have opted for conservative care to manage their kidney disease. Conservative renal care is a new variable available in our administrative databases which we did not have access to when we wrote our original protocol.<br>**Reference:**<br>1. Wang C, Naylor KL, Luo B, *et al*. Using Administrative Health Care Databases to

Revision	Date of Revision	Details of Revision	Rationale
		call in the past year, or any of the following cancers: bladder, cervical, colorectal, liver, lung, lymphoma, or active multiple myeloma. We have also clarified that receiving conservative renal care will be considered a contraindication to transplant as these patients have decided not to purse dialysis or transplantation.	Identify Patients With End-Stage Kidney Disease With No Recorded Contraindication to Receiving a Kidney Transplant. *CJKHD* 2022 Jul 21;9:1-14.
		**Added clarification:** In our published statistical analysis plan we specified that we are using the 2021 Chronic Kidney Disease–Epidemiology Collaboration Equation, without race, to calculate the estimate glomerular filtration rate.	No change was made. We simply provided clarification on the equation that we will use to calculate the estimated glomerular filtration rate.
		**Added clarification**: In our published statistical analysis plan we included details on our cohort selection stating, “to ensure stability of kidney function, at least two eGFR or two KFRE measures were required to enter the cohort and these measures had to be separated by at least >90 days but within 365 days.”	No changes were made. We are simply providing more details.
Eligibility (*as described on ClinicalTrials.gov*)	August 10, 2020	**Changed from**: *Eligibility Criteria*: This is a pragmatic cluster randomized controlled trial with eligibility criteria detailed below.<br>**Inclusion Criteria:**<br>•All 26 chronic kidney disease (CKD) programs in Ontario. These programs provide care for all chronic dialysis patients in the province. Each CKD program also provides a multi-care kidney clinic for patients with advanced CKD who are progressing to end-stage renal disease.<br><br>**Changed to**: *Eligibility Criteria*: This is a pragmatic, two-arm, parallel-group, open-label, registry-based cluster randomized clinical trial with eligibility criteria detailed below.<br>**Inclusion Criteria:**<br>•All 26 chronic kidney disease (CKD) programs in Ontario. These programs provide care for all chronic dialysis patients in the province. Each CKD program also provides a multi-care kidney clinic for patients with advanced CKD who are approaching the need for dialysis.	No changes were made to the eligibility criteria. We have just provided more detail on the trial design. In the inclusion criteria we also refined the wording to indicate that CKD programs provide a multi-care kidney clinic for patients with advanced CKD who are approaching the need for dialysis (previously we said progressing to end-stage renal disease). The wording now aligns with our published trial protocol.
	June 23, 2022	**Added clarification**: *Eligibility Criteria (only bolded word added)*: This is a pragmatic, two-arm, parallel-group, open-label, registry-based, **superiority**, cluster randomized clinical trial with eligibility criteria detailed below.	No change was made to the design of the trial, rather we added the term superiority, to align with the wording in our published statistical analysis plan. This trial was always a superiority trial.

Revision	Date of Revision	Details of Revision	Rationale
Analysis of Trial Outcomes	August 10, 2020	**Analysis of trial outcomes revision 1:**<br>**Changed from:** To determine if a statistically significant difference in the referral rate exists between the multi-component kidney transplant quality improvement program and the standard of care group a Poisson regression model will be used which includes potential confounders but not the intervention status. This method has been found in simulation studies to be robust even when there are a small number of clusters and when the distribution of cluster sizes is skewed. Study data at ICES will be used.<br><br>**Changed to:** Study data will be obtained from Ontario's linked administrative healthcare databases at ICES (ices.on.ca). We will account for the study design and covariate-constrained randomization in our analysis. The primary outcome is at the cluster level (the rate of completing steps towards receiving a kidney transplant [per 100 person-years]) and will be compared between groups using a two-stage approach because we have 26 clusters randomized (13 per arm). In the first stage of the model, residuals are obtained from fitting a regression model to the individual level count data adjusting for pre-specified individual-level confounders while ignoring the intervention and clustering effects. In the second stage, the residuals from the first stage are aggregated at the cluster level and used as the outcome to estimate the effect of the intervention. This model fits cluster-level variables and the treatment effect.	We have refined our analysis based on feedback from our study biostatistician and a review of historic records. The new adopted statistical method was thought to be more appropriate given our updated primary outcome and number of clusters within each arm. The analysis will still be completed at the cluster level and still be done using an intent-to-treat approach. Please refer to our published trial protocol for more details.
	June 23, 2020	**Analysis of trial outcomes revision 2:**<br>**Changed from the above.**<br>**Changed to**: The primary outcome will be analyzed using a patient-level constrained multi-state model adjusting for the clustering within CKD programs. Bootstrapping at the cluster level will be used to maintain valid inference in the presence of correlated outcomes within CKD programs. We are interested in the global intervention effect for all completed steps towards transplantation. That is, we will be constraining the intervention effect to be the same for each state transition in our primary analysis. This approach will provide a single estimate of the relative rate (i.e., hazard ratio) of steps completed towards receiving a transplant among	The lead trial statistician, without knowledge of the trial results, considered different approaches as it pertained to statistical power and changed the statistical approach. For several reasons, we changed to a patient-level analysis with a multistate statistical model, which are fully described in our published statistical analysis plan.

Revision	Date of Revision	Details of Revision	Rationale
		patients in CKD programs in the intervention group versus the usual-care group.	
	August 10, 2020	**Changed from**: We will censor at death or end of study.<br>**Changed to:** We will follow patients in our analysis until the end of study, death, receipt of a kidney transplant, or become ineligible for transplant, whichever comes first.	We have refined our censoring events to ensure our denominator is capturing the population of interest.
	June 23, 2022	**Added clarification:** A patient's observation time will only stop on the trial end date (December 31, 2021), death, receipt of a kidney transplant, evidence of recovered kidney function, emigration, or on the date a recorded contraindication to transplant occurs (with the exception of age >75 years).	In our published protocol we indicated that we would stop following individuals when they become ineligible for transplant. We are also censoring at recovered kidney function as these individuals would not be actively taking steps towards a transplant.<br>We will keep individuals in the trial analysis if they turn aged > 75 years during follow-up given they were 75 years when they entered the trial, and a small proportion of older individuals receive a kidney transplant. As described in our published statistical analysis plan, we will also be censoring at emigration from the province using updated methodology available using our administrative data sources.
	August 10, 2020	**Added**: A patient's follow-up time will begin on November 1, 2017 or on the earliest date when all eligibility criteria were met up until 3 months before the trial end date (3 months is the expected minimum time to complete early steps towards receiving a kidney transplant). Patients can only enter the analytic cohort once.	We provide detail on the timeframe for entering the analytic cohort for analysis.
Additional exploratory analyses	August 10, 2020	**Added:** We have added pre-specified subgroup analyses. Specifically, in additional exploratory analyses we will consider subgroup analyses to determine if the intervention improved access to kidney transplant in the following subgroups: receiving maintenance dialysis at the time of trial entry (in-centre or home dialysis), sex (male vs. female), race (white vs. other), immigration status, geography (average distance from the patient's place of residence to the transplant centre), income quintile (measured by neighbourhood-level median income), and measures of	We want to explore the effects of the intervention in different subgroups, with a focus on subgroups that have traditionally experienced lower access to transplant.

Revision	Date of Revision	Details of Revision	Rationale
		marginalization (i.e. residential instability, material deprivation, ethnic concentration, and dependency).	
	June 23, 2022	**<u>Changed to</u>**: We will not perform subgroup analyses of the intervention effect by race (white vs. other) or immigration status.	After consultation with our project partners, we will not perform subgroup analyses by race and immigration status. We do not have access to self-reported race which is considered the gold standard for determining individuals' race and ethnicity. Ethnicity information in the Ontario Renal Reporting System was collected by data leads in each CKD program at the time of patient registration, based on charting by clinical staff who could ask patients to self-identify ethnicity but who were not mandated to do so.
		**Added**: In addition to the subgroup analyses described in our published protocol, we will also conduct subgroup analyses based on how the patient entered the trial (whether patients were approaching the need for dialysis or receiving maintenance dialysis as well as if patients entered on November 1, 2017 or during the accrual period).	We updated our subgroup analyses by including an analysis based on how the patient entered the trial. This was always an intended analysis but was not explicit in the published protocol. This information is detailed in our published statistical analysis plan.
		**Added Clarification**: As specified in our protocol, the primary analysis for this trial will not account for pandemic-related changes in transplant activity. However, we will conduct an additional analysis in which patients' follow-up times will be truncated on the date transplant activity was first suspended in Ontario as described below. We are also conducting a concurrent process evaluation using surveys and interviews to understand how the intervention was delivered in each CKD program, and we will ask respondents how the pandemic affected these activities.	Given the challenges of delivering the intervention during the COVID-19 pandemic, we will perform a pre-specified analysis of our primary and secondary outcomes restricting the trial period and follow up from November 1st, 2017 to December 20th, 2019 with follow up to March 16th, 2020. March 16th, 2020 aligns with the suspension of transplant activity in Ontario. It is possible any beneficial effect of the intervention will be more pronounced in the pre-pandemic period.
Sponsors and Collaborators	August 10, 2020	**<u>Changed from:</u>** *Collaborators*:<br>Institute for Clinical Evaluative Sciences<br>Canadian Institutes of Health Research (CIHR)<br><br>**<u>Changed to:</u>** *Collaborators*:<br>ICES (formerly known as the Institute for Clinical Evaluative Sciences)	We added the Ontario Renal Network as a collaborator. The Ontario Renal Network has always been involved with the organization and the delivery of the intervention. We are now officially listing them to align with our published trial protocol. We also now include TGLN as a collaborator.

Revision	Date of Revision	Details of Revision	Rationale
		Canadian Institutes of Health Research (CIHR)<br>Ontario Renal Network (ORN) (part of Ontario Health)<br>Trillium Gift of Life Network (TGLN) (part of Ontario Health)	

Final statistical analysis plan

Reference

Dixon SN, Naylor KL, Yohanna S, McKenzie S, Belenko D, Blake PG, Coghlan C, Cooper R, Elliott L, Getchell L, Ki V, Mucsi I, Nesrallah G, Patzer RE, Presseau J, Reich M, Sontrop JM, Treleaven D, Waterman AD, Zaltzman J, Garg AX. Enhance Access to Kidney Transplantation and Living Kidney Donation (EnAKT LKD): Statistical Analysis Plan of a Registry-Based, Cluster-Randomized Clinical Trial. *Can J Kidney Health Dis*. 2022 Nov 22;9:20543581221131201. doi: 10.1177/20543581221131201. eCollection 2022.

Final process evaluation protocol

Reference

Yohanna S, Wilson M, Naylor KL, Garg AX, Sontrop JM, Belenko D, Elliott L, McKenzie S, Macanovic S, Mucsi I, Patzer R, Voronin I, Lui I, Blake PG, Waterman AD, Treleaven D, Presseau J. Protocol for a Process Evaluation of the Quality Improvement Intervention to Enhance Access to Kidney Transplantation and Living Kidney Donation (EnAKT LKD) Cluster-Randomized Clinical Trial. *Can J Kidney Health Dis.* 2022 Mar 19;9:20543581221084502. doi: 10.1177/20543581221084502. eCollection 2022.
