# Supplementary material for: Effect of a Novel Multicomponent Intervention to Improve Patient Access to Kidney Transplant and Living Kidney Donation: The EnAKT LKD Cluster Randomized Clinical Trial
Source: JAMA Intern Med. 2023 Nov 3;183(12):1366–75. doi: 10.1001/jamainternmed.2023.5802 (PMC10696487; doi:10.1001/jamainternmed.2023.5802)
Supplement: Supplement 3. — eTable 1. Ottawa Statement eTable 2. CONSORT statement for cluster trials, pragmatic trials, and trials using routinely collected data eTable 3. Risk of bias eTable 4. CONSERVE-CONSORT (CONSORT Extension for RCTs Revised in Extenuating Circumstance) eTable 5. Missing baseline data eTable 6. Outcome ascertainment, data cleaning, and handling of missing data for the steps completed toward receiving a kidney transplant eTable 7. Intracluster correlation coefficient (ICC) and coefficient of variation measures for the outcomes eTable 8. Multistate model assumptions eTable 9. Patient follow-up eTable 10. Primary outcome examined in two multistate models: the first unadjusted for baseline characteristics and the second adjusted for additional baseline characteristics. eTable 11. Effect of the intervention on each transition (shown visually in eFigure 1a and 1b); these estimates are from an unconstrained model eTable 12. Effect of the intervention on steps not specified as primary or secondary outcomes eTable 13. Effect of the intervention when follow-up was truncated to March 16, 2020, the start of the COVID-19 pandemic in Ontario eTable 14. Effect of the intervention on the primary composite outcome in multiple subgroups eTable 15. The time to complete steps and other measures in the intervention group and usual-care group eTable 16. Effect of the intervention when restricted to patients who completed no steps toward receiving a transplant before trial entry eTable 17. Effect of the intervention when restricted to patients who entered the trial approaching the need for dialysis eTable 18. Effect of the intervention when restricted to patients who were receiving maintenance dialysis when they entered the trial eTable 19. Rates of kidney transplantation (total, living, deceased) in intervention and usual-care groups in pre-trial, trial, and pre-COVID-19 pandemic periods eFigure 1. Multistate models for the intervention and usual-care groups eFigure 2. Forest plot of the eff [file jamainternmed-e235802-s003.pdf]

# Supplemental Online Content

Garg AX, Yohanna S, Naylor KL, et al. Effect of a novel multicomponent intervention to improve patient access to kidney transplant and living kidney donation: the EnAKT LKD cluster randomized clinical trial. *JAMA Intern Med.* Published online November 3, 2023. doi:10.1001/jamainternmed.2023.5802

**eTable 1.** Ottawa Statement

**eTable 2.** CONSORT statement for cluster trials, pragmatic trials, and trials using routinely collected data

**eTable 3.** Risk of bias

**eTable 4.** CONSERVE-CONSORT (CONSORT Extension for RCTs Revised in Extenuating Circumstance)

**eTable 5.** Missing baseline data

**eTable 6.** Outcome ascertainment, data cleaning, and handling of missing data for the steps completed toward receiving a kidney transplant

**eTable 7.** Intracluster correlation coefficient (ICC) and coefficient of variation measures for the outcomes

**eTable 8.** Multistate model assumptions

**eTable 9.** Patient follow-up

**eTable 10.** Primary outcome examined in two multistate models: the first unadjusted for baseline characteristics and the second adjusted for additional baseline characteristics.

**eTable 11.** Effect of the intervention on each transition (shown visually in **eFigure 1** a and 1b); these estimates are from an unconstrained model

**eTable 12.** Effect of the intervention on steps not specified as primary or secondary outcomes

**eTable 13.** Effect of the intervention when follow-up was truncated to March 16, 2020, the start of the COVID-19 pandemic in Ontario

**eTable 14.** Effect of the intervention on the primary composite outcome in multiple subgroups

**eTable 15.** The time to complete steps and other measures in the intervention group and usual-care group

**eTable 16.** Effect of the intervention when restricted to patients who completed no steps toward receiving a transplant before trial entry

© 2023 Garg AX et al. *JAMA Internal Medicine.*

**eTable 17.** Effect of the intervention when restricted to patients who entered the trial approaching the need for dialysis

**eTable 18.** Effect of the intervention when restricted to patients who were receiving maintenance dialysis when they entered the trial

**eTable 19.** Rates of kidney transplantation (total, living, deceased) in intervention and usual-care groups in pre-trial, trial, and pre-COVID-19 pandemic periods

**eFigure 1.** Multistate models for the intervention and usual-care groups

**eFigure 2.** Forest plot of the effect of the intervention on the primary composite outcome in multiple subgroups

This supplemental material has been provided by the authors to give readers additional information about their work.

© 2023 Garg AX et al. *JAMA Internal Medicine.*

**Trial Team**

1.1 Steering Committee and Team

*Steering Committee*: Amit X. Garg, Seychelle Yohanna, Kyla L. Naylor, Susan McKenzie, Istvan Musci, Stephanie N. Dixon, Bin Luo, Jessica M. Sontrop, Mary Beaucage, Dmitri Belenko, Candice Coghlan, Rebecca Cooper, Lori Elliot, Leah Getchell, Esti Heale, Vincent Ki, Gihad Nesrallah, Rachel E. Patzer, Justin Presseau, Marian Reich, Darin Treleaven, Carol Wang, Amy D. Waterman, Jeffrey Zaltzman, Peter G. Blake

*Team*: Staff and leaders at all participating chronic kidney disease programs. Staff at the Ontario Renal Network and Trillium Gift of Life Network (both part of Ontario Health).

1.2 Patient and Family Partners

Susan Q. McKenzie (Lead), Mary Beaucage, Candice Coghlan, Leah Getchell, Marian Reich

1.3 Transplant Education Group

Istvan Musci (Lead), Dmitri Belenko, Amy Waterman

1.4 Statistics Group

Stephanie N. Dixon (Lead), Bin Luo, Kyla L. Naylor, Amit X. Garg

1.5 Participating Chronic Kidney Disease Programs (26 programs in alphabetical order) *

| | |
|---|---|
| Grand River Hospital | Sault Area Hospital |
| Halton Healthcare | Scarborough and Rouge Hospital |
| Health Sciences North | St. Joseph's Health Centre Toronto |
| Humber River Hospital | St. Joseph's Healthcare Hamilton |
| Kingston Health Sciences Centre | St. Michael's Hospital |
| Lakeridge Health | Sunnybrook Health Sciences Centre |
| London Health Sciences Centre | The Ottawa Hospital |
| Mackenzie Health | Thunder Bay Regional Health Sciences Centre |
| Niagara Health | Timmins and District Hospital |
| North Bay Regional Health Centre | Trillium Health Partners |
| Orillia Soldiers' Memorial Hospital | University Health Network |
| Peterborough Regional Health Centre | William Osler Health System |
| Renfrew Victoria Hospital | Windsor Regional Hospital |

*All Chronic Kidney Disease (CKD) programs in Ontario were randomized to the intervention or usual care. On April 1, 2018, Orillia Soldiers' Memorial Hospital split into two CKD Programs, creating 27 CKD programs. We treated the new CKD program (Royal Victoria Regional Health Centre) as part of Orillia Soldiers' Memorial Hospital.

© 2023 Garg AX et al. *JAMA Internal Medicine.*

## eTables 1 to 4. Adherence to trial reporting guidelines and risk of bias assessment

To assess trial compliance, we used the following reporting guidelines[a]: **1)** Ottawa Statement, **2)** CONSORT statement for cluster-randomization trials, pragmatic trials, and trials using routinely collected data, **3)** the risk of bias using the Revised Cochrane risk of bias tool for randomized trials (RoB 2) Additional considerations for cluster-randomized trials and **4)** CONSERVE statement for reporting trials modified due to the COVID-19 Pandemic and other extenuating circumstances.

We used information from several documents to complete the assessments, including Manuscript, Manuscript Supplementary Appendix, Published Study Protocol[b], Published Statistical Analysis Plan[c], Published Study Process Evaluation Protocol[d], and Protocol Compilation (Table of Protocol Updates- available on ClinicalTrials.gov).

[a]The Template for Intervention Description and Replication Criteria (TIDieR) checklist will be reported with the EnAKT LKD process evaluation manuscript.

[b]Yohanna S, Naylor KL, Mucsi I, et al. A quality improvement intervention to enhance access to kidney transplantation and living kidney donation (EnAKT LKD) in patients with chronic kidney disease: clinical research protocol of a cluster-randomized clinical trial. *Can J Kidney Health Dis*. 2021;8:2054358121997266. doi:10.1177/2054358121997266.

[c]Dixon SN, Naylor KL, Yohanna S, McKenzie S, Belenko D, Blake PG, et al. Enhance Access to Kidney Transplantation and Living Kidney Donation (EnAKT LKD): Statistical Analysis Plan of a Registry-Based, Cluster-Randomized Clinical Trial. *Can J Kidney Health Dis*. 2022; 9:20543581221131201. doi: 10.1177/20543581221131201

[d]Yohanna S, Wilson M, Naylor KL, et al. Protocol for a Process Evaluation of the Quality Improvement Intervention to Enhance Access to Kidney Transplantation and Living Kidney Donation (EnAKT LKD) Cluster-Randomized Clinical Trial. *Can J Kidney Health Dis.* 2022;9:20543581221084502. doi: 10.1177/2054358122108450

## eTable 1. Ottawa Statement[a]

| Ethical Issue | Recommendation | Location in manuscript |
|---|---|---|
| Justifying the cluster randomized design | Researchers should provide a clear rationale for the use of the cluster randomized design and adopt statistical methods appropriate for this design. | Manuscript, section- Methods (Study Design)<br>Published Study Protocol- Methods (Study Design and Setting)<br>Published Statistical Analysis Plan- Study Methods (Analysis Methods) |
| Research ethics committee review | Researchers must submit a cluster randomized trial involving human research participants for approval by a research ethics committee before commencing. | Manuscript, section- Methods (Study Design)<br>Published Study Protocol-Methods (Ethical Considerations) |
| Identifying research participants | Researchers should clearly identify the research participants in cluster randomized trials. A research participant can be identified as an individual whose interests may be affected as a result of study interventions or data collection procedures, that is, an individual (1) who is the intended recipient of an experimental (or control) intervention; or (2) who is the direct target of an experimental (or control) manipulation of his/her environment; or (3) with whom an investigator interacts for the purpose of collecting data about that | Manuscript, section- Methods (Study Design) (Study Setting and Participants); Figure<br>Published Study Protocol- Methods (Inclusion Criteria) (Statistical Analysis- Trial Population) (Statistical Analysis-Eligibility criteria)<br>Published Statistical Analysis Plan- Study Methods (Trial Design), Table 1 |

© 2023 Garg AX et al. *JAMA Internal Medicine.*

| | | |
|---|---|---|
| | individual; or (4) about whom an investigator obtains identifiable private information for the purpose of collecting data about that individual. Unless one or more of these criteria is met, an individual is not a research participant. | |
| Obtaining informed consent | Researchers must obtain informed consent from human research participants in a cluster randomized trial, unless a waiver of consent is granted by a research ethics committee under specific circumstances. | Waived consent granted<br>Manuscript, section: Methods (Study Design)<br>Published Study Protocol: Methods (Ethical Considerations); Supplemental Appendix 13 |
| | When participants' informed consent is required, but recruitment of participants is not possible before randomization of clusters, researchers must seek participants' consent for trial enrolment as soon as possible after cluster randomization—that is, as soon as the potential participant has been identified, but before the participant has undergone any study interventions or data collection procedures. | Not applicable |
| | A research ethics committee may approve a waiver or alteration of consent requirements when (1) the research is not feasible without a waiver or alteration of consent, and (2) the study interventions and data collection procedures pose no more than minimal risk. | Published Study Protocol: Methods (Ethical Considerations); Supplemental Appendix 13 |
| | Researchers must obtain informed consent from professionals or other service providers who are research participants unless conditions for a waiver or alteration of consent are met. | Not applicable<br>Manuscript, section: Methods (Study Setting and Participants)<br>Published Study Protocol: Methods (Ethical Considerations) |
| Gatekeepers | Gatekeepers should not provide proxy consent on behalf of individuals in their cluster. | Manuscript, section- Methods (Study Setting and Participants)<br>Published Study Protocol- Discussion<br>This item was given careful consideration and our approach was approved by our Research Ethics Board. |
| | When a cluster randomized trial may substantially affect cluster or organizational interests, and a gatekeeper possesses the legitimate authority to make decisions on the cluster or organization's behalf, the researcher should obtain the gatekeeper's permission to enroll the cluster or organization in the trial. Such permission does not replace the need for the informed consent of research participants. | Manuscript, section- Methods (Study Setting and Participants)<br>Published Study Protocol- Methods (Ethical Considerations); Supplemental Appendix 13 |
| | When cluster randomized trial interventions may substantially affect cluster interests, researchers should seek to protect cluster interests through cluster consultation to inform study design, conduct, and | Published Study Protocol: Background (Development of the Quality Improvement Intervention and Its Components) |

© 2023 Garg AX et al. *JAMA Internal Medicine.*

| | | |
|---|---|---|
| | reporting. Where relevant, gatekeepers can often facilitate such a consultation. | |
| Assessing benefits and harms | The researcher must ensure that the study intervention is adequately justified. The benefits and harms of the study intervention must be consistent with competent practice in the field of study relevant to the cluster randomized trial. | Manuscript, section: Introduction<br>Published Study Protocol: Methods (Data Monitoring, Harms, and Auditing)<br>Published Statistical Analysis Plan: Analysis (Harms and Data Monitoring) |
| | Researchers must adequately justify the choice of the control condition. When the control arm is usual practice or no treatment, individuals in the control arm must not be deprived of effective care or programs to which they would have access, were there no trial. | Published Study Protocol: Methods (Usual care) |
| | Researchers must ensure that data collection procedures are adequately justified. The risks of data collection procedures must (1) be minimized consistent with sound design and (2) stand in reasonable relation to the knowledge to be gained. | Manuscript, section- Methods (Study Design) (Study Setting and Participants)<br>Published Study Protocol: Methods (Data Collection and Data Sources)<br>Published Study Process Evaluation Protocol: Background (The Need for a Process Evaluation of the EnAKT LKD Trial), Methods (Participants) (Data Collection) |
| Protecting vulnerable participants | Clusters may contain vulnerable participants. In these circumstances, researchers and research ethics committees must consider whether additional protections are needed. | The Research Ethics Board considered vulnerable patients with advanced chronic kidney disease or patients receiving maintenance dialysis in this cluster trial. The Research Ethics Board agreed that the trial met the criteria for a waiver of patient consent for participation.<br>Manuscript, section: Methods (Study Design)<br>Published Study Protocol: Methods (Ethical Considerations), Supplemental Appendix 13 |
| | When individual informed consent is required and there are individuals who may be less able to choose participation freely because of their position in a cluster or organizational hierarchy, research ethics committees should pay special attention to recruitment, privacy, and consent procedures for those participants. | Not applicable.<br>Published Study Protocol- Methods (Ethical Considerations); Supplemental Appendix 13 |

[a]Weijer C, Grimshaw JM, Eccles MP, et al. The Ottawa Statement on the Ethical Design and Conduct of Cluster Randomized Trials. *PLoS Med*. 2012;9(11):e1001346.

© 2023 Garg AX et al. *JAMA Internal Medicine.*

**eTable 2**. CONSORT statement for cluster-randomized trials, pragmatic trials and trials using routinely collected data[a,b,c,d]

| Section/Topic | Standard CONSORT description[a] | Pragmatic trials[b] | Cluster design[c] | Routinely collected data[d] | Location in manuscript |
|---|---|---|---|---|---|
| **TITLE** | Identification as a randomised trial in the title | | Identification as a cluster randomised trial in the title | | Manuscript, section: Title |
| **ABSTRACT** | Structured summary of trial design, methods, results, and conclusions (for specific guidance see CONSORT for abstracts). | | Identification as a cluster-randomised trial in the abstract | Specify that a cohort or routinely collected data were used to conduct the trial and, if applicable, provide the name of the cohort or routinely collected database(s) | Manuscript, section: Abstract<br>Manuscript, section: Methods (Study Design)<br>Limited details could only be provided in the Abstract due to word limit restrictions. Therefore, some of this information is contained in other Manuscript sections (Methods [Study Design]). |
| **INTRODUCTION** | | | | | |
| **Background** | Scientific background and explanation of rationale | Describe the health or health service problem that the intervention is intended to address and other interventions that may commonly be aimed at this problem | Rationale for using a cluster design | | Manuscript, section: Introduction<br>Manuscript, section: Methods (Study Design)<br>Published Study Protocol: (Background and Discussion) |
| | Specific objectives or hypotheses | | Whether objectives pertain to the cluster level, the individual participant level or both | | Manuscript, section: Introduction<br>Manuscript, section: Methods (Study Design)<br>Published Study Statistical Analysis Plan: Study Methods (Trial Objectives and Hypotheses) |
| **METHODS** | | | | | |
| **Trial design** | Description of trial design (such as parallel, factorial) including allocation ratio | | Definition of cluster and description of how the design features apply to the clusters | Description of the cohort or routinely collected database(s) used to conduct the trial (such as electronic health record, registry) and how the data were used within the trial (such as | Manuscript, section: Methods (Study Design) (Study Setting and Participants)<br>Published Study Protocol-Methods (Ethics, Randomization, Loss to Follow-Up, Data Collection and Data Sources, Statistical Power, |

© 2023 Garg AX et al. *JAMA Internal Medicine.*

| Section/Topic | Standard CONSORT description[a] | Pragmatic trials[b] | Cluster design[c] | Routinely collected data[d] | Location in manuscript |
|---|---|---|---|---|---|
| | | | | identification of eligible trial participants, trial outcomes) | Statistical Analysis, Supplemental Appendix 14)<br><br>Published Study Statistical Analysis Plan-Study Methods (Trial Design, Timing of Outcome Assessment and Analysis, Table 1) |
| | Important changes to methods after trial commencement (such as eligibility criteria), with reasons | | | | Protocol compilation- Table of Protocol Updates<br><br>Published Study Statistical Analysis Plan- Study Methods (Adherence and Protocol Deviations); Analysis (Changes to the analysis of the primary outcome from the published protocol; Subgroup analyses); Appendix 4 |
| | | | | Name, if applicable, and description of the cohort or routinely collected database(s) used to conduct the trial, including information on the setting (such as primary care), locations, and dates, (such as periods of recruitment, follow-up, and data collection) | Manuscript, section: Methods (Study Design, Study Setting and Participants)<br><br>Published Study Protocol-Appendix 14 |
| | | | | Eligibility criteria for participants in the cohort or routinely collected database(s) | Manuscript, section: Methods (Study Setting and Participants)<br><br>Published Study Statistical Analysis Plan: Table 1 and Trial Population (Eligibility Criteria) |
| | | | | State whether the study included person-level, institutional-level, or other data linkage across two or more databases and, if so, linkage techniques and methods used to evaluate | Manuscript, section: Methods (Study Setting and Participants); Supplement eTables<br><br>Published Study Protocol: Methods (Data Collection and Data Sources); Supplemental Appendix 14 |

© 2023 Garg AX et al. *JAMA Internal Medicine.*

| Section/Topic | Standard CONSORT description[a] | Pragmatic trials[b] | Cluster design[c] | Routinely collected data[d] | Location in manuscript |
|---|---|---|---|---|---|
| | | | | completeness and accuracy of linkage | |
| **Participants** | Eligibility criteria for participants | Eligibility criteria should be explicitly framed to show the degree to which they include typical participants and/or, where applicable, typical providers (eg, nurses), institutions (eg, hospitals), communities (or localities eg, towns) and settings of care (eg, different healthcare financing systems) | Eligibility criteria for clusters | Eligibility criteria for trial participants, including information on how to access the list of codes and algorithms used to identify eligible participants, information on accuracy and completeness of data used to ascertain eligibility, and methods used to validate accuracy and completeness (e.g., monitoring, adjudication), if applicable | Manuscript, section: Methods (Study Setting and Participants); Data Sharing Statement<br><br>Published Study Protocol: Methods (Inclusion Criteria), Statistical Analysis (Eligibility Criteria)<br><br>Published Statistical Analysis Plan: Table 1<br><br>Additional detail on capturing patients with no recorded contraindication to kidney transplant was previously published in "Wang et al. Using administrative healthcare databases to identify patients with end stage kidney disease with no recorded contraindication to receiving a kidney transplant. *Can J Kidney Heal Dis*. 2022;9." |
| | Settings and locations where the data were collected | | | | Manuscript, section: Methods (Study Setting and Participants)<br><br>Published Study Protocol: Methods (Inclusion Criteria) |
| | Describe whether and how consent was obtained | | | | Manuscript, section: Methods (Study Design)<br><br>Published Study Protocol: Methods (Ethics); Supplemental Appendix 13 |
| **Interventions** | The interventions for each group with sufficient details to allow replication, including how and when they were actually administered | Describe extra resources added to (or resources removed from) usual settings in order to implement intervention. Indicate if efforts were made to standardise the intervention or if the | Whether interventions pertain to the cluster level, the individual participant level or both | | Manuscript, section: Methods (Study Design)<br><br>Published Study Protocol: Methods (Intervention) (Characterizing the Trial Within the Pragmatic-Explanatory Continuum) |

© 2023 Garg AX et al. *JAMA Internal Medicine.*

| Section/Topic | Standard CONSORT description[a] | Pragmatic trials[b] | Cluster design[c] | Routinely collected data[d] | Location in manuscript |
|---|---|---|---|---|---|
| | | intervention and its delivery were allowed to vary between participants, practitioners, or study sites | | | Published Study Process Evaluation Protocol: Background (The EnAKT LKD Cluster-Randomized Clinical Trial) and Supplemental Appendix 1 |
| | | Describe the comparator in similar detail to the intervention | | | Manuscript, section: Methods (Intervention)<br>Published Study Protocol: Methods (Usual Care)<br>Published Study Process Evaluation Protocol: Supplemental Appendix 1 |
| **Outcomes** | Completely defined prespecified primary and secondary outcome measures, including how and when they were assessed | Explain why the chosen outcomes and, when relevant, the length of follow-up are considered important to those who will use the results of the trial | Whether outcome measures pertain to the cluster level, the individual participant level or both | Information on how to access the list of codes and algorithms used to define or derive the outcomes from the cohort or routinely collected database(s) used to conduct the trial, information on accuracy and completeness of outcome variables, and methods used to validate accuracy and completeness (e.g., monitoring, adjudication), if applicable | Manuscript, section: Methods (Outcomes), (Statistical Analysis)<br>Manuscript, section: Supplementary Appendix eFigures<br>Manuscript, section: Data Sharing Statement<br>Manuscript section: Supplementary Appendix, eTables<br>Published Study Protocol: Methods (Primary Outcome) (Secondary Outcomes)<br>Protocol Compilation: Table of Protocol Updates (Objective), (Primary Outcome Measures), (Secondary Outcome Measures) |
| | Any changes to trial outcomes after the trial commenced, with reasons | | | | Protocol Compilation: Table of Protocol Updates (Objective), (Primary Outcome Measures), (Secondary Outcome Measures) |
| **Sample size** | How sample size was determined | If calculated using the smallest difference considered important by the target decision maker audience (the minimally important difference) then | Method of calculation, number of clusters(s) (and whether equal or unequal cluster sizes are assumed), cluster size, a coefficient of intracluster correlation (ICC or k), and an | | Manuscript, section: Methods (Sample Size Calculation)<br>Published Study Protocol: Methods (Statistical Power)<br>Published Statistical Analysis Plan: Study Methods (Sample Size) |

© 2023 Garg AX et al. *JAMA Internal Medicine.*

| Section/Topic | Standard CONSORT description[a] | Pragmatic trials[b] | Cluster design[c] | Routinely collected data[d] | Location in manuscript |
|---|---|---|---|---|---|
| | | report where this difference was obtained | indication of its uncertainty | | Supplement eTable |
| | When applicable, explanation of any interim analyses and stopping guidelines | | | | Not applicable, see:<br>-Published Study Protocol: Methods (Analysis of trial outcomes)<br>-Published Statistical Analysis Plan: Methods (Interim Analysis) |
| **Randomisation—sequence generation** | Method used to generate the random allocation sequence, including details of any restriction (e.g., blocking, stratification) | | Details of stratification or matching if used | | Manuscript, section: Methods (Randomization)<br><br>Published Study Protocol: Methods (Randomization) |
| **Randomisation—allocation concealment** | Mechanism used to implement the random allocation sequence (such as sequentially numbered containers), describing any steps taken to conceal the sequence until interventions were assigned | | Specification that allocation was based on clusters rather than individuals and whether allocation concealment (if any) was at the cluster level, the individual participant level or both | Mechanism used to implement the random allocation sequence (such as embedding an automated randomiser within the cohort or routinely collected database(s)). | Manuscript, section: Methods (Randomization)<br>Published Study Protocol: Methods (Randomization) |
| **Randomisation—implementation** | Who generated the allocation sequence, who enrolled participants, and who assigned participants to their groups | | Who generated the random allocation sequence, who enrolled clusters, and who assigned clusters to interventions | | Manuscript, section: Methods (Randomization)<br>Published Study Protocol: Methods (Randomization) |
| | | | Mechanism by which individual participants were included in clusters | | Manuscript, section: Methods (Study Setting and Participants) (Randomization) |

© 2023 Garg AX et al. *JAMA Internal Medicine.*

| Section/Topic | Standard CONSORT description[a] | Pragmatic trials[b] | Cluster design[c] | Routinely collected data[d] | Location in manuscript |
|---|---|---|---|---|---|
| | | | for the purposes of the trial (such as complete enumeration, random sampling) | | Published Study Protocol: Methods (Inclusion Criteria) (Randomization) |
| | | | From whom consent was sought (representatives of the cluster, or individual cluster members, or both), and whether consent was sought before or after randomisation | | Manuscript, section: Methods (Study Design) (Study Setting and Participants)<br><br>Published Study Protocol: Methods (Ethics) |
| **Blinding (masking)** | If done, who was blinded after assignment to interventions (for example, participants, care providers, those assessing outcomes) and how | If blinding was not done, or was not possible, explain why | | | Not applicable, see:<br>Published Study Protocol: Methods (Blinding) |
| **Statistical methods** | Statistical methods used to compare groups for primary outcomes; methods for additional analyses, such as subgroup analyses and adjusted analyses | | How clustering was taken into account | | Manuscript, section: Methods (Statistical Analysis)<br>Published Statistical Analysis Plan: Methods (Analysis Methods) (Analysis of the Secondary Outcomes) (Additional Analyses) (Subgroup analyses) (Restricting the trial to the Pre-COVID period) |
| **RESULTS** | | | | | |
| **Participant flow** | For each group, the numbers of participants who were randomly assigned, received intended treatment, and were analysed for the primary outcome | The number of participants or units approached to take part in the trial, the number which were eligible, and reasons for non-participation should be reported | For each group, the numbers of clusters that were randomly assigned, received intended treatment, and were analysed for the primary outcome | For each group, the number of participants in the cohort or routinely collected database(s) used to conduct the trial and the numbers screened for eligibility, randomly assigned, offered, and accepted interventions (e.g., cohort multiple RCTs), | Manuscript, section: Figure |

© 2023 Garg AX et al. *JAMA Internal Medicine.*

| Section/Topic | Standard CONSORT description[a] | Pragmatic trials[b] | Cluster design[c] | Routinely collected data[d] | Location in manuscript |
|---|---|---|---|---|---|
| | | | | received intended treatment, and analysed for the primary outcome | |
| | For each group, losses and exclusions after randomisation, together with reasons | | For each group, losses and exclusions for both clusters and individual cluster members | | Manuscript, section: Figure, Supplement eTables |
| **Recruitment** | Dates defining the periods of recruitment and follow-up | | | | Manuscript section: Methods (Study Setting and Participants); Results |
| | Why the trial ended or was stopped | | | | Not applicable |
| **Baseline data** | A table showing baseline demographic and clinical characteristics for each group | | Baseline characteristics for the individual and cluster levels as applicable for each group | | Manuscript, section: Table 1 |
| **Numbers analysed** | For each group, number of participants (denominator) included in each analysis and whether the analysis was by original assigned groups | | For each group, number of clusters included in each analysis | | Manuscript, section: Results, Figure , Table 2<br>Manuscript, section: Supplement eTables |
| **Outcomes and estimation** | For each primary and secondary outcome, a summary of results for each group and the estimated effect size and its | | Results at the individual or cluster level as applicable and a coefficient of intracluster correlation (ICC or k) for each primary outcome | | Manuscript section: Results, Table 2<br>Supplement eTables |

© 2023 Garg AX et al. *JAMA Internal Medicine.*

| Section/Topic | Standard CONSORT description[a] | Pragmatic trials[b] | Cluster design[c] | Routinely collected data[d] | Location in manuscript |
|---|---|---|---|---|---|
| | precision (e.g., 95% CI) | | | | |
| | For binary outcomes, presentation of both absolute and relative effect sizes is recommended | | | | Manuscript section: Table 2 |
| **Ancillary analyses** | Address multiplicity by reporting any other analyses performed, including subgroup analyses and adjusted analyses, indicating which are prespecified and which are exploratory | | | | Manuscript section: Methods (Statistical Analysis); Results (Additional Analyses)<br>Manuscript, section: Supplement eTables |
| **Harms** | All important harms or unintended effects in each group (for specific guidance see CONSORT for harms) | | | | Manuscript, section: Supplement eTables<br>Published Study Protocol Methods (Balancing Measures) (Data Monitoring, Harms, and Auditing) |
| **DISCUSSION** | | | | | |
| **Limitations** | Trial limitations, addressing sources of potential bias, imprecision, and, if relevant, multiplicity of analyses | | | | Manuscript, section: Discussion |
| **Interpretation** | Interpretation consistent with results, balancing benefits and harms, and considering | | | Implications of using data that were not collected to answer the trial research questions | Manuscript, section: Discussion |

© 2023 Garg AX et al. *JAMA Internal Medicine.*

| Section/Topic | Standard CONSORT description[a] | Pragmatic trials[b] | Cluster design[c] | Routinely collected data[d] | Location in manuscript |
|---|---|---|---|---|---|
| | other relevant evidence | | | | |
| **Generalisability** | Generalisability (external validity, applicability) of the trial findings | Describe key aspects of the setting which determined the trial results. Discuss possible differences in other settings where clinical traditions, health service organisation, staffing, or resources may vary from those of the trial | Generalisability to clusters and/or individual participants (as relevant) | | Manuscript, section: Discussion<br>Published Study Protocol, section: Discussion |
| **Overall evidence** | General interpretation of the results in the context of current evidence | | | | Manuscript, section: Discussion |
| **OTHER INFORMATION** | | | | | |
| **Registration** | Registration number and name of trial registry | | | | Manuscript, section: Abstract |
| **Protocol** | Where the full trial protocol can be accessed, if available | | | | Published Study Protocol (Supplement 1)<br>Published study process evaluation<br>Published study statistical analysis plan (Supplement 2)<br>Protocol Compilation |
| **Funding** | Sources of funding and other support (such as supply of drugs), role of funders | | | Sources of funding for the cohort or routinely collected database(s), role of funders | Manuscript, section: Funding/Support, Role of the Funder / Sponsor, Acknowledgements |

[a] Schulz KF et al. CONSORT 2010 Statement: Updated guidelines for reporting parallel group randomised trials. BMJ. 2010;340:c332
[b] Zwarenstein M et al. Improving the reporting of pragmatic trials: An extension of the CONSORT statement. BMJ. 2008;337:a2390.
[C] Campbell MK et al. Consort 2010 statement: extension to cluster randomised trials. *BMJ*. 2012;345(7881). doi:10.1136/BMJ.E5661
[d] Kwakkenbos L et al. CONSORT extension for the reporting of randomised controlled trials conducted using cohorts and routinely collected data. BMJ 2021;373:n857.

© 2023 Garg AX et al. *JAMA Internal Medicine.*

**eTable 3. Risk of bias[a]**

| Intention-to-treat | Unique ID | Study ID | Experimental | Comparator | Outcome |
|---|---|---|---|---|---|
| | EnAKT LKD | 1 | Multicomponent intervention | Usual care | Steps completed toward receiving a kidney transplant |

| Legend | | Domains |
|---|---|---|
| + | Low Risk | D1a Randomisation process |
| ! | Some Concerns | D1b Timing of identification or recruitment of participants |
| - | High risk | D2 Deviations from the intended interventions |
| | | D3 Missing outcome data |
| | | D4 Measurement of the outcome |
| | | D5 Selection of the reported result |

| D1a | D1b | D2 | D3 | D4 | D5 | Overall |
|---|---|---|---|---|---|---|
| + | + | + | + | + | + | + |

| Domain | Signalling question | Response | Comments |
|---|---|---|---|
| Bias arising from the randomization process | 1a.1 Was the allocation sequence random? | Yes | The CKD programs were randomly allocated (1:1) to the intervention arm or the usual-care arm using covariate-constrained randomization, stratified by historic transplant center referral patterns. The allocation scheme was computer- generated and was concealed from CKD programs and most members of the trial team. Covariate-constrained randomization was used to ensure key baseline characteristics were balanced between arms. |
| | 1a.2 Was the allocation sequence concealed until clusters were enrolled and assigned to interventions? | Yes | |
| | 1a.3 Did baseline differences between intervention groups suggest a problem with the randomization process? | No | |
| | **Risk of bias judgement** | **Low** | |

© 2023 Garg AX et al. *JAMA Internal Medicine.*

| | | | |
|---|---|---|---|
| Bias arising from the timing of identification or recruitment of participants | 1b.1 Were all the individual participants identified and recruited (if appropriate) before randomization of clusters? | Partial No | Both prevalent and incident patients were included in the trial; some patients entered the trial at the beginning of the trial period and some entered during the trial period. |
| | 1b.2 If N/PN/NI to 1b.1: Is it likely that selection of individual participants was affected by knowledge of the intervention assigned to the cluster? | No | All individual participants (patients) approaching the need for dialysis or receiving maintenance dialysis at one of the CKD programs in the intervention group would have participated in the trial (i.e., intervention was designed to impact entire CKD programs). Therefore, the selection of individual participants would not be affected by knowledge of the intervention assigned to the cluster. |
| | 1b.3 Were there baseline imbalances that suggest differential identification or recruitment of individual participants between intervention groups? | No | Covariate-constrained randomization was used to help ensure key baseline characteristics were balanced between intervention groups. |
| | **Risk of bias judgement** | **Low** | |
| Bias due to deviations from intended interventions | 2.1a Were participants aware that they were in a trial? | Partial Yes | This trial was delivered as part of the Ontario Renal Network's (part of Ontario Health) provincial quality improvement strategy to improve access to kidney transplant. Individual patient consent is not required to execute these strategies. Since our unit of randomization was at the cluster and the intervention was at the cluster level it made it impractical to let each participant know about the trial given much of the intervention involved components that may not directly affect them (e.g., administrative support provided to the program and quality improvement teams reviewing transplant metrics). The participants could also opt out of many of the intervention components. For example, patients did not have to participate in the Transplant Ambassador Program if it was not of interest to them and they could decide not to receive education on transplantation.<br><br>Patients could not opt out of trial data collection with the de-identified baseline and outcome data coming from administrative healthcare databases at ICES, which allows for the collection of personal health information without consent for the purpose |
| | 2.1b If Y/PY/NI to 2.1a: Were participants aware of their assigned intervention during the trial? | Partial Yes | |
| | 2.2 Were carers and people delivering the interventions aware of participants' assigned intervention during the trial? | Yes | |

© 2023 Garg AX et al. *JAMA Internal Medicine.*

| | | | |
|---|---|---|---|
| | | | of analysis or compiling of statical information with respect to the management of, evaluation or monitoring of, the allocation of resources to or planning for all or part of the health system.<br><br>The Ontario Renal Network will share the results with all the 26 Chronic Kidney Disease programs in Ontario who can then share this information with staff and patients.<br><br>Since the intervention was at the cluster-level all patients within the CKD programs would have received the intervention and all staff (e.g., clinicians and healthcare professionals) within the CKD programs were aware of the intervention. |
| | 2.3 If Y/PY/NI to 2.1b or 2.2: Were there deviations from the intended intervention that arose because of the trial context? | Yes | The onset of the COVID-19 pandemic, 2.4 years into the 4.2-year trial period, substantially impacted intervention delivery for at least a year. Transplant activity ceased temporarily, local quality improvement teams met less often, there was a pause on the provincial rounds, healthcare staff retired or were re-deployed, and transplant ambassadors transitioned from in-person to virtual meetings.<br><br>Effects of the pandemic on intervention delivery will be explored in the process evaluation.<br><br>In summary, the trial context did not result in deviations from the intended intervention but rather the delivery of the intervention was affected by extenuating circumstances. Please refer to the CONSERVE statement below. |
| | 2.4 If Y/PY to 2.3: Were these deviations likely to have affected the outcome? | NA | Please refer to the CONSERVE statement below. |
| | 2.5 If Y/PY/NI to 2.4: Were these deviations from intended intervention balanced between groups? | NA | Please refer to the CONSERVE statement below. |
| | 2.6 Was an appropriate analysis used to estimate the effect of assignment to intervention? | Yes | Results were analysed using an intention-to-treat approach. Only a small proportion of patients changed clusters during the trial period. |
| | 2.7 If N/PN/NI to 2.6: Was there potential for a substantial impact (on the result) of | NA | |

© 2023 Garg AX et al. *JAMA Internal Medicine.*

| | | | |
|---|---|---|---|
| | the failure to analyse participants in the group to which they were randomized? | | |
| | **Risk of bias judgement** | **Low** | |
| Bias due to missing outcome data | 3.1a Were data for this outcome available for all clusters that recruited participants? | Yes | Outcome data was obtained from provincial administrative healthcare databases at ICES which contains data for all individuals in Ontario with a valid Ontario Health Insurance Plan number. |
| | 3.1b Were data for this outcome available for all, or nearly all, participants within clusters? | Yes | The proportion of missing outcome data in this trial was minimal. |
| | 3.2 If N/PN/NI to 3.1a or 3.1b: Is there evidence that the result was not biased by missing data? | NA | |
| | 3.3 If N/PN to 3.2 Could missingness in the outcome depend on its true value? | NA | |
| | 3.4 If Y/PY/NI to 3.3: Is it likely that missingness in the outcome depended on its true value? | NA | |
| | **Risk of bias judgement** | **Low** | |
| Bias in measurement of the outcome | 4.1 Was the method of measuring the outcome inappropriate? | No | Data was captured from administrative healthcare data which are collected and recorded in a manner that is not influenced by treatment assignment using standardized auditable procedures. Efforts throughout the trial took place to improve and/or verify the accuracy of transplant data. This occurred before and during the trial at all transplant centres regardless of whether the CKD program was in the intervention group or usual-care group. |
| | 4.2 Could measurement or ascertainment of the outcome have differed between intervention groups? | No | |
| | 4.3a If N/PN/NI to 4.1 and 4.2: Were outcome assessors aware that a trial was taking place? | No | |
| | 4.3b If Y/PY/NI to 4.3a: Were outcome assessors aware of the intervention received by study participants? | NA | |
| | 4.4 If Y/PY/NI to 4.3b: Could assessment of the outcome have been influenced by knowledge of intervention received? | NA | |
| | 4.5 If Y/PY/NI to 4.4: Is it likely that assessment of the outcome was influenced by knowledge of intervention received? | NA | |
| | **Risk of bias judgement** | **Low** | |
| Bias in selection of the reported result | 5.1 Were the data that produced this result analysed in accordance with a pre-specified analysis plan that was finalized before | Yes | The pre-specified protocol and statistical analysis plan was published and posted on clinicaltrials.gov before the unblinded outcome data was available. |

© 2023 Garg AX et al. *JAMA Internal Medicine.*

| | | | |
|---|---|---|---|
| | unblinded outcome data were available for analysis? | | Any changes were publicly documented in the table of protocol updates located on clincialtrialsl.gov and these changed occurred before unblinded outcome data was available. |
| | 5.2 ... multiple eligible outcome measurements (e.g. scales, definitions, time points) within the outcome domain? | No | Our outcomes were pre-specified in our published trial protocol and statistical analysis plan. They were captured using administrative health databases. When outcomes could be assessed at multiple time points we pre-specified how the outcome would be captured. Our primary and secondary outcomes were routinely collected in our administrative healthcare databases. |
| | 5.3 ... multiple eligible analyses of the data? | No | The published statistical analysis plan and trial protocol pre-specified multiple analyses of the data. These were all reported and for any outcomes that were not reported a rationale was provided. |
| Overall bias | **Risk of bias judgement** | **Low** | |

[a] Sterne JAC, Savović J, Page MJ, et al. RoB 2: a revised tool for assessing risk of bias in randomised trials. *BMJ*. 2019;366.

© 2023 Garg AX et al. *JAMA Internal Medicine.*

**eTable 4**. CONSERVE-CONSORT (CONSORT Extension for Randomized Controlled Trials Revised in Extenuating Circumstance)

| **CONSERVE-CONSORT Extension: March 2, 2023** | | | |
|---|---|---|---|
| **Item** | **Item Title** | **Description** | **Page No.** |
| I. | Extenuating Circumstances | Describe the circumstances and how they constitute extenuating circumstances. | Manuscript, section-Methods (Statistical Analysis) |
| II. | Important Modifications | a. Describe how the modifications are important modifications. | Published Study Protocol: Methods (Timeline)<br><br>Manuscript, section-Results (Intervention Uptake); Discussion |
| | | b. Describe the impacts and mitigating strategies, including their rationale and implications for the trial. | (see below) |
| | | c. Provide a modification timeline. | Published Study Protocol: Methods (Intervention)<br><br>Protocol compilation (Table of Updates) |
| III. | Responsible Parties | State who planned, reviewed and approved the modifications. | EnAKT LKD investigators approved modifications. |
| IV. | Interim data | If modifications were informed by trial data, describe how the interim data were used, including whether they were examined by study group, and whether the individuals reviewing the data were blinded to the treatment allocation. | Not applicable |
| **CONSORT Number and Item** | | For each row, if important modifications occurred check "direct impact" and/or "mitigating strategy" and describe the changes in the trial manuscript or supplement. Check "no change" for items that are unaffected in the extenuating circumstance. | **Page No.** |

© 2023 Garg AX et al. *JAMA Internal Medicine.*

| | | No Change | Impact* | Mitigating Strategy** | |
|---|---|---|---|---|---|
| 1 | Title and abstract | X | | | |
| 2 | Introduction | X | | | |
| 3 | Methods: Trial Design | X | | | |
| 4 | Methods: Participants | X | | | |
| 5 | Methods: Interventions | | | x | Published study protocol: Methods (Intervention) (Timeline) |
| 6 | Methods: Outcomes | x | | | |
| 7 | Methods: Sample Size | X | | | |
| 8-10 | Methods: Randomisation | X | | | |
| 11 | Methods: Blinding | X | | | |
| 12 | Methods: Statistical methods | X | | | |
| 13 | Results: Participant flow | X | | | |
| 14 | Results: Recruitment | X | | | |
| 15 | Results: Baseline data | X | | | |
| 16 | Results: Numbers analysed | X | | | |

© 2023 Garg AX et al. *JAMA Internal Medicine.*

| | | | | | |
|---|---|---|---|---|---|
| 17 | Results: Outcomes and estimation | | x | x | Manuscript, section- Results (Intervention Uptake) (Additional Analyses)<br><br>The effect of the pandemic on the results will also be analysed and reported in our process evaluation (see Published Process Evaluation protocol).<br><br>Published Statistical Analysis Plan: Methods (Adherence and Protocol Deviations) (Restricting the trial to the pre-COVID period) |
| 18 | Results: Ancillary analyses | x | | | |
| 19 | Results: Harms | X | | | |
| 20 | Discussion: Limitations | | x | | Discussion |
| 21 | Discussion: Generalisability | X | | | |
| | Other information: Registration | X | | | |
| 24 | Other information: Protocol | | x | x | Protocol compilation- Table of Updates |
| 25 | Other information: Funding | X | | | |

© 2023 Garg AX et al. *JAMA Internal Medicine.*

| |
|---|
| *Aspects of the trial that are directly affected or changed by the extenuating circumstance and are not under the control of investigators, sponsor or funder.<br>**Aspects of the trial that are modified by the study investigators, sponsor, or funder to respond to the extenuating circumstance or manage the direct impacts on the trial. |

Orkin AM, Gill PJ, Ghersi D, *et al*. Guidelines for Reporting Trial Protocols and Completed Trials Modified Due to the COVID-19 Pandemic and Other Extenuating Circumstances The CONSERVE 2021 Statement. *JAMA*. 2021;326:257-65. doi:10.1001/ jama.2021.9941

© 2023 Garg AX et al. *JAMA Internal Medicine.*

**eTable 5**. Missing baseline data

Baseline data were missing as follows:

The albumin-to-creatinine ratio and the 2-year risk of kidney failure were missing for 130 (1.3%) (intervention 98 [1.9%], usual care 32 [0.6%]). No imputation was done, and only complete data are reported.

Rural residence was missing for 55 (0.3%) (intervention 26 [0.3%], usual care 29 [0.3%]). Imputed as non-rural [i.e., urban].

Lower income was missing for 74 (0.4%) (intervention 45 [0.5%], usual care 29 [0.3%]). Imputed as 'higher income'.

Ontario Marginalization Index data were missing for 550 (2.7%) (intervention 201 [2.1%], usual care 349 [3.3%]). Imputed as lower category.

Height, weight, and body mass index were missing for 1857 (9.1%) (intervention 1003 [10.3%], usual care 854 [8.1%]). No imputation was done, and only complete data are reported.

All other variables were complete.

© 2023 Garg AX et al. *JAMA Internal Medicine.*

**eTable 6**. Outcome ascertainment, data cleaning, and handling of missing data for the steps completed toward receiving a kidney transplant

| |
|---|
| Information on steps completed toward receiving a kidney transplant by patients with advanced chronic kidney disease (CKD) in Ontario, Canada, was primarily obtained from the Trillium Gift of Life Network (TGLN) database housed at ICES.<br><br>There were 4 steps in this trial.<br><br>Step 1: Referred to a transplant center for evaluation.<br>Step 2: Had a potential living donor contact a transplant center for evaluation.<br>Step 3: Added to deceased donor waitlist.<br>Step 4: Received a transplant from a living or deceased donor.<br><br>Transplant center personnel are requested to accurately input this data into the TGLN database. TGLN performs data validation on some steps; if data discrepancies are identified, they are resolved by asking staff at the transplant centers to verify or correct the discrepancies. Despite these efforts, data errors may still occur.<br><br>We reviewed, quantified, and reconciled data discrepancies in the TGLN dataset before performing any outcome analyses. We explored the completeness of the TGLN data by looking at the total number of patients who completed each step by year and by the transplant center, and we checked whether all expected transplant steps were present and in the correct order. For example, all patients who received a deceased kidney transplant were referred to a transplant center and then placed on the deceased donor transplant waitlist. Similarly, before receiving a living donor transplant, patients need to have a referral and have a potential living donor contact a transplant center for evaluation (where the order of the two steps does not matter). Furthermore, activities for a current transplant had to occur after a prior kidney transplant, should one exist.<br><br>We also needed to ensure that patients would start in the correct state shown visually in eFigure 1a and 1b (patients were allowed to complete steps 1 to 3 before trial entry). Finally, we also needed to ensure we captured new steps after each patient entered the trial.<br><br>We used the following process to assess and correct suspected data entry errors using supplementary data sources, and to impute missing steps if there was sufficient evidence that the patient completed the step (for example if a patient received a kidney transplant but was missing a record of being referred to a transplant center). As described below, we imputed missing steps in the TGLN dataset before linking it to our trial cohort.<br><br>1. We linked the TGLN data to other data sources at ICES to identify any missing historic transplant activity. For example, we examined TGLN transplant data linked to the Ontario Health Insurance Plan (OHIP) database to identify patients with a prior kidney transplant and to differentiate new transplant-related activities from historical activities in patients with a prior failed kidney transplant.<br><br>2. If information on when a potential living donor began their evaluation at a transplant center was missing, we used an additional TGLN date field based on the date the donor first contacted a transplant center (e.g., email or phone call to a transplant center).<br><br>3. The Canadian Organ Replacement Registry (CORR) database contains waitlist information for individuals who went on to receive a transplant. If the TGLN database did not contain a waitlist date for a patient who received a kidney transplant from a deceased donor, we obtained this information from CORR, if it was available.<br><br>4. Some patients with a record of receiving a kidney transplant had missing steps leading up to the transplant that we could not supplement using other data sources. In these cases, we used median imputation conditional on the 6 transplant centers, transplant type (deceased vs. living), and transplant |

© 2023 Garg AX et al. *JAMA Internal Medicine.*

history (i.e., whether there was evidence of at least one prior transplant). We did this to account for potential differences across centers, transplant types, and history. For example, we used median imputation for patients with a record of waitlisting who were missing a transplant center referral date, which was conditional on transplant history and the transplant center where the patient was listed. We calculated the median days between dates (i.e., time from referral to transplant, referral to a waitlist, waitlist to transplant, contact to transplant) for records where both date variables were available. When two or more dates existed, we calculated the median proportion of time between the three dates to ensure that the order was appropriate with an expected alignment.

Imputation was done for all patients in the trial, using the same approach without considering whether they were in the intervention or usual-care group. Once the data were imputed, we proceeded with the trial outcome analyses.

Concerning the number of data errors and the amount of imputation performed for the 20 375 patients accrued into the trial:

- 33 patients (0.16%) had evidence of a waitlist date before their referral date;
- 275 patients (1.35%) had a missing referral date (i.e., the patient had evidence of a waitlist, deceased or living kidney transplant with no evidence of a referral date);
- 92 patients (0.45%) had a missing waitlist date (i.e., the patient had evidence of a deceased kidney donor transplant with no evidence of a waitlist date); and
- 68 patients (0.33%) had a missing date for when a living kidney donor began their evaluation (i.e., the patient had evidence of a living kidney donor transplant but no date for the start of the living kidney donor evaluation).

In summary, for outcome data, 2.1% of patients had evidence of at least one missing step in error, which we then imputed.

© 2023 Garg AX et al. *JAMA Internal Medicine.*

**eTable 7.** The intracluster correlation coefficient (ICC)[a] and coefficient of variation for primary and secondary outcomes

Step 1: Referred to a transplant center for evaluation.
Step 2: Had a potential living donor contact a transplant center for evaluation. [b]
Step 3: Added to deceased donor waitlist.
Step 4: Received a transplant from a living or deceased donor.

| Variable | Patients, No.[c] | Patients with at least 1 event during the trial, No. | Patients with at least 1 event during the trial, % | Unadjusted ICC based on binary censoring indicator | Adjusted ICC[d] based on the binary censoring indicator | Unadjusted coefficient of variation (CV)[e] |
|---|---|---|---|---|---|---|
| **Primary outcome** | | | | | | |
| Steps 1, 2, 3 or 4 | 20 375 | 6520 | 32.0% | 0.023 | 0.010 | 0.238 |
| **Secondary outcomes** | | | | | | |
| Steps 2 or 4, restricted to living donor transplants | 20 375 | 2207 | 10.8% | 0.011 | 0.006 | 0.326 |
| Step 2 | 19 239[f] | 1843 | 9.6% | 0.010 | 0.004 | 0.346 |
| Steps 1 and 2 | 16 369[g] | 1368 | 8.4% | 0.010 | 0.005 | 0.318 |
| Step 4, restricted to living donor transplants | 20 375 | 760 | 3.7% | 0.007 | 0.004 | 0.391 |
| Step 4, restricted to pre-emptive living donor transplants[h] | 10 350[i] | 242 | 2.3% | 0.008 | 0.004 | 0.543 |

[a] The methodology for deriving the ICC from multistate models is limited. For example, from the literature for survival anlaysis, Kalia et al. (2016) show negative biases when using the binary censoring indicator or the continuous event times to calculate the ICC. Nevertheless, we decided to use the binary censoring indicator as recommended by Campbell and Walters (2014) as we found more than minimal censoring (>5%). Furthermore, when there were multiple states, we created a binary indicator based on at least one event. Finally, we calculated ICCs from a Restricted Maximum Likelihood (REML) from a mixed model to naturally account for the variation in cluster sizes.

[b] Only the first potential donor was counted for a patient when there were multiple potential donors.

[c] As there was no intervention effect, we provided a single estimate for ICC and CV across the entire trial group. Had we observed an intervention effect, we would have reported the ICC separately by the intervention and usual-care groups.

[d] As with the primary adjusted analysis, the following baseline characteristics were included when estimating the adjusted ICC: age, sex, Charlson Comorbidity Index, ≥1 intensive care unit admissions in the prior year, the frequency of hospital admissions in the prior year, the historic rate of kidney transplant, whether a transplant center was co-located with the CKD program (present in 6 of the 26 CKD programs), and the CKD treatment modality at the time of trial entry (i.e., in-center hemodialysis, home dialysis, or approaching the need for dialysis). We included the historic transplant center referral pattern as an additional adjustment rather than inclusion as a stratification factor. This measure is provided to demonstrate that some of the variation can be explained by the characteristics of patients within clusters. For planning future trials, we recommend using the more conservative estimates.

© 2023 Garg AX et al. *JAMA Internal Medicine.*

[e] The CV is calculated based on Hayes and Moulton (2017). This method uses the cluster level rates (events per patient-years), where the events in the numerator are not limited to 1 per patient.

[f] Excludes patients who completed step 2 before entering the trial.

[g] Excludes patients who completed steps 1 or 2 before entering the trial.

[h] The follow-up time was censored if and when a patient started dialysis.

[i] Excludes patients who were receiving maintenance dialysis when they entered the trial.

References:

Campbell MJ, Walters SJ. How to Design, Analyse and Report Cluster Randomised Trials in Medicine and Health Related Research. 2014. John Wiley & Sons, Ltd. DOI:10.1002/9781118763452.

Hayes RJ, Moulton LH. Cluster Randomised Trials 2nd edition. 2017. Chapman and Hall/CRC. Doi: 10.4324/9781315370286

Kalia S, Klar N, Doner A. On the estimation of intracluster correlation for time-to-event outcomes in cluster randomized trials. Statistics in Medicine. 2016. 35(39):5551-5560. Doi: 10.1002/sim.7145.

© 2023 Garg AX et al. *JAMA Internal Medicine.*

**eTable 8**. Multistate model assumptions

| |
|---|
| Primary outcome: The semiparametric multistate model for the primary outcome was assessed using a constrained intervention effect with cluster-level bootstrap standard errors (to account for clustering on outcomes within chronic kidney disease [CKD] programs) and with a t-distribution to adjust the degrees of freedom as a small sample correction when calculating the margin of error in the 95% CI because there were less than 40 clusters in the trial. The resulting estimand is an individual-averaged cause-specific hazard ratio for the total effect of the intervention on the key steps towards transplantation. |
| We evaluated the model assumptions in the multistate model. Decisions on how to manage any violation in assumptions were made prior to knowledge of the results and considered (1) impact to the statistical interpretation of the intervention effect, (2) overall model fit (i.e., model improvement), (3) rules of parsimony between two statisticians (Dr. Dixon and Dr. Luo), and (4) clinical interpretation through discussions with the clinical lead (Dr. Garg).<br>The following are details on the model assumptions.<br>1. **Markov:** The Markov assumption in multistate models assumes that transition intensities only depend on the information in the current state at a particular time. To test this assumption, we incorporated event history variables in the model in a few ways (i.e., time spent since completing a prior step at time of state entry, or time to complete a prior step in history; each was also further assessed as time during the trial, prior to trial start, and overall). Regardless of the way in which the history was defined, there was an indication that the Markov assumption was violated. As such, our final selected models included the historic variables in the model. It is worth noting that when specifying the history in more detail, some of the historical variables were not significant. Although the model showed improvements in overall fit with a more detailed information of history, the intervention effect remained unchanged regardless of how the event history was defined. We chose to keep the simplest summary of history (adding only 3 additional terms to the model based on time spent since completing the prior 3 steps at the time of state entry) because there was no impact to the main findings and due to rules of parsimony.<br>2. **Linearity:** We had 4 continuous variables in the model: age, the historic rate of kidney transplant, the Charlson Comorbidity Index, and the frequency of hospital admissions in the prior year. We tested the assumption of linearity for each variable using restricted cubic splines and B-splines. We assessed the impact to the model (i.e., change in intervention effect, significance and overall model fit). We found violations in linearity for age and historic rate of kidney transplant. However, we chose to not include the splines in our final model because the interpretation of the intervention effect remained unchanged, and because of rules of parsimony.<br>3. **Proportional Hazards:** Our multistate model used a semiparametric framework where no assumptions are made about the baseline hazard (i.e., transition intensity). This approach assumes proportional hazards (PH) in our model variables. We tested the PH assumption in the variables included in our model using the Schoenfeld residuals [Reference: Fox and Weisberg (2023)[a]]. The PH assumption was satisfied for the intervention effect in models for the primary and secondary outcomes. In the primary model, we did find PH violations for age (p-value < 0.001), baseline cluster historic rate of kidney transplant (p-value < 0.001), CKD treatment modality at time of trial entry (p-value 0.002), Charlson Comorbidity Index (p-value < 0.001), and whether a transplant center was co-located with the CKD program (present in 6 of the 26 CKD programs) (p-value 0.034). We did not include time-interactions to the adjusted factors that violated the proportional hazard assumption because our estimated intervention effect was robust regardless of changes to the model.<br>4. **Constrained intervention effect:** Our primary model reported the constrained intervention effect. We assumed an overall effect of the intervention across all transitions between states (rather than the specific effects from individual transitions between states). We present results of the unconstrained model in eTable 11. The unconstrained model has better model fit than the constrained model. |

© 2023 Garg AX et al. *JAMA Internal Medicine.*

5. **Independence:** There is a natural correlation of outcomes within CKD programs induced by the clustered design which violates the independence assumption. To accommodate, variance inflation techniques are required to avoid spurious statistical significance. The inferences in the paper (i.e., p-values and confidence intervals) were calculated using a cluster-level bootstrap standard error and a *t*-distribution to adjust the degrees of freedom from the small number of clusters included in the trial (note: this approach has not been formally tested and may deviate from the nominal levels; i.e., wider or narrower intervals than expected). The bootstrap approach inflates the variance beyond a model that assumes independence or a model that applies a robust standard error without a small sample correction.
6. **Non-informative censoring:** There were competing events in our follow-up period that could impact the probability of observing our events of interest (i.e., steps toward transplantation). The main competing events were death and recorded contradiction to transplant, neither of which differed between the arms of the trial. We choose to censor at these events, and therefore, the model produced an estimand of the individual-averaged cause-specific hazard ratio for the total effect of the intervention on the key steps towards transplantation. We acknowledge that the estimate could be mediated by the competing events and that the total intervention effect does not provide information about whether the intervention effect on key steps is partially driven by the intervention effect on the competing risks. However, we explored the impact of the intervention on the competing risks and did not find any associations.

[a] Fox and Weisberg (2023). Cox Proportion-Hazards Regression for Survival Data in R, an appendix to an R companion to applied regression 3rd edition.

© 2023 Garg AX et al. *JAMA Internal Medicine.*

**eTable 9**. Patient follow-up

| | Intervention | Usual care |
|---|---|---|
| Number of programs | 13 | 13 |
| Number of patients per program, mean (SD) | 752 (420) | 815 (463) |
| Number of patients per program, median (IQR) | 789 (535-912) | 738 (591-995) |
| Number of patients | 9780 | 10 595 |
| Patient-years of follow-up | 21 470 | 23 430 |
| Patient follow-up, mean (SD), years | 2.2 (1.4) | 2.2 (1.4) |
| Patient follow-up, median (IQR), years | 2.0 (1.0, 3.5) | 2.1 (1.0, 3.6) |
| **Events in follow-up** | | |
| Number of patients who emigrated from Ontario, n (%) | 136 (1.4) | 154 (1.4) |
| Rate of emigration, 100 patient-years* | 0.6 | 0.7 |
| Number of patients who recovered kidney function, n (%) | 92 (0.9) | 133 (1.1) |
| Rate of recovered kidney function, 100 patient-years* | 0.4 | 0.6 |
| Number of patients who became ineligible to receive a kidney transplant[a], n (%) | 1351 (13.8) | 1438 (13.6) |
| Rate of becoming ineligible to receive a kidney transplant, 100 patient-years* | 6.3 | 6.1 |
| Number of patients who died, n (%) | 1624 (16.6) | 1808 (17.1) |
| Rate of death, 100 patient-years* | 7.6 | 7.7 |
| Number of patients who received a kidney transplant, n (%) | 1214 (12.4) | 1233 (11.6) |
| Rate of receiving a kidney transplant, 100 patient-years* | 5.7 | 5.3 |
| Number of patients who reached trial end date (Dec 31, 2021) without event | 5363 | 5829 |
| **Transfers to another CKD program in follow-up** | | |
| Number of patients who switched to CKD program with same allocation | 467 | 416 |
| Rate of switches, per 100 patient-years | 2.2 | 1.8 |
| Number of patients who switched to CKD program with opposite allocation | 484 | 621 |
| Rate of switches, per 100 patient-years | 2.3 | 2.8 |
| **Average time in each state, years[b]** | | |
| No steps | 1.8 | 1.7 |
| Referral | 1.5 | 1.5 |
| Donor evaluation | 0.6 | 0.6 |
| Referral and donor evaluation | 1.2 | 1.2 |
| Referral and waitlist | 1.1 | 1.1 |
| Referral, waitlist, and donor evaluation | 1.1 | 1.2 |

The primary analysis used an intention-to-treat approach. All outcomes were attributed to the program where a patient entered the trial, regardless of whether they transferred to another program in follow-up. As the primary outcome was ascertained using provincial healthcare databases, the only reason for lost follow-up was emigration from Ontario.

* We estimated the intervention on each of the competing risks and did not find an association in either the unadjusted or adjusted analyses. The adjusted hazard ratios (95% confidence intervals) are as follows: emigration: 1.00 (0.81, 1.25); recovered kidney function: 0.89 (0.57,1.39); developed contraindication to receiving a transplant: 1.00 (0.87, 1.14); death: 0.96 (0.87, 1.07). We also looked at a composite outcome of all competing events other than death: 0.99 (0.89, 1.10). The proportional hazard assumption was not violated for the intervention effect in any of these analyses.

[a] Developed a contraindication to receiving a transplant that was recorded in our data sources; for example, a diagnosis of dementia, use home oxygen [a sign of serious lung disease], transfer to a long-term care home, or developed a comorbidity likely to preclude transplantation.

© 2023 Garg AX et al. *JAMA Internal Medicine.*

[b] The states listed here are shown visually in eFigure 1a and 1b. The average time is the mean patient time spent in a certain state until transitioning into another state or being censored during the trial period. For example, patients receiving the intervention have an average time spent in the ‘no steps’ state of 1.8 years. We did not provide the mean sojourn time as it is an estimate relying on strict parametric assumptions, and our main model used a semi-parametric framework.

© 2023 Garg AX et al. *JAMA Internal Medicine.*

**eTable 10**. Primary outcome examined in two multistate models: the first unadjusted for baseline characteristics and the second adjusted for additional baseline characteristics

| Analysis | Patients, No. | | Steps completed during the trial, No. | | Rate of steps completed per 100 patient-years | | Hazard Ratio (95% CI)[a] |
|---|---|---|---|---|---|---|---|
| | Intervention | Usual care | Intervention | Usual care | Intervention | Usual care | |
| Primary analysis[b] | 9780 | 10 595 | 5334 | 5638 | 24.8 | 24.1 | 1.00 (0.87-1.15) |
| Unadjusted | 9780 | 10 595 | 5334 | 5638 | 24.8 | 24.1 | 1.03 (0.88-1.20) |
| Additional adjustment[c] | 9780 | 10 595 | 5334 | 5638 | 24.8 | 24.1 | 1.00 (0.85-1.18) |

[a] For the hazard ratio, the referent group is usual care. We used a stratified, constrained, multistate model accounting for the order in which steps were completed and the clustered design. The historic transplant center referral pattern was a stratification factor in both the randomization and our final model. To maintain valid inferences, we used cluster-level bootstrapping to obtain standard errors (accounting for the correlated outcomes within CKD programs) and a t-distribution as a small-sample correction when calculating the margin of error in the 95% CI because our trial included <40 clusters.

[b] The following baseline characteristics were included in the model: age, sex, Charlson Comorbidity Index, ≥1 intensive care unit admissions in the prior year, the frequency of hospital admissions in the prior year, the historic rate of transplant, whether the transplant center was co-located with a CKD program (present in 6 of the 26 CKD programs), and the CKD treatment modality at the time of trial entry (i.e., in-center hemodialysis, home dialysis, or approaching the need for dialysis).

[c] Besides the baseline characteristics used for adjustment listed in the primary analysis [b], two additional baseline characteristics were added based on their distribution in Table 1 in the main paper: one-way distance from residence to a transplant center (as a continuous variable), and higher residential instability (as a binary variable).

© 2023 Garg AX et al. *JAMA Internal Medicine.*

**eTable 11**. Effect of the intervention on each transition (shown visually in eFigure 1a and 1b); these estimates are from an unconstrained model

Step 1: Referred to a transplant center for evaluation.
Step 2: Had a potential living donor contact a transplant center for evaluation.
Step 3: Added to deceased donor waitlist.
Step 4: Received a transplant from a living or deceased donor.

| | Patients available for this transition, No. | | Transitions completed during the trial, No. | | Transition rate per 100 patient-years | | Adjusted Hazard Ratio (95% CI)[b] |
|---|---|---|---|---|---|---|---|
| | Intervention | Usual care | Intervention | Usual care | Intervention | Usual care | |
| **Transition** | | | | | | | |
| 1) No activity to step 1 | 7828 | 8541 | 1631 | 2040 | 11.7 | 13.7 | 0.97 (0.75–1.26) |
| 2) No activity to step 2 | 7828 | 8541 | 347 | 206 | 2.5 | 1.4 | 1.79 (1.02–3.17) |
| 3) Step 1 to steps 1 & 2 | 2761 | 3266 | 526 | 647 | 12.9 | 13.1 | 1.11 (0.77–1.59) |
| 4) Step 1 to steps 1 & 3 | 2761 | 3266 | 669 | 734 | 16.4 | 14.9 | 1.00 (0.79–1.25) |
| 5) Step 2 to steps 1 & 2 | 485 | 299 | 387 | 240 | 135.4 | 134.3 | 0.83 (0.49–1.40) |
| 6) Step 1 & 2 to steps 1, 2, & 3 | 1254 | 1254 | 510 | 471 | 35.0 | 30.1 | 1.00 (0.67–1.50) |
| 7) Step 1 & 2 to steps 1, 2, & 4 | 1254 | 1254 | 228 | 227 | 15.7 | 14.5 | 1.01 (0.63–1.63) |
| 8) Step 1 & 3 to steps 1, 2, & 3 | 921 | 996 | 50 | 67 | 4.9 | 6.2 | 0.80 (0.47–1.35) |
| 9) Step 1 & 3 to steps 1, 3, & 4 | 921 | 996 | 592 | 599 | 57.8 | 55.0 | 0.96 (0.86–1.07) |
| 10) Step 1, 2, & 3 to steps 1, 2, 3 & 4 | 651 | 644 | 394 | 407 | 53.4 | 54.3 | 0.87 (0.66–1.14) |

[a] Only the first potential donor was counted for a patient when there were multiple potential donors.

[b] For the adjusted hazard ratio, the referent group is usual care. We used a stratified, multistate model accounting for the order in which steps were completed and the clustered design. The historic transplant center referral pattern was a stratification factor in both the randomization and our final model. We also stratified on the different transitions between steps to allow for separate baseline hazard functions. To maintain valid inferences, we used cluster-level bootstrapping to obtain standard errors (accounting for the correlated outcomes within CKD programs) and a t-distribution as a small-sample correction when calculating the margin of error in the 95% CI because our trial included <40 clusters. The following baseline characteristics were included in the model: age, sex, Charlson Comorbidity Index, ≥1 intensive care unit admissions in the prior year, the frequency of hospital admissions in the prior year, the historic rate of transplant, whether a transplant center was co-located with the CKD program (present in 6 of the 26 CKD programs), and the CKD treatment modality at the time of trial entry (i.e., in-center hemodialysis, home dialysis, or approaching the need for dialysis).

© 2023 Garg AX et al. *JAMA Internal Medicine.*

**eTable 12**. Effect of the intervention on steps not specified as primary or secondary outcomes

Step 1: Referred to a transplant center for evaluation.
Step 2: Had a potential living donor contact a transplant center for evaluation.[a]
Step 3: Added to deceased donor waitlist.
Step 4: Received a transplant from a living or deceased donor.

| Outcome | Patients, No. | | Steps completed during the trial, No. | | Rate of steps completed per 100 patient-years | | Adjusted hazard ratio (95% CI)[d] |
|---|---|---|---|---|---|---|---|
| | Intervention | Usual care | Intervention | Usual care | Intervention | Usual care | |
| Step 1 | 7966[b] | 8634[b] | 2018 | 2280 | 14.2 | 15.1 | 1.02 (0.79-1.32) |
| Step 3 | 9437[c] | 10 227[c] | 1179 | 1205 | 6.0 | 5.6 | 1.06 (0.83-1.36) |
| Step 4 | 9780 | 10 595 | 1214 | 1233 | 5.7 | 5.3 | 1.05 (0.86-1.27) |
| Step 4, restricted to deceased donor transplants | 9780 | 10 595 | 833 | 854 | 3.9 | 3.6 | 1.05 (0.86-1.27) |

[a] Only the first potential donor was counted for a patient when there were multiple potential donors.

[b] Excludes patients who completed step 1 before entering the trial.

[c] Excludes patients who completed step 3 before entering the trial.

[d] For the adjusted hazard ratio, the referent group is usual care. Outcomes in this trial were analyzed at the patient level using a stratified, constrained, multistate model accounting for the order in which steps were completed, the clustered design, and the covariates used in the randomization. The multistate model for the outcomes in this table reduced to a classic Cox proportional hazards model (i.e., a single endpoint). The historic transplant center referral pattern was a stratification factor in both the randomization and our final model. To maintain valid inferences, we used cluster-level bootstrapping to obtain standard errors (accounting for the correlated outcomes within CKD programs) and a t-distribution as a small-sample correction when calculating the margin of error in the 95% CI because our trial included <40 clusters. The following baseline characteristics were included in the model: age, sex, Charlson Comorbidity Index, the historic rate of kidney transplant, whether a transplant center was co-located with the CKD program (present in 6 of the 26 CKD programs), and the CKD treatment modality at the time of trial entry (i.e., in-center hemodialysis, other forms of dialysis, or approaching the need for dialysis).

© 2023 Garg AX et al. *JAMA Internal Medicine.*

**eTable 13**. Effect of the intervention when follow-up was truncated to March 16, 2020, the start of the COVID-19 pandemic in Ontario.

Step 1: Referred to a transplant center for evaluation.
Step 2: Had a potential living donor contact a transplant center for evaluation.[a]
Step 3: Added to deceased donor waitlist.
Step 4: Received a transplant from a living or deceased donor.

| Variable | Patients, No. | | Completed steps during the trial, No. | | Rate of completed steps per 100 patient-years | | Adjusted Hazard Ratio (95% CI)[b] |
|---|---|---|---|---|---|---|---|
| | Intervention | Usual care | Intervention | Usual care | Intervention | Usual care | |
| **Primary outcome** | | | | | | | |
| Steps 1, 2, 3 or 4 | 7449 | 8052 | 3324 | 3438 | 28.5 | 26.9 | 1.02 (0.89-1.16) |
| **Secondary outcomes** | | | | | | | |
| Steps 2 or 4 | 7449 | 8052 | 824 | 818 | 7.1 | 6.4 | 1.10 (0.90-1.33) |
| Step 2[c] | 6958[d] | 7563[d] | 586 | 593 | 5.6 | 5.2 | 1.15 (0.94-1.41) |
| Steps 1 and 2[c] | 5616[e] | 6153[e] | 377 | 415 | 4.5 | 4.5 | 1.04 (0.81-1.32) |
| Step 4, restricted to living donor transplants[c] | 7449 | 8052 | 238 | 225 | 2.0 | 1.8 | 1.17 (0.79-1.75) |
| Step 4, restricted to pre-emptive living donor transplants[c,f] | 3446[g] | 3539[g] | 77 | 67 | 1.9 | 1.6 | 1.18 (0.63-2.20) |

[a] Only the first potential donor was counted for a patient when there were multiple potential donors.

[b] For the adjusted hazard ratio, the referent group is usual care. The primary outcome was analyzed at the patient-level using a stratified, constrained, multistate model accounting for the order in which steps were completed, the clustered design, and the covariates used in the randomization. The historic transplant center referral pattern was a stratification factor in both the randomization and our final model. We also stratified on the different transitions between steps to allow for separate baseline hazard functions. To maintain valid inferences, we used cluster-level bootstrapping to obtain standard errors (accounting for the correlated outcomes within CKD programs) and a t-distribution as a small-sample correction when calculating the margin of error in the 95% CI because our trial included <40 clusters. The following baseline characteristics were included in the model: age, sex, Charlson Comorbidity Index, the historic rate of kidney transplant, whether a transplant center was co-located with the CKD program (present in 6 of the 26 CKD programs), and the CKD treatment modality at the time of trial entry (i.e., in-center hemodialysis, home dialysis, or approaching the need for dialysis). The secondary outcomes were analyzed using the same multistate model framework and evaluation process as for the primary outcome. For outcomes with a single endpoint, the model reduced to a classic Cox proportional hazards model.

[c] The multistate model for this outcome reduced to a classic Cox proportional hazards model.

[d] Excludes patients who completed step 2 before entering the trial.

[e] Excludes patients who completed steps 1 or 2 before entering the trial.

[f] The follow-up time was censored if and when a patient started dialysis.

[g] Excludes patients who were receiving maintenance dialysis when they entered the trial.

© 2023 Garg AX et al. *JAMA Internal Medicine.*

**eTable 14**. Effect of the intervention on the primary composite outcome in multiple subgroups. The adjusted hazard ratio results are also presented graphically in **eFigure 2**.

| Subgroup | Patients, No. | | Steps completed during the trial, No. | | Rate of steps completed per 100 patient-years | | Adjusted Hazard Ratio (95% CI)[a] |
|---|---|---|---|---|---|---|---|
| | Intervention | Usual care | Intervention | Usual care | Intervention | Usual care | |
| **Type of treatment at trial entry** | | | | | | | |
| Approaching the need for dialysis | 5058 | 5292 | 2638 | 2767 | 23.6 | 23.7 | 1.00 (0.81 to 1.23) |
| In-center hemodialysis | 3495 | 3951 | 1720 | 1831 | 23.1 | 21.4 | 1.01 (0.86 to 1.19) |
| Home dialysis | 1227 | 1352 | 976 | 1040 | 34.1 | 32.7 | 0.97 (0.85 to 1.12) |
| **Sex** | | | | | | | |
| Female | 3726 | 4060 | 2000 | 2090 | 24.4 | 22.7 | 0.99 (0.82 to 1.20) |
| Male | 6054 | 6535 | 3334 | 3548 | 25.1 | 24.9 | 1.01 (0.90 to 1.14) |
| **One-way distance from residence to transplant center, km** | | | | | | | |
| 0 to <30 | 5604 | 4987 | 3264 | 2966 | 25.7 | 25.9 | 0.94 (0.42 to 2.08) |
| 30 to <70 | 2247 | 2623 | 1250 | 1390 | 26.0 | 23.8 | 0.96 (0.76 to 1.21) |
| 70 and over | 1929 | 2985 | 820 | 1282 | 20.7 | 21.0 | 1.04 (0.72 to 1.49) |
| **Income quintile**[b] | | | | | | | |
| Lower (quintiles 1, 2) | 5042 | 5343 | 2516 | 2543 | 22.6 | 21.3 | 1.03 (0.89 to 1.18) |
| Higher (quintiles 3, 4, 5) | 4738 | 5252 | 2818 | 3095 | 27.2 | 26.9 | 1.00 (0.84 to 1.19) |
| **Residential Instability**[c] | | | | | | | |
| Lower (quintiles 1, 2, 3) | 4881 | 5962 | 2891 | 3311 | 26.9 | 24.9 | 1.02 (0.91 to 1.14) |
| Higher (quintiles 4, 5) | 4899 | 4633 | 2443 | 2327 | 22.8 | 22.9 | 1.00 (0.81 to 1.22) |
| **Material Deprivation**[d] | | | | | | | |
| Lower (quintiles 1, 2, 3) | 5034 | 5486 | 2992 | 3162 | 27.1 | 26.4 | 0.99 (0.83 to 1.17) |
| Higher (quintiles 4, 5) | 4746 | 5109 | 2342 | 2476 | 22.5 | 21.6 | 1.02 (0.87 to 1.20) |
| **Ethnic diversity**[e] | | | | | | | |
| Lower (quintiles 1, 2, 3) | 4691 | 5306 | 2378 | 2622 | 24.1 | 23.4 | 0.98 (0.79 to 1.21) |
| Higher (quintiles 4, 5) | 5089 | 5289 | 2956 | 3016 | 25.5 | 24.7 | 1.01 (0.88 to 1.16) |
| **Dependency**[f] | | | | | | | |
| Lower (quintiles 1, 2, 3) | 6152 | 6582 | 3589 | 3724 | 26.1 | 24.9 | 0.99 (0.86 to 1.14) |
| Higher (quintiles 4, 5) | 3628 | 4013 | 1745 | 1914 | 22.6 | 22.5 | 1.02 (0.85 to 1.23) |

© 2023 Garg AX et al. *JAMA Internal Medicine.*

| | | | | | | | |
|---|---|---|---|---|---|---|---|
| **Trial entry date** | | | | | | | |
| Nov 1, 2017 | 4162 | 4560 | 2807 | 2838 | 25.3 | 22.8 | 1.04 (0.91 to 1.17) |
| After Nov 1, 2017 | 5618 | 6035 | 2527 | 2800 | 24.3 | 25.5 | 0.96 (0.80 to 1.15) |
| **Age, years**[g] | | | | | | | |
| 18 to 54 | 3041 | 3301 | 2752 | 2861 | 39.8 | 37.1 | 0.99 (0.84 to 1.18) |
| 55 to 65 | 3163 | 3398 | 1720 | 1788 | 24.3 | 23.5 | 1.02 (0.88 to 1.19) |
| 66 to 75 | 3576 | 3896 | 862 | 989 | 11.5 | 12.2 | 0.98 (0.77 to 1.25) |
| **Diabetes mellitus**[g] | | | | | | | |
| Yes | 5390 | 6127 | 2128 | 2479 | 18.6 | 18.9 | 0.99 (0.81 to 1.21) |
| No | 4390 | 4468 | 3206 | 3159 | 32.0 | 30.5 | 1.00 (0.87 to 1.14) |
| **Cardiovascular disease**[g] | | | | | | | |
| Yes | 5298 | 5804 | 2204 | 2310 | 19.7 | 18.8 | 1.03 (0.88 to 1.20) |
| No | 4482 | 4791 | 3130 | 3328 | 30.5 | 29.9 | 0.98 (0.83 to 1.16) |

All subgroups were pre-specified unless indicated otherwise.

The widths of the confidence intervals (CI) for these outcomes were not adjusted for multiplicity, so the CIs should not be used to infer definitive treatment effects for these outcomes.

[a] For the adjusted hazard ratio, the referent group is usual care. Outcomes in this trial were analyzed at the patient-level using a stratified, constrained, multistate model accounting for the order in which steps were completed, the clustered design, and the covariates used in the randomization. The historic transplant center referral pattern was a stratification factor in both the randomization and our final model. We also stratified on the different transitions between steps to allow for separate baseline hazard functions. To maintain valid inferences, we used cluster-level bootstrapping to obtain standard errors (accounting for the correlated outcomes within CKD programs) and a t-distribution as a small-sample correction when calculating the margin of error in the 95% CI because our trial included <40 clusters. The following baseline characteristics were included in the model: age, sex, Charlson Comorbidity Index, the historic rate of kidney transplant, whether a transplant center was co-located with the CKD program (present in 6 of the 26 CKD programs), and the CKD treatment modality at the time of trial entry (i.e., in-center hemodialysis, other forms of dialysis, or approaching the need for dialysis.

[b] Income quintile: measured by neighborhood-level median income

[c] Residential instability refers to the area-level concentration of people with high housing instability or family instability

[d] Material deprivation refers to individuals and communities not being able to access and realize basic material needs.

[e] Ethnic diversity refers to area-level concentrations of residents who are recent immigrants and/or those who self-identify as a visible minority.

[f] Dependency refers to the area-level concentrations of people who do not have employment income, including seniors and children.

[g] Post hoc subgroups

© 2023 Garg AX et al. *JAMA Internal Medicine.*

**eTable 15**. The time to complete steps and other measures in the intervention group and usual-care group.[a] Each result restricted to only those patients who completed all components for the measure during the trial period.

| Time to step completion and other outcomes | Patients, No. | | Median (IQR), months | |
|---|---|---|---|---|
| | Intervention | Usual care | Intervention | Usual care |
| Time from starting maintenance dialysis to transplant center referral[b] | 1057 | 1160 | 7.7 (4.0-13.2) | 7.2 (3.4-12.6) |
| Time from transplant center referral to receipt of a living kidney donor transplant[c] | 178 | 173 | 13.6 (9.3-19.3) | 15.6 (11.3-20.9) |
| Time from transplant center referral to receipt of a transplant (living or deceased)[d] | 412 | 411 | 16.1 (10.9-24.7) | 18.9 (12.2-26.6) |
| Time from when potential living donor began evaluation to living donor transplant[e] | 182 | 214 | 13.8 (9.5-18.5) | 13.9 (9.6-19.1) |
| Time from when potential living donor began evaluation to abdominal CT angiogram[f] | 519 | 495 | 5.2 (3.2-8.6) | 5.1 (3.2-8.6) |
| Time from referral to when a potential living donor began their evaluation[g] | 145 | 154 | 0.1 (0.0-2.1) | 0.5 (0.0-2.6) |
| Time from referral to waitlisting for a deceased donor transplant[h] | 580 | 607 | 11.6 (7.7-17.8) | 14.3 (9.5-21.1) |
| **Referrals and other outcomes** | **Patients, No.** | | **No. (%)** | |
| | **Intervention** | **Usual care** | **Intervention** | **Usual care** |
| Referral to a transplant center that was declined[i] | 2018 | 2280 | 1 to 5 (0.05 to 0.2) | 20 to 25 (0.9 to 1.1) |
| Referral to a transplant center that was accepted[j] | 2018 | 2280 | 1900 (94.2) | 2166 (95.0) |
| Referral to a transplant center that was deferred[k] | 2018 | 2280 | 105 (5.2) | 90 (3.9) |
| Patient referred to a transplant center with no transplant or waitlisting within 1 year[l] | 1419 | 1642 | 1060 (74.7) | 1363 (83.0) |
| Living donor transplant was pre-emptive[m] | 381 | 379 | 127 (33.3) | 115 (30.3) |

[a] The following measures were listed in the protocol but were not analyzed because either the data were of insufficient quality or there was concern that the measure would not provide meaningful information: (1) Time from nephrologist consultation to donor nephrectomy. (2) Proportion of potential living kidney donors who began their evaluation who completed all the following: a nephrology consultation, a surgeon consultation, and an abdominal computed tomography angiogram. (3) Time from when the potential kidney donor began their evaluation to abdominal CT angiogram, restricted to those who had a donor nephrectomy during the trial period. (4) Time from referral to waitlisting for a deceased donor transplant for patients who received a living kidney donor transplant during the trial period. (5) Time from consulting with a transplant nephrologist (after referral to a transplant center) to waitlisting (this measure could also be restricted to patients who received a living kidney donor transplant during the trial period). (6) Time from referral to a transplant center to consulting with a transplant nephrologist (this measure could also be restricted to patients who received a living kidney donor transplant during the trial period). (7) Proportion of referrals to a transplant center that were incomplete. (8) Rate of living kidney donor transplants assessed in patients waitlisted for a deceased donor transplant. The published trial protocol indicated some of measures in this table would be reported during the trial period, while others would be assessed as a change from historical norms pre-dating the trial. For reasons of time, cost, and feasibility we focused on reporting these measures by group only during the trial period.

[b] Assessed in patients who entered the trial receiving maintenance dialysis after November 1, 2017 or those who entered the trial approaching the need for dialysis and started maintenance dialysis during the trial period. In both cases we only considered patients who received a referral after they began maintenance dialysis.

[c] Assessed in patients who had a transplant referral and living kidney donor transplant during the trial period.

[d] Assessed in patients who had a transplant referral and a living or deceased donor transplant during the trial period.

[e] Assessed in patients who had a potential living kidney donor begin their evaluation and who received a living kidney donor transplant during the trial period.

© 2023 Garg AX et al. *JAMA Internal Medicine.*

[f] Assessed in patients who had a potential living kidney donor who began their evaluation, and the donor had an abdominal CT angiogram during the trial period. There are 88.5% (1913/2162) of recipients who had a donor who began their evaluation and at least one of the donors had a valid Ontario health card number (i.e., this analysis excludes potential out of province donors and donors with invalid health card numbers since they could not be linked to our other data sources). This corresponds to 88.4% (956/1081) and 88.5% (957/1081) in the intervention and control groups respectively. Only 53% overall had evidence of a CT angiograph during follow up (1014/1913), corresponding to 54.3% (519/956) and 51.7% (495/957) in the intervention and control groups respectively.

[g] Assessed in patients who had a living donor transplant, a referral and a potential living kidney donor begin their evaluation during the trial period. Potential living kidney donors who began their evaluation before the referral were given a value of 0 months.

[h] Assessed in patients who had a transplant referral and were waitlisted for a deceased donor transplant during the trial period.

[i] Referral declined indicates a patient no longer moves forward with the kidney transplant assessment process (i.e., will not be scheduled to meet with a transplant specialist). Reasons for a declined status, include 1) patient did not meet referral criteria; 2) patient referred to another program; 3) patient was too sick to receive a transplant; or 4) patient declined to consult with a transplant specialist. In accordance with ICES policy of suppressing cell sizes <6, numbers are presented as ranges.

[j] Referral accepted indicates the patient was approved to proceed with their kidney transplant assessment.

[k] Referral deferred indicates that a consultation with a transplant specialist was not made at the time but could be made in the future (within 12 months).

[l] Assessed in patients who were referred to a transplant center during the trial period and had at least a year of follow-up when no transplant or no waitlist was observed within a year of the referral date during the trial.

[m] Assessed in those patients with advanced chronic kidney disease who were approaching the need for dialysis when they entered the trial and were not on dialysis when they received a living kidney donor transplant during the trial period.

© 2023 Garg AX et al. *JAMA Internal Medicine.*

**eTable 16**. Effect of the intervention when restricted to patients who completed no steps toward receiving a transplant before trial entry

Step 1: Referred to a transplant center for evaluation.
Step 2: Had a potential living donor contact a transplant center for evaluation.[a]
Step 3: Added to deceased donor waitlist.
Step 4: Received a transplant from a living or deceased donor.

| Variable | Patients, No. | | Steps completed during the trial, No. | | Rate of steps completed per 100 patient-years | | Adjusted Hazard Ratio (95% CI) [b] |
|---|---|---|---|---|---|---|---|
| | Intervention | Usual care | Intervention | Usual care | Intervention | Usual care | |
| **Primary outcome** | | | | | | | |
| Steps 1, 2, 3 or 4 | 7828 | 8541 | 3560 | 3913 | 20.7 | 20.8 | 1.01 (0.86-1.18) |
| **Secondary outcomes** | | | | | | | |
| Steps 2 or 4 | 7828 | 8541 | 889 | 883 | 5.2 | 4.7 | 1.12 (0.90-1.40) |
| Step 2[c] | 7828 | 8541 | 744 | 729 | 4.6 | 4.1 | 1.20 (0.97-1.48) |
| Steps 1 and 2[c] | 7828 | 8541 | 677 | 691 | 4.2 | 3.9 | 1.16 (0.90-1.49) |
| Step 4, restricted to living donor transplants[c] | 7828 | 8541 | 145 | 154 | 0.8 | 0.8 | 0.98 (0.55-1.73) |
| Step 4, restricted to pre-emptive living donor transplants[d,e] | 4663[e] | 4888[e] | 51 | 44 | 0.7 | 0.6 | 1.63 (0.32-8.33) |

[a] Only the first potential donor was counted for a patient when there were multiple potential donors.

[b] For the adjusted hazard ratio, the referent group was usual care. The primary outcome was analyzed at the patient-level using a stratified, constrained, multistate model accounting for the order in which steps were completed, the clustered design, and the covariates used in the randomization. The historic transplant center referral pattern was a stratification factor in both the randomization and our final model. We also stratified on the different transitions between steps to allow for separate baseline hazard functions. To maintain valid inferences, we used cluster-level bootstrapping to obtain standard errors (accounting for the correlated outcomes within CKD programs) and a t-distribution as a small-sample correction because our trial included <40 clusters. The following baseline characteristics were included in the model: age, sex, Charlson Comorbidity Index, the historic rate of kidney transplant, whether a transplant center was co-located with the CKD program (present in 6 of the 26 CKD programs), and the CKD treatment modality at the time of trial entry (i.e., in-center hemodialysis, home dialysis, or approaching the need for dialysis). The secondary outcomes were analyzed using the same multistate model framework and evaluation process as for the primary outcome. For outcomes with a single endpoint, the model reduced to a classic Cox proportional hazards model.

[c] The multistate model for this outcome reduced to a classic Cox proportional hazards model.

[d] The follow-up time was censored if and when a patient started dialysis.

[e] Excludes patients who were receiving maintenance dialysis when they entered the trial.

© 2023 Garg AX et al. *JAMA Internal Medicine.*

**eTable 17**. Effect of the intervention when restricted to patients who entered the trial approaching the need for dialysis

Step 1: Referred to a transplant center for evaluation.
Step 2: Had a potential living donor contact a transplant center for evaluation.[a]
Step 3: Added to deceased donor waitlist.
Step 4: Received a transplant from a living or deceased donor.

| | Patients, No. | | Steps completed during the trial, No. | | Rate of steps completed per 100 patient-years | | Adjusted Hazard Ratio (95% CI)[b] |
|---|---|---|---|---|---|---|---|
| **Variable** | **Intervention** | **Usual care** | **Intervention** | **Usual care** | **Intervention** | **Usual care** | |
| **Primary outcome** | | | | | | | |
| Steps 1, 2, 3 or 4 | 5058 | 5292 | 2638 | 2767 | 23.6 | 23.7 | 1.00 (0.81-1.23) |
| **Secondary outcomes** | | | | | | | |
| Steps 2 or 4 | 5058 | 5292 | 865 | 815 | 7.7 | 7.0 | 1.18 (0.92-1.50) |
| Step 2[c] | 4819[d] | 5067[d] | 630 | 594 | 6.4 | 5.8 | 1.27 (0.97-1.66) |
| Steps 1 and 2[c] | 4663[e] | 4888[e] | 516 | 504 | 5.4 | 5.0 | 1.22 (0.90-1.65) |
| Step 4, restricted to living donor transplants[c] | 5058 | 5292 | 235 | 221 | 2.1 | 1.9 | 1.12 (0.77-1.63) |
| Step 4, restricted to pre-emptive living donor transplants[c,f] | 5058 | 5292 | 127 | 115 | 1.7 | 1.5 | 1.11 (0.57-2.15) |

[a] Only the first potential donor was counted for a patient when there were multiple potential donors.

[b] For the adjusted hazard ratio, the referent group was usual care. The primary outcome was analyzed at the patient-level using a stratified, constrained, multistate model accounting for the order in which steps were completed, the clustered design, and the covariates used in the randomization. The historic transplant center referral pattern was a stratification factor in both the randomization and our final model. We also stratified on the different transitions between steps to allow for separate baseline hazard functions. To maintain valid inferences, we used cluster-level bootstrapping to obtain standard errors (accounting for the correlated outcomes within CKD programs) and a t-distribution as a small-sample correction because our trial included <40 clusters. The following baseline characteristics were included in the model: age, sex, Charlson Comorbidity Index, the historic rate of kidney transplant, and whether a transplant center was co-located with the CKD program (present in 6 of the 26 CKD programs). The secondary outcomes were analyzed using the same multistate model framework and evaluation process as for the primary outcome. For outcomes with a single transition, the model reduced to a classic Cox proportional hazards model.

[c] The multistate model for this outcome reduced to a classic Cox proportional hazards model.

[d] Excludes patients who completed step 2 before entering the trial.

[e] Excludes patients who completed steps 1 or 2 before entering the trial.

[f] The follow-up time was censored if and when a patient started dialysis.

© 2023 Garg AX et al. *JAMA Internal Medicine.*

**eTable 18**. Effect of the intervention when restricted to patients who were receiving maintenance dialysis when they entered the trial

Step 1: Referred to a transplant center for evaluation.
Step 2: Had a potential living donor contact a transplant center for evaluation.[a]
Step 3: Added to deceased donor waitlist.
Step 4: Received a transplant from a living or deceased donor.

| Variable | Patients, No. | | Steps completed during the trial, No. | | Rate of steps completed per 100 patient-years | | Adjusted Hazard Ratio (95% CI)[b] |
|---|---|---|---|---|---|---|---|
| | Intervention | Usual care | Intervention | Usual care | Intervention | Usual care | |
| **Primary outcome** | | | | | | | |
| Steps 1, 2, 3 or 4 | 4722 | 5303 | 2696 | 2871 | 26.2 | 24.4 | 1.00 (0.89-1.12) |
| **Secondary outcomes** | | | | | | | |
| Steps 2 or 4 | 4722 | 5303 | 439 | 484 | 4.3 | 4.1 | 1.02 (0.78-1.33) |
| Step 2[c] | 4391[d] | 4962[d] | 293 | 326 | 3.2 | 3.1 | 1.14 (0.83-1.56) |
| Steps 1 and 2[c] | 3165[e] | 3653[e] | 161 | 187 | 2.4 | 2.4 | 1.03 (0.72-1.48) |
| Step 4, restricted to living donor transplants[c] | 4722 | 5303 | 146 | 158 | 1.4 | 1.3 | 1.01 (0.61-1.68) |

[a] Only the first potential donor was counted for a patient when there were multiple potential donors.

[b] For the adjusted hazard ratio, the referent group was usual care. The primary outcome was analyzed at the patient-level using a stratified, constrained, multistate model accounting for the order in which steps were completed, the clustered design, and the covariates used in the randomization. The historic transplant center referral pattern was a stratification factor in both the randomization and our final model. We also stratified on the different transitions between steps to allow for separate baseline hazard functions. To maintain valid inferences, we used cluster-level bootstrapping to obtain standard errors (accounting for the correlated outcomes within CKD programs) and a t-distribution as a small-sample correction because our trial included <40 clusters. The following baseline characteristics were included in the model: age, sex, Charlson Comorbidity Index, the historic rate of kidney transplant, whether a transplant center was co-located with the CKD program (present in 6 of the 26 CKD programs), and the type of dialysis at the time of trial entry (in-center hemodialysis or home dialysis). The secondary outcomes were analyzed using the same multistate model framework and evaluation process as for the primary outcome. For outcomes with a single endpoint, the model reduced to a classic Cox proportional hazards model.

[c] The multistate model for this outcome reduced to a classic Cox proportional hazards model.

[d] Excludes patients who completed step 2 before entering the trial.

[e] Excludes patients who completed steps 1 or 2 before entering the trial.

© 2023 Garg AX et al. *JAMA Internal Medicine.*

**eTable 19**. Rates of kidney transplantation (total, living, deceased) in intervention and usual-care groups in pre-trial, trial, and pre-COVID-19 pandemic periods.

| | Patients, No. | | Patients with outcome, No. | | Rate per 100 patient-years (95% CI)[a] | |
|---|---|---|---|---|---|---|
| | **Intervention** | **Usual care** | **Intervention** | **Usual care** | **Intervention** | **Usual care** |
| **Outcome** | | | | | | |
| Kidney transplant | | | | | | |
| Pre-trial period Nov 1, 2013 to Oct 31, 2017[b] | 8,674 | 9,215 | 1,218 | 1,161 | 6.8 (5.8 – 7.7) | 6.3 (4.7 – 7.9) |
| Trial period Nov 1, 2017 to Dec 31, 2021 | 9,780 | 10,595 | 1,214 | 1,233 | 5.7 (4.7 – 6.6) | 5.3 (4.0 – 6.5) |
| Trial period, pre-COVID Nov 1, 2017 to March 16, 2020 | 7,449 | 8,052 | 766 | 729 | 6.6 (5.5 – 7.6) | 5.7 (4.3 – 7.1) |
| Living kidney donor transplant | | | | | | |
| Pre-trial period Nov 1, 2013 to Oct 31, 2017[b] | 8,674 | 9,215 | 329 | 352 | 1.8 (1.5 – 2.2) | 1.9 (1.3 – 2.6) |
| Trial period Nov 1, 2017 to Dec 31, 2021 | 9,780 | 10,595 | 381 | 379 | 1.8 (1.3 – 2.3) | 1.6 (1.0 – 2.2) |
| Trial period, pre-COVID Nov 1, 2017 to March 16, 2020 | 7,449 | 8,052 | 238 | 225 | 2.0 (1.5 – 2.6) | 1.8 (1.2 – 2.3) |
| Deceased kidney donor transplant | | | | | | |
| Pre-trial period Nov 1, 2013 to Oct 31, 2017[b] | 8,674 | 9,215 | 889 | 809 | 4.9 (4.2 – 5.7) | 4.4 (3.3 – 5.5) |
| Trial period Nov 1, 2017 to Dec 31, 2021 | 9,780 | 10,595 | 833 | 854 | 3.9 (3.3 – 4.4) | 3.6 (2.9 – 4.4) |
| Trial period, pre-COVID Nov 1, 2017 to March 16, 2020 | 7,449 | 8,052 | 528 | 504 | 4.5 (3.9 – 5.1) | 3.9 (3.0 – 4.9) |

[a] The rate was calculated as the number of transplants over the total patient-years. To maintain valid inferences, we used cluster-level bootstrapping to obtain standard errors (accounting for the correlated outcomes within CKD programs) and a t-distribution as a small-sample correction when calculating the margin of error for the 95% CI because our trial included <40 clusters.

[b] Historic transplant rates (pre-trial period) were calculated in a similar fashion as done during the trial period. Patients with advanced CKD (patients approaching the need for dialysis or receiving maintenance dialysis) were accrued on November 1, 2013 or during the period ending September 30, 2017 with a maximum follow up of October 31, 2017.

© 2023 Garg AX et al. *JAMA Internal Medicine.*

**eFigure 1a and 1b**. Multistate model for the intervention and usual-care groups, respectively

The arrows indicate transitions from one state to another. Possible states are: having no steps completed, a single step completed, or multiple steps completed. The different colored arrows indicate the steps completed in the transition:

- An orange arrow indicates a patient was referred to a transplant center for evaluation.
- A blue arrow indicates a patient had a potential living kidney donor contact a transplant center for evaluation.
- A purple arrow indicates a patient was added to the deceased donor waitlist.
- A green arrow indicates a patient received a kidney transplant from a living or deceased donor.

Numbers along the arrows are the number of patients who transitioned from one state to another (and in the brackets is the unadjusted incidence rate per 100 patient-years).

For example, in the diagram for the usual-care group, the two arrows leaving the *Referral* box in Figure 1b (the usual-care group) show that during the trial (*i*) 734 patients with referrals transitioned to having a referral and being waitlisted at a rate of 14.9 events per 100 patient-years and (*ii*) 647 patients with referrals transitioned to having a referral and having a potential living donor start the evaluation process at a rate of 13.1 events per 100 patient-years.

The numbers within each state are:

| | |
|---|---|
| At index: | Number of patients who were in the state at trial entry. |
| Entered: | Number of patients who entered the state during the trial. |
| Left: | Number of patients who left the state during the trial. |

The total number of patients who occupied the state during the trial is the sum of ‘at index’ plus ‘entered’.

The total number of patients in a state at the end of the trial is the sum of ‘at index’ plus ‘entered’ minus ‘left’.

© 2023 Garg AX et al. *JAMA Internal Medicine.*

**1a. Intervention group**

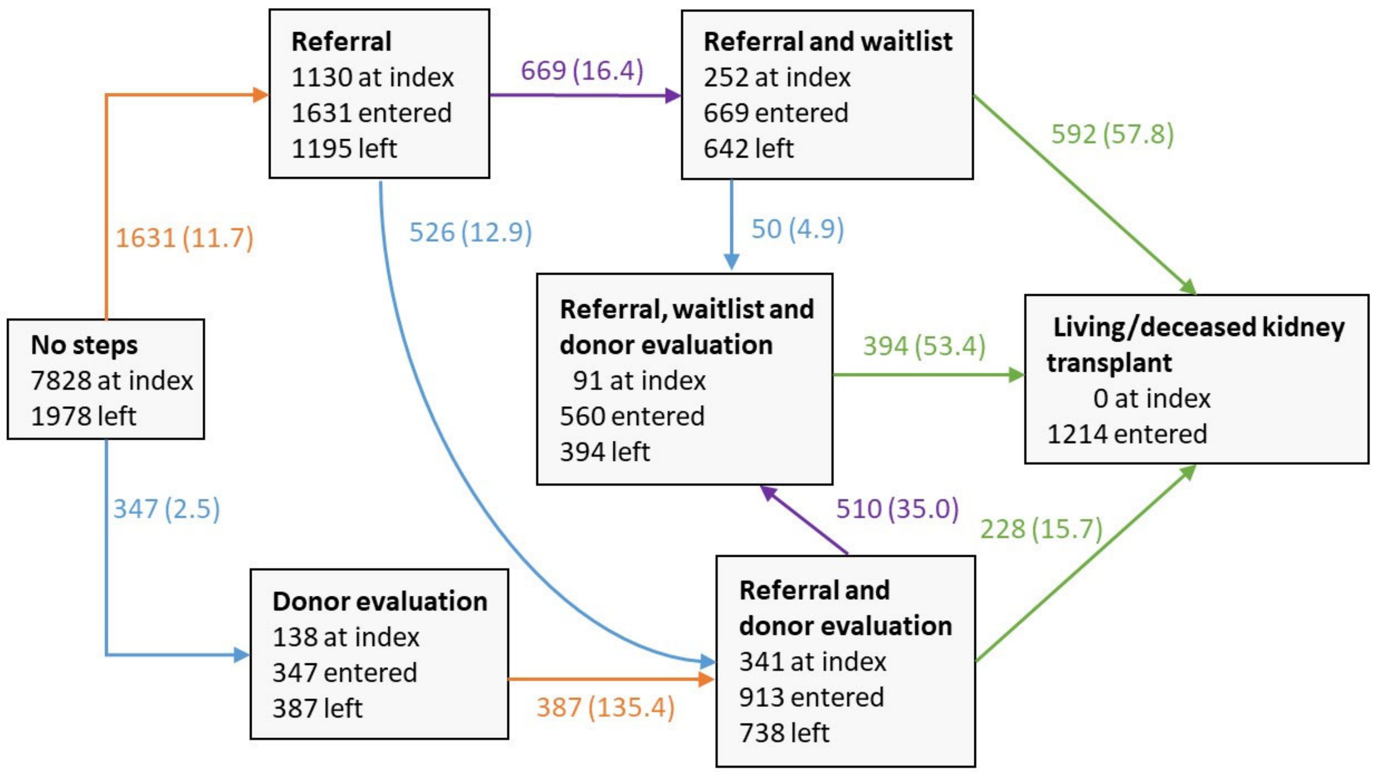


© 2023 Garg AX et al. *JAMA Internal Medicine.*

**1b. Usual-care group**

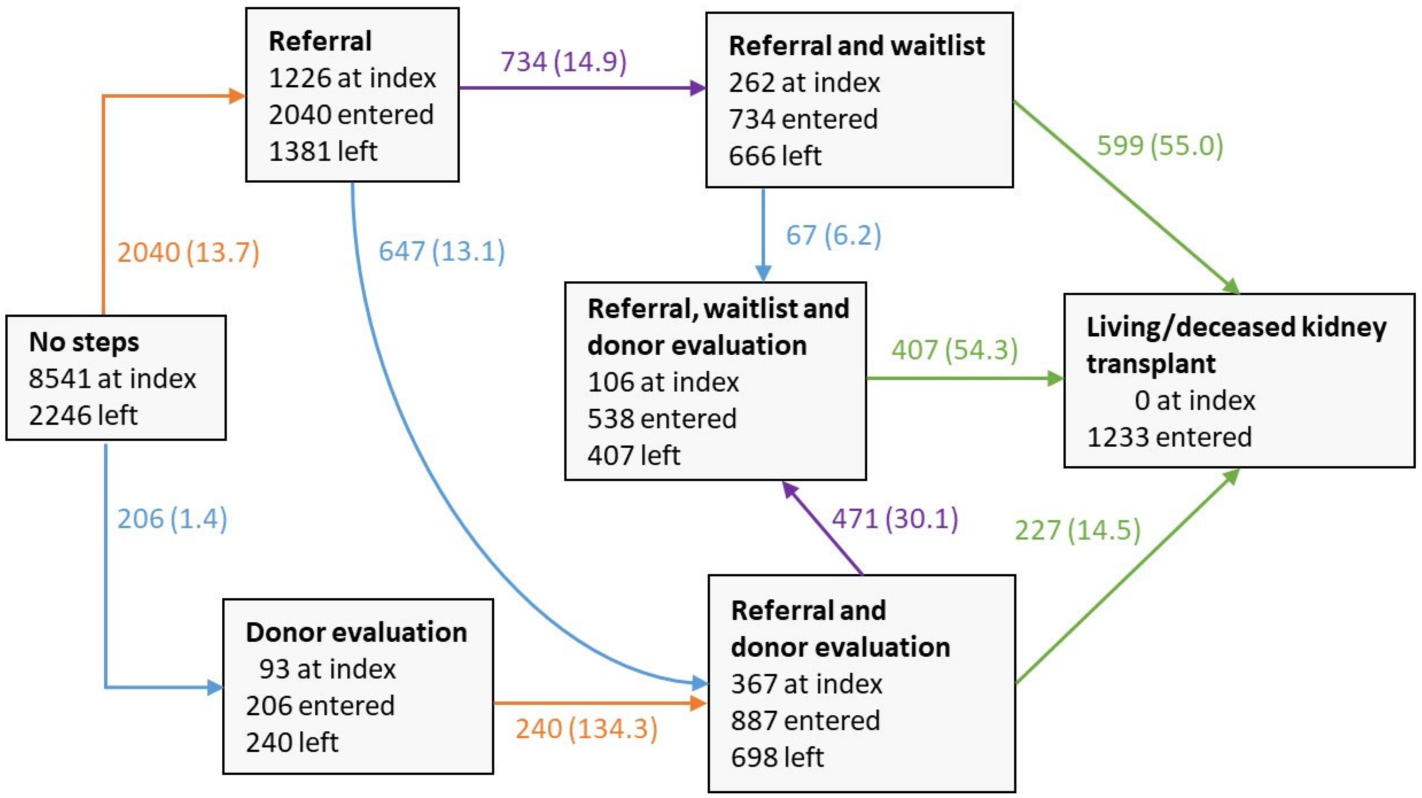


© 2023 Garg AX et al. *JAMA Internal Medicine.*

**eFigure 2**. Forest plot of the effect of the intervention on the primary composite outcome in multiple subgroups

| | | Hazard Ratio (95% CI) |
|---|---|---|
| Primary analysis | | 1.00 (0.87 - 1.15) |
| Type of treatment at trial entry | Approaching the need for dialysis | 1.00 (0.81 - 1.23) |
| | In-center hemodialysis | 1.01 (0.86 - 1.19) |
| | Home dialysis | 0.97 (0.85 - 1.12) |
| Sex | Female | 0.99 (0.82 - 1.20) |
| | Male | 1.01 (0.90 - 1.14) |
| Distance to a transplant center, km | 0 to <30 | 0.94 (0.42 - 2.08) |
| | 30 to <70 | 0.96 (0.76 - 1.21) |
| | 70 and over | 1.04 (0.72 - 1.49) |
| Income quintile | Lower (quntiles 1,2) | 1.03 (0.89 - 1.18) |
| | Higher (quntiles 3,4,5) | 1.00 (0.84 - 1.19) |
| Residential instability | Lower (quntiles 1,2,3) | 1.02 (0.91 - 1.14) |
| | Higher (quntiles 4,5) | 1.00 (0.81 - 1.22) |
| Material deprivation | Lower (quntiles 1,2,3) | 0.99 (0.83 - 1.17) |
| | Higher (quntiles 4,5) | 1.02 (0.87 - 1.20) |
| Ethnic diversity | Lower (quntiles 1,2,3) | 0.98 (0.79 - 1.21) |
| | Higher (quntiles 4,5) | 1.01 (0.88 - 1.16) |
| Dependency | Lower (quntiles 1,2,3) | 0.99 (0.86 - 1.14) |
| | Higher (quntiles 4,5) | 1.02 (0.85 - 1.23) |
| Date entered trial | Nov 1, 2017 | 1.04 (0.91 - 1.17) |
| | After Nov 1, 2017 | 0.96 (0.80 - 1.15) |
| Age, years | 18 to 54 | 0.99 (0.84 - 1.18) |
| | 55 to 65 | 1.02 (0.88 - 1.19) |
| | 66 to 75 | 0.98 (0.77 - 1.25) |
| Diabetes mellitus | Present | 0.99 (0.81 - 1.21) |
| | Absent | 1.00 (0.87 - 1.14) |
| Cardiovascular disease | Present | 1.03 (0.88 - 1.20) |
| | Absent | 0.98 (0.83 - 1.16) |

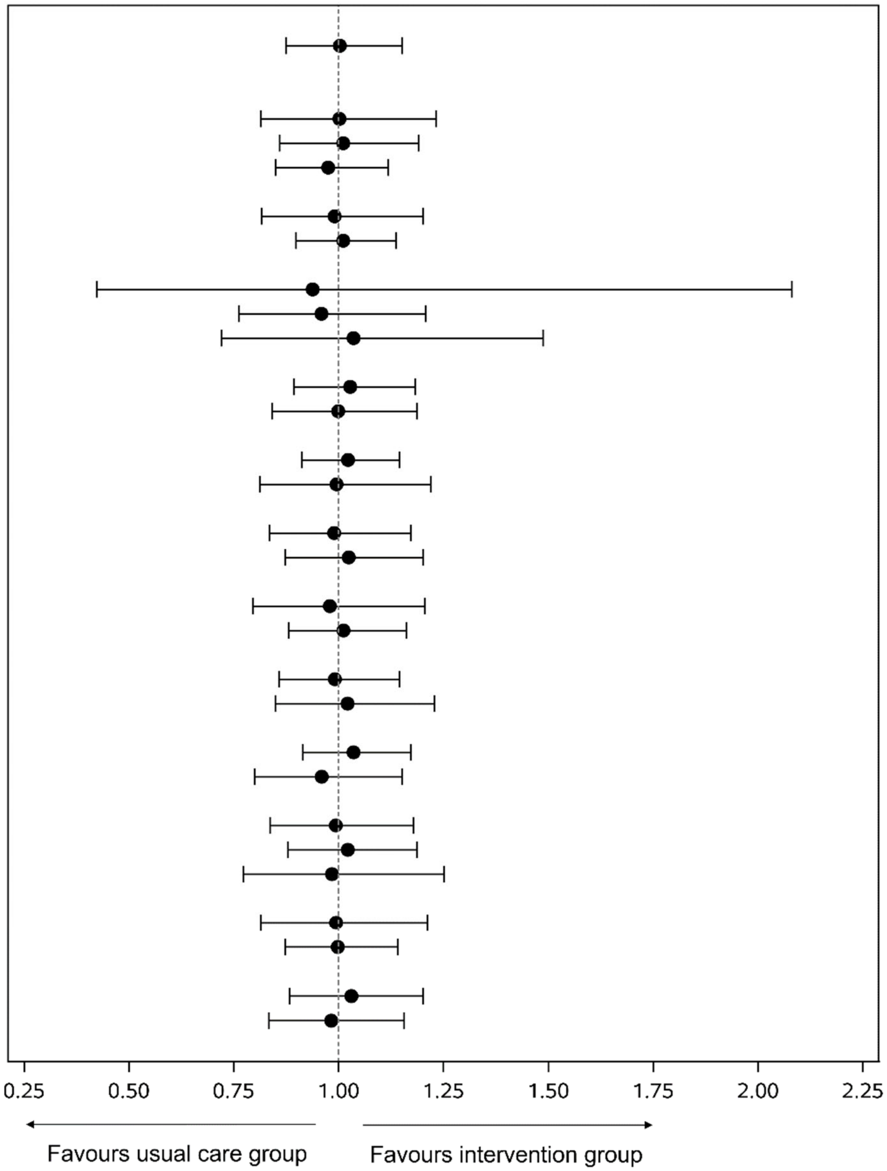


© 2023 Garg AX et al. *JAMA Internal Medicine.*
