# Supplementary material for: Effect of a Novel Multicomponent Intervention to Improve Patient Access to Kidney Transplant and Living Kidney Donation: The EnAKT LKD Cluster Randomized Clinical Trial
Source: JAMA Intern Med. 2023 Nov 3;183(12):1366–75. doi: 10.1001/jamainternmed.2023.5802 (PMC10696487; doi:10.1001/jamainternmed.2023.5802)
Supplement: Supplement 4. — Data Sharing Statement [file jamainternmed-e235802-s004.pdf]

Data Sharing Statement

Garg. Effect of a Novel Multicomponent Intervention to Improve Patient Access to Kidney Transplant and Living Kidney Donation. *JAMA Intern Med*. Published November 03, 2023. doi:10.1001/jamainternmed.2023.5802

Data
Data available: No

Additional Information
Explanation for why data not available: Data Sharing Statement: The provincial datasets used for this trial are held securely in coded form at ICES in Toronto, ON, Canada. While legal data sharing agreements between ICES and data providers (e.g., healthcare organizations and Government) prohibit ICES from making the dataset publicly available, access might be granted to those who meet prespecified criteria for confidential access, available at https://www.ices.on.ca/DAS (das@ices.on.ca). The full dataset creation plan and underlying analytic code might be available from the corresponding author at amit.garg@lhsc.on.ca if requested before Dec 31, 2024, with the understanding that the computer programs might rely on coding templates or macros that are unique to ICES and are therefore either inaccessible or require modification. Links to the full-text study protocol and statistical analysis plan are available at https://clinicaltrials.gov/ct2/show/NCT03329521.
